# Verbing nouns and nouning verbs: Using a balanced design provides ERP evidence against “syntax-first” approaches to sentence processing

**DOI:** 10.1371/journal.pone.0229169

**Published:** 2020-03-13

**Authors:** Lauren A. Fromont, Karsten Steinhauer, Phaedra Royle

**Affiliations:** 1 École d'orthophonie et d'audiologie, Université de Montréal, Montréal, Canada; 2 Centre for Research on Brain, Language and Music, Montreal, Canada; 3 School of Communication Sciences and Disorders, McGill University, Montreal, Canada; Basque Center on Cognition Brain and Language, SPAIN

## Abstract

In this event-related potential (ERP) study we reevaluate syntax-first approaches to sentence processing by implementing a novel paradigm in French that includes correct sentences, pure syntactic category violations, lexical-semantic anomalies, and combined anomalies. Our balanced design systematically controlled for target word (noun vs. verb) and the context immediately preceding it. Group results from 36 native speakers of Quebec French revealed that, up to 300 ms, ERPs elicited by syntactic category violations were comparable with ERP responses to correct sentences, showing that there is no early activation reflecting syntactic category identification. Instead, in response to all anomalous conditions, we observed an N400 followed by a P600. Combined anomalies yielded additive effects of syntactic category and lexical-semantic anomalies on the N400, and a large P600 effect similar to the one observed in the pure syntactic condition. These results provide strong evidence against the hypothesis that (i) syntactic categories are processed first, and (ii) that syntactic category errors “block” lexical-semantic processing. Further, the N400 effect in response to pure syntactic category violations reflects a mismatch detection between a predicted word-stem and the actual target. This mechanism takes place simultaneously (and potentially in parallel) with lexical-semantic processing. In contrast, an interaction of syntax and semantics for the P600 reveals that the same neurocognitive resources are recruited for syntactic and semantic integration, both promoted by the implementation of an acceptability judgement task in our design. Additional analyses of individual data complemented these observations: during sentence processing, participants did not rely on one single cognitive mechanism reflected by either the N400 or the P600 effect but on both, suggesting that the biphasic N400-P600 ERP wave *can* indeed be considered to be an index of phrase-structure violations in most individuals, at least if they are realized on content words.

## 1. Introduction

Sentence comprehension, as effortless as it seems, is contingent upon a rapid analysis of each incoming word along several linguistic dimensions. Within a few hundred milliseconds, we identify the syntactic category of each word in order to build larger phrases (e.g., a noun is combined with its determiner to build a determiner phrase) and use its lexical-semantic properties to integrate it with the sentence and discourse contexts. Event-related potentials (ERPs), thanks to their excellent temporal resolution, allow us to study the time course of semantic and syntactic processing during on-line sentence comprehension. However, despite decades of research, the relative timing of cognitive processes underlying these two linguistic dimensions is still controversial. Friederici’s serial and modular account of sentence processing adopts a “syntax-first” approach [1,2] where syntactic category identification of each incoming word occurs first and conditions further lexical-semantic and morphological analyses on that word. While this framework has largely dominated the field since the mid 1990s, it has faced contradictory data [3–5] in addition to a critical review of most previous studies that had supported the “syntax-first” approach [6]. In that review, Steinhauer and Drury (2012, hereafter S&D) outline major methodological flaws observed in the majority of ERP studies investigating syntactic category and lexical-semantic processing and call for cautious interpretation of their ERP results. Following S&D’s arguments, here we investigate the hypothesis that experimental designs are potentially responsible for the contradictory evidence for syntax-first models and propose a novel and improved design to reevaluate the time-course of syntactic-category identification and lexical-semantic processing. By doing so, we will (1) clarify the time course of syntax-semantics integration, (2) elucidate the functional significance of N400 and P600, and (3) methodologically challenge recent claims about the complementarity of these two components in online sentence processing.

### 1.1. On the precedence of syntactic category identification

Friederici’s “syntax-first” approach [1,7–8] is arguably the most influential ERP-based neurocognitive model of language processing to date. It posits three phases, defined here along with the ERP components that index them:

- Between 100 and 300 ms after stimulus onset, automatic identification of syntactic categories (noun, verb, etc.) takes place to generate initial syntactic representations. This phase is labelled *local phrase-structure building*.
  - The early left anterior negativity (ELAN), appears around 150 ms after stimulus presentation in response to syntactic category violations (henceforth SCVs) [7].
- Between 300 and 500 ms after stimulus onset, lexical-semantic information and morpho-syntactic relations are processed in parallel.
  - The N400 component is a negative deflection peaking around 400 ms after stimulus onset, usually observed at centro-parietal sites (e.g., [9,10]). The N400 has generally been associated with lexical-semantic processing: more specifically, and important for our design, it is sensitive to both priming effects (i.e., when the target appears after a related word–the prime–its retrieval is facilitated; priming leads to a reduction of the N400), and cloze probability (i.e., the probability of the target word completing a particular sentence; lower cloze probability increases the N400);
  - The left anterior negativity (LAN) is a negative wave that peaks around 400 ms, often with a left-lateralized and frontal distribution (in the visual modality). It has been observed in response to morpho-syntactic errors, such as agreement violations [11–13].
- Between 500 and 1000 ms after stimulus onset, information from various processing streams (e.g., syntactic and semantic information) are integrated, and, if necessary, a revision of the first analysis is initiated.
  - The P600 component is a large, long-lasting posterior positivity, starting around 500 ms, that is elicited by syntactic violations [14], as well as syntactically complex or temporarily-ambiguous sentences [15]. Some suggest that the early P600, observed between 500 and 750 ms–and distributed over midline electrodes [13]–reflects the reactivation of contextual information in order to process syntactic integration. The late P600, observed at parieto-occipital sites between 750 and 1000 ms or beyond, is assumed to reflect general sentence reanalysis and repair [16] as well as controlled processes related to decision-making and categorization, as it is highly modulated by the presence or absence of a task [17] and by experimental design [18].

This model predicts that in Phase I, syntactic category information enjoys a special status and is used prior to lexical-semantic information for sentence comprehension. The ELAN effect that is specifically observed in response to syntactic category errors is the cornerstone of Friederici’s proposal [1]. This effect is time-locked to the syntactic category cue at word onset or offset, for example inflectional or derivational morphology [19–21], and reflects automatic linguistic processes that are putatively independent from attention [18] and task demands [22]. However, ELAN effects are not robust: for example, less than half of the studies reviewed by S&D report one. These authors report major methodological issues associated with ELAN studies. In particular, ELANs have been mostly evidenced using one specific context manipulation paradigm [7,23] (e.g., *Die Bluse wurde oft* *ge**bügelt*, ‘The blouse was often ironed’ vs. Die Bluse wurde am *geb*ügelt*, ‘The blouse was on-the *ironed’ [24] target words or morphemes are underlined) which became a standard paradigm for ELAN studies. Insertion of the preposition-article contraction *am* (‘on-the’) creates an SCV on the underlined target past-participle verb (purportedly indexed by the participle verb prefix *ge-*) because a noun would be expected rather than a verb, resulting in an ELAN effect on the target word. According to S&D, context-manipulation paradigms can lead to artefactual context effects in the time-window of the target word. For example, an N400 difference in the baseline window is observed when comparing a context ending with a content word in the correct condition (*oft* ‘often’), to a context ending with a function word in the SCV condition (*am* ‘on-the’). Baseline correction would then shift the whole ERP waveform in the correct condition towards a positivity, and in the incorrect one towards a negativity, leading to an early, sustained negativity in the violation condition [25]. To compensate for this problem, many studies adopted a post-target onset baseline (0–100 ms, [18,19,22,26]). However, using this type of baseline does not rule-out potential artefacts (especially in auditory studies) and rather ensures the appearance of a component at 100 ms. Despite these concerns, studies have continued to adopt this standard paradigm (e.g., [27,28]) which has serious potential to affect baselines.

Friederici emphasizes that Phase I in her model does not simply reflect the earlier temporal availability of syntactic category information: it also predicts that this information is *obligatorily* integrated first, and that it reliably guides later phases of language processing, such as lexical-semantic integration. Thus, without a grammatical syntactic representation that includes the current target word, Stage II, which includes semantic and morphosyntactic processing, cannot proceed. According to Friederici and collaborators, this claim is strongly supported by the absence of an N400 for combined syntactic and semantic violations (e.g., Die Wolke wurde am *geb*ügelt*, ‘The cloud was on-the *ironed’), a phenomenon known as “semantic blocking”. Semantic blocking is viewed as strong evidence in favour of the syntax-first model, independent of the ELAN. In fact, semantic blocking has been found even in studies that did not observe an ELAN effect [29].

However, it is crucial to note that, as S&D point out, the standard paradigm used by Friederici and collaborators fails to create outright syntactic or semantic violations at the time when the target word is presented. First, contrary to what has been claimed, the prefix *ge-* is not a reliable marker for verb syntactic category: most past participles in German can be used as adjectival modifiers, and thus can appear after the preposition+determiner *am* ‘on-the’ (see S&D). Therefore, the occurrence of an ELAN at the prefix *ge-* cannot be associated with a failure in phrase-structure building due to a SC violation, because there is none. Following this logic, there cannot be any lexical-semantic incongruency at the target word either, since *geb**ügelt* should be integrated as an adjective modifying a subsequent noun that has not yet appeared (and not the preceding subject NP *the cloud*). The participant must wait for the following noun to integrate lexical-semantic information, thereby explaining the absence of an N400 at the target word.

As is the case for the ELAN, the semantic blocking effect has not always been replicated [4,30–33]. For example, in some studies N400s have been observed in response to SCVs when the participants were instructed to ignore syntax [22]. Perhaps the strongest argument against semantic blocking comes from a replication of the original semantic-blocking study [24] using the exact same German sentence materials illustrated above, which tested seven different groups of German native speakers while varying task instructions, linguistic profiles, and the types of filler sentences used, in order to elicit the ELAN and semantic-blocking effects [33]. Across all groups, a significant N400 was not only observed for combined syntactic and semantic errors, but even for pure SCVs, suggesting that not only was the N400 not blocked, but that pure syntactic violations alone *can also* elicit N400s, at least when realized on a content word. Note however that the N400 for the pure semantic violation condition (e.g., *Die Wolke wurde oft* ?*geb**ügelt*, ‘The cloud was often ?ironed’) was the one with the largest amplitude (similar to [24]), followed by the combined and pure syntactic anomalies. In other words, there were no additive effects of semantic and syntactic N400s in the combined anomaly condition. The slightly larger N400 in response to the combined violation compared to the pure syntactic anomaly could be interpreted as the result of priming effects between the subject and the target word in the pure syntactic condition [6]. On the other hand, the uncertainty of how the target word should be categorized–either as an adjective to be integrated with the upcoming noun or as a verb to be integrated with the previous subject–may explain why the N400 is smaller in the combined condition compared to the pure semantic one. Two other studies in Mandarin Chinese adopted a target-manipulation approach on the verb, rather than using a pre-verbal context-manipulation one (i.e., the standard paradigm), to investigate semantic blocking effects in the absence of morpho-syntactic cues for syntactic categories [30,31]. In object–subject–verb sentences the target verb would either be correct, semantically anomalous, or a combined semantic and syntactic category anomaly (that is a noun instead of a verb: e.g., Real estate business—corporation—recent several years—develop / *condition_[noun]_−[…] ‘This corporation has developed/*condition_[noun]_ its real estate business […] during several recent years’), an N400 was observed in the combined condition, and therefore no semantic blocking was evidenced [31]. Similar results have been obtained using passive sentences and systematically manipulating for syntactic category and semantic incongruency in Mandarin, where an N400 effect was also observed for “pure” SCVs (e.g., ‘That piece of glass is carefully wiped / *dishcloth […]’ [32]. However, in these two studies, target nouns were always associated with a syntactic violation condition while target verbs were always correct, resulting in unbalanced designs that may affect ERPs independent of syntactic category processing in addition to promoting participant strategies.

### 1.2. What are we left with? LAN and N400 responses to syntactic category violations

One the one hand, there is some evidence in favour of an elegant, but very possibly incorrect, syntax-first model of sentence processing, and on the other, there is evidence pointing against it–but this evidence is not consistent. That is, in some cases, LAN effects have been elicited by syntactic category errors, suggesting that LANs (rather than ‘ELANs’) may reflect grammatical processing difficulties at large, including agreement and SCV effects [3,28,34]. In other cases, SCVs seem to elicit N400s [30–32,35]. If S&D are right and there is no reliable early marker for syntactic category identification before 300 ms, then it seems especially important for a model of sentence processing to account for discrepancies between LAN and N400 findings, which would now be viewed as the earliest ERP markers for SCVs. Zhang and colleagues [31] offer a cross-linguistic explanation for the presence of an N400 in response to SCVs in their studies on Mandarin. They suggest that, due the absence of morpho-syntactic cues in Mandarin, speakers rely more heavily on lexical-semantics for sentence processing, thus inducing an N400 in ungrammatical structures. In contrast, the presence of such cues would promote morpho-syntactic processes, thus eliciting an ELAN or a LAN, as found in German. However, we have seen that N400s have also been found in response to SCVs with no semantic incongruency in a large-scale replication study in German [33], which makes this cross-linguistic explanation less compelling.

The interpretation of the LAN effect as a defining marker of morpho-syntactic processing of local relationships (agreement in particular [1,25,36], but also syntactic category identification [3,28]) has been controversially discussed by Molinaro and colleagues [13] on the one hand, and Tanner and colleagues [37] on the other. In a series of studies specifically looking at individual ERP patterns in response to agreement violations, Tanner and colleagues [38–40] provided evidence that, in their dataset, the LAN effect was largely the product of grand-averaging over multiple subjects. That is, the apparent LAN in grand average data resulted from component overlap between central N400s elicited by some participants and right-posterior P600s found in *different* individuals. These two overlapping components cancelled each other out at central and posterior electrodes near the midline, and only left-lateralized portions of the N400 survived, thus resembling a LAN in the group average. Moreover, when plotting individual N400 magnitudes against P600 ones, Tanner et al. [40] observed a negative correlation between these two measures. This correlation revealed that most individual ERP profiles did not display a biphasic (negativity+P600) profile (only 2/42 did), but instead tended either toward a P600 profile (31/42 participants) or an N400 profile (observed in only 9/42 participants). The authors suggest that the N400 may index mismatches between word- and morphological-form based predictions and the actual linguistic input in English speakers, whereas P600s reflect a distinct combinatorial processing strategy that is not prediction-driven.

The alternative account in the LAN/N400 debate does take the LAN effect at face value [13,41], while adopting (for the LAN) a similar predictive approach to the one suggested by Tanner for the N400. Somewhat reminiscent of Zhang et al’s cross-linguistic argument discussed above [33], Molinaro and colleagues [13] propose that the LAN and N400 are at two points on a continuum reflecting a mismatch with predicted features. Importantly, however, linguistic features can be expressed in more or less transparent ways, even within the same language. The more participants can rely on transparent morpho-phonological cues to process syntactic mismatches (e.g., the third person -*s* in English *walks / walk*), the more likely they will elicit a LAN, and the more they have to focus on more elaborate lexical information (e.g., the word stem mismatch *is* / *are*), the more N400-like their response. Molinaro et al.’s functional distinction between N400s and LANs implies that LANs are real, and not just averaging artifacts as claimed by Tanner and colleagues. This implication has received strong support from a recent 80-participant analysis by Caffarra and colleagues that does observe genuine LAN effects in response to agreement violations in 55% of participants (only 25% show an N400 effect), and in 49% of individual trials [42].

While the N400/LAN discussion largely arose from studies of morpho-syntactic agreement processing (e.g., subject-verb, gender and number agreement, and tense marking), it may be relevant to our present study on SCVs. As many studies that did not employ Friederici’s standard paradigm for SCVs have observed either a LAN [4,34,43] or an N400 [30–32], rather than an ELAN, the aforementioned debates could shed some light on the disparity between N400 and LAN effects observed in response to SCVs as well.

### 1.3. Individual variability in ERP responses to morphosyntactic errors

When Tanner and colleagues observed distinct individual ERP profiles expressed as an N400 or a P600 dominance, they introduced the *Response Dominance Index* (RDI [40]) as a new measure reflecting the relative dominance of either the N400 (negative values for the RDI) or the P600 effect (positive values). They observed that individual RDIs were correlated across experimental manipulations (e.g., subject-verb or tense agreement). In other words, if an individual showed an N400 dominance for subject-verb agreement errors, this participant would likely also be N400 dominant in response to tense-agreement errors. It follows that some individuals might rely more on lexically-based mechanisms (reflected by negativities), while others rely on combinatorial processes (indexed by the P600 effect) to process agreement, a pattern also found in response to syntactic category violations [33].

An important methodological issue related to RDI measures and especially to correlations between N400 and P600 amplitudes has to do with component overlap. In all previous studies, amplitude quantification for both components was done (i) in the same (centro-parietal) region of interest and (ii) using adjacent time-windows, that is the P600 window (e.g., 500–900 ms) directly followed that of the N400 (e.g., 300–500 ms). Since individual ERP components vary in latency and do not abruptly change at 500 ms, these correlations may at least to some extent result from component overlap: early parts of P600s would thus contaminate the N400 time-window and late parts of the N400 would in turn affect the P600 interval [44]. Alternatively, this phenomenon could simply be explained by autocorrelation, which would be promoted by the selection of subsequent time windows on the same electrodes. These points are important because systematic interdependence between N400 and P600 effects would also call for a reconsideration of most neurocognitive models of sentence comprehension that posit a biphasic detection-reanalysis pattern [40] and assume that the N400 and the P600 reflect fundamentally distinct–and largely independent–cognitive processes.

The first objective of the present study is to re-evaluate the syntax-first approach to sentence processing by implementing a balanced design that controls both for contexts and targets. Second, this study aims to shed light on the nature of LAN vs. N400 negativities as indices of morpho-syntactic and lexical-semantic processing. Third, our data will allow us to test the (in)dependence between processes indexed by those negativities and the P600, while considering inter-individual variability.

### 1.4. The current study

This study uses an improved, novel paradigm that systematically manipulates contextual semantic priming and target-word syntactic category. The general idea was to create word-category violations by replacing a noun with a verb (and vice versa), while ensuring that context words immediately preceding the target noun or verb were optimally matched, in order to avoid any ERP baseline issues that rendered previous studies invalid (S&D). Nouns and verbs were selected as targets in the SC violation to maximize the comparability of our data with previous studies, but also because content words allowed us to include some additional manipulations that would specifically test the semantic blocking hypothesis ([24,45] vs. S&D). We selected two types of French sentence structures as carriers for our two types of target words. One carrier sentence (see Example 1.a below, experimental sentence in **bold font**) included a “control” verb (such as *oser* ‘to dare’) that requires an infinitive verb phrase as its complement (e.g., our target verb *plaquer* ‘to-tackle’ in Example 1.a). The other carrier phrase (in 1.b) included a transitive verb (such as *ôter* ‘to-remove’) which mandatorily requires a noun phrase as its complement (e.g., *le* *crapaud* ‘the toad’). Note that “control” and “transitive” do not reflect a linguistic dichotomy. All target nouns were directly preceded by a definite determiner (here: *le* ‘the_[masc]_’), the presentation interval of which would later be used to calculate the pre-target baseline for the ERP analysis. In order to ensure a comparable baseline in the verb condition (1.a), the target verb (*plaquer* ‘to-tackle’) was preceded by a clitic pronoun (*le* ‘him’) that served as the target verb’s direct object and, importantly, was a homograph of the determiner in the noun condition (i.e., *le plaquer* ‘to-tackle him’). Thus, conditions 1.a and 1.b included target nouns and target verbs in a grammatical sentence, and both target words were immediately preceded by the exact same written stimulus (*le*). In both experimental sentences, the target words were followed by the same 3-word prepositional phrase (here: *sur le côté* ‘to/on the side’) that would address spill-over effects and delay sentence final (wrap-up) effects in the ERPs (see S&D for details). Finally, in order to introduce a pronoun antecedent and to license the use of definite determiners, we included context sentences (shown in unbolded font below) that preceded the experimental sentences. These context sentences also contained a semantic prime (shown in upper case font) for the respective target word in the experimental sentence (e.g., swamp is a prime for ‘toad’ in Example 1.b).

1.a *Marie et Jeanne jouent au hockey avec leur copain. **Elles osent le****plaquer****sur le côté***.

Mary and Jane are playing hockey with their friend_[m.sg]_. **They**_**[f.pl]**_ **dare him**_**[m..sg]**_ **tackle** **to the side.**

‘Mary and Jane are playing hockey with their friend. They dare to tackle him to the side.’

1.b. *Marie et Jeanne vont au* *marais* *avec leur copain*. ***Elles ôtent le*** ***crapaud*** ***sur le côté***.

Mary and Jane go to the swamp with their friend_[m.sg]_. **They**_**[f.pl]**_ **remove the**_**[m.sg]**_ **toad** **on the side.**

‘Mary and Jane go to the swamp with their friend. They remove the toad on the side.’

The full experimental design with example stimuli is illustrated in Table 1. We introduced outright SCVs by swapping the target words (e.g., ‘they remove the *to-tackle’, second row in Table 1), and manipulated semantic priming by interchanging context sentences so the target remains unprimed (e.g., prime: hockey; target: toad, third row in Table 1). Pairs of context sentences and sentences containing unprimed target words made sense in isolation, but taken together they resulted in lexical-semantic anomalies, so we will refer to unprimed sentences as *semantically anomalous*. In total, four main conditions involving both noun and verb targets were created by manipulating these two dimensions: syntactic category (Syntax: correct/incorrect), and lexical-semantic anomalies (Semantics: primed/unprimed). These were combined using a Latin-square design. Sentence contexts and targets appeared in every condition, and all sentence pairs were matched on relevant psycholinguistic factors, presented in §2.2.

**Table 1:** Sample stimuli for the four experimental main conditions, including both noun and verb targets.

		Context sentence	Experimental sentence			
Syntax	Semantics		Subject pronoun + Verb	Clitic / Determiner	Target	Prepositional phrase
✓	✓	*Marie et Jeanne jouent au hockey avec leur copain*.	*Elles osent*	*le*	*plaquer*	*sur le côté*.
		Mary and Jane are playing hockey with their friend_[M]_.	They_fem_ dare	him	to-tackle	on the side.
		*Marie et Jeanne vont au marais avec leur copain*.	*Elles ôtent*	*le*	*crapaud*	*sur le côté*.
		Mary and Jane are going to the swamp with their friend_[M]_.	They_[F.PL]_ remove	the_[M]_	toad_[M]_	on the side.
✘	✓	*Marie et Jeanne vont au marais avec leur copain*.	*Elles osent*	*le*	*crapaud	*sur le côté*.
		Mary and Jane are going to the swamp with their friend_[M]_.	They_[F.PL]_ dare	him	*toad.masc	on the side.
		*Marie et Jeanne jouent au hockey avec leur copain*.	*Elles ôtent*	*le*	*plaquer	*sur le côté*.
		Mary and Jane are playing hockey with their friend_[M]_.	They_[F.PL]_ remove	the_[M]_	*to-tackle	on the side.
✓	✘	*Marie et Jeanne vont au marais avec leur copain*.	*Elles osent*	*le*	?*plaquer*	*sur le côté*.
		Mary and Jane are going to the swamp with their friend_[M]_.	They_[F.PL]_ dare	him	?to-tackle	on the side.
		*Marie et Jeanne jouent au hockey avec leur copain*.	*Elles ôtent*	*le*	?*crapaud*	*sur le côté*.
		Mary and Jane are playing hockey with their friend_[M]_.	They_[F.PL]_ remove	the_[M]_	?toad_[M]_	on the side.
✘	✘	*Marie et Jeanne jouent au hockey avec leur copain*.	*Elles osent*	*le*	*crapaud	*sur le côté*.
		Mary and Jane are playing hockey with their friend_[M]_.	They_[F.PL]_ dare	him	*toad.masc	on the side.
		*Marie et Jeanne vont au marais avec leur copain*.	*Elles ôtent*	*le*	*plaquer	*sur le côté*.
		Mary and Jane are going to the swamp with their friend_[M]_.	They_[F.PL]_ remove	the_[M]_	*to-tackle	on the side.

F = feminine, M = masculine, PL = plural

#### Hypotheses

In the 100–300 ms time-window of the target word, Friederici’s syntax-first model would predict an ELAN effect in ERPs for all SCVs regardless of semantics (e.g., ‘The cloud was on-the *ironed’), compared to the correct conditions. Following S&D, and because of our balanced design, we predict no difference between syntactically correct sentences and those with SCVs (i.e., Syntax: incorrect, rows 2 and 4 in Table 1) in this early time-window. Regarding the 300–500 ms time-window, several predictions can be made. First, Friederici’s model would predict an N400 effect for lexical-semantic anomalies in otherwise grammatical sentences (e.g., ‘The cloud was often ?ironed’), but *not* for the combined violations (e.g., ‘The cloud was on-the *ironed’) due to semantic blocking. In contrast, we predict an N400 effect in *both* these conditions (Semantics: unprimed, rows 3 and 4), as SCVs are not expected to block lexical-semantic processing. Second, as LANs and N400 effects have also been observed in response to SCVs, even though the experimental manipulations are different from that of agreement studies, we will investigate whether the discussion on LAN-N400 effects can be extended to syntactic category identification. We tentatively hypothesize that Tanner would predict either an N400, or the “illusion” of a LAN effect in group data due to component overlap of N400 and P600 components across participants [37]. On the other hand, if we adopt Molinaro’s idea of a continuum between LAN and N400 effects [13], and since French has transparent morpho-phonological markers for syntactic category (e.g., infinitive verbs end with–*er*,–*ir*, or–*re*), we might expect SCVs to elicit LAN effects reflecting participants’ use of these markers to detect anomalies. Third, if the cognitive processes underlying syntactic category and lexical-semantic processing are independent, we predict additive effects of our syntactic and semantic manipulations in this time-window [46]. On the other hand, if these processes rely on (and compete for) the same neurocognitive resources, we predict an interaction of these two factors on the ERP responses [47]. After 500 ms, most–if not all–frameworks would predict P600 effects at least for all conditions involving SCVs (Syntax: incorrect, lines 2 and 4). According to some studies, P600 modulations may also be expected for semantic manipulations (e.g., [48]). If so, similar predictions regarding additivity versus interactions between syntactic and semantic effects apply as outlined for the 300–500 ms time-window. We will assess these predictions while taking inter-individual variability into account, following Molinaro and colleagues’ recommendations for analyzing the data using mixed-effect models [41], and further explore our individual datasets following Tanner et al.’s practice [38–40]. The latter analyses should reveal if the amplitudes of P600s and preceding negativities are correlated, and whether such correlations are partly due to component overlap. In other words, our data are expected to not only clarify certain predictions of Friederici’s influential model, but also shed light on a number of methodological issues in recent ERP research.

## 2. Methods

### 2.1. Participants

Thirty-nine native speakers of Quebec French (20 of which identified as women) aged 20–31 (*M* age: 25;10) participated in the study. All were right-handed (confirmed with an abbreviated French version of the Edinburg handedness questionnaire [49]), had normal or corrected-to-normal vision, and reported no neurological disorder. Given the highly multilingual environment in Montreal, participants filled in a language usage questionnaire where they were asked to evaluate their daily exposure to French at work or school, in the family, social circles, and through media use. All of the participants had limited exposure to English (*M % daily exposure* = 11.41, SD = 10.69), and reported having learned English as part of their education program only (*M age of first exposure* = 12.44, SD = 1.94). Additional native French speakers were recruited to evaluate our stimuli in three separate offline experiments: an acceptability rating study (*n* = 40), a relatedness rating questionnaire (*n* = 68), and a cloze test (*n =* 40).

### 2.2. Stimuli

In total, 160 pairs of correct sentences as in 1.a. and 1.b. were created. In order to minimize inter-item variability, we controlled for the three following dimensions: (1) phonological and lexical properties of context and target words, (2) acceptability ratings for correct and anomalous conditions, and (3) degree of semantic priming between primes and target words. Being aware that phonological structure may result in differences in the early ERP time-windows (that may then be misinterpreted as ELANs, see S&D), and that lexical properties can affect the N400 component, we matched (i) control and transitive verbs in the carrier sentences, and (ii) target verbs and nouns, on both phonological/orthographic and lexical dimensions. Paired t-tests revealed that main verbs and targets did not differ significantly in number of phonemes, syllables, and log-transformed frequency (details about the main verbs can be found in the S1 Table and about the targets in the S2 Table). Second, any difference in acceptability within pairs in the correct or the anomalous conditions could also result in unwanted ERP differences. Focusing on correct sentences and ‘combined’ anomalies, we distributed all 160 sentence pairs into four experimental lists, using a Latin square design, so that every participant would only see one sentence per pair and condition. Forty undergraduate students were asked to rate their acceptability on a scale from 1 (acceptable) to 4 (unacceptable). Results showed that, although our correct sentences were judged more acceptable than our anomalous sentences (*p* < .001), there was no effect of target lexical category (noun or verb, *p* = .514) nor interaction between these two factors (acceptability*lexical category: *p* = .196). We thus ensured that no major lexical differences within sentence pairs could affect acceptability judgements or electrophysiological responses during the online experiment. Third, we wanted to control for priming effects on target nouns and verbs, as more priming would result in a reduced N400 in the correct condition. We thus sought to match target nouns and verbs in their degree of priming. A questionnaire assessing relatedness between primes and targets in the correct sentences (from 1: not related to 5: extremely related) revealed no difference between noun and verb targets, despite highly related prime-target pairs in the case of verbs (for all conditions *M* = 3.35, *SD* = 1.07, *p* > .1). However, we also used a cloze test to evaluate the predictability of target words given the context sentence in both correct and semantically anomalous conditions, and computed a cloze probability index ranging from 0 (never predicted by our participants) to 1 (always predicted). Cloze probability indices for semantic anomalies were, as expected, always zero, so we used the responses on the correct sentences for our comparisons. Paired t-tests revealed that verb targets (*M* = .1) were less predictable than nouns (*M* = .17, *p =* .006) despite low predictability levels overall. Priming results and methods are summarized in the supplementary materials (S3 Table). Since it was impossible to control for this specific dimension without interfering with other lexical, formal, acceptability, and relatedness dimensions that we had carefully controlled for, we opted to include cloze probability as a random slope in our statistical models (see §3.2). It is important to note that the experiment is designed so that results on target nouns and verbs will be merged to perform the analyses: this is key to avoid context effects that have previously led to baseline issues, as well as any possible lexical differences on target words (S&D).

Creating the various violation conditions based on the original set of 160 grammatical sentence pairs resulted in a final set of 1280 items, with an average of 13.24 words per item (*SD =* 1.12; range for context sentences: 5–10 words; number of words for target sentences = 7), corresponding to an average duration of 6622 ms per trial (*SD* = 560 ms). The 1280 items were divided into four lists using a Latin square design, such that each participant would read one single list with 320 sentences (80 per main condition), but no prime or target word would ever be repeated within a given list. To every list, we added the same set of 80 filler sentences that were either correct or contained one or more subject-verb number-agreement error(s) unrelated to the present study, such that each participant read a total of 400 experimental sentences with their corresponding contexts.

### 2.3. Experimental procedure

Participants sat in a chair 80 cm in front of a computer monitor and read the sentences while their electroencephalogram (EEG) was recorded. Each trial began with the presentation of a fixation cross (500 ms). The sentences were presented in rapid-serial-visual presentation mode (each word: 300 ms display + 200 ms blank screen), and words appeared in white 30-point Arial font on a black background. At the end of every trial, a “???” prompt appeared and remained on the screen until participants scored sentence acceptability on a scale of 1 to 5 (1: totally acceptable– 5: totally unacceptable) by pressing a button on a response box. After participants responded, a second prompt “!!!” would appear for a 1800 ms interval during which participants were encouraged to blink their eyes (in order to reduce eye blinks during sentence presentation). The experimental sessions started with two short practice blocks containing six sentences each, while the 400 experimental trials were divided into eight blocks of 50 trials each, separated by short breaks. The recording lasted about 2.5 hours including setup. Participants provided signed informed consent to participate in the experiment. This study was approved by the Research Ethics Office (Institutional Review Board) of the Faculty of Medicine, McGill University, and *Comité d’éthique de la recherche en santé*, *Université de Montréal*.

### 2.4. EEG recording and data processing

EEG was recorded continuously from 25 Ag-Cl active shielded electrodes mounted on an EEG cap (Waveguard^TM^ original, *ANT Neuro*, Netherlands) according to the 10–20 system [50] at the following sites: Fp1, Fpz, Fp2, F7, F3, Fz, F4, F8, T3, C3, Cz, C4, T4, T5, P3, Pz, P4, T6, O1, Oz, O2. All EEG electrodes were referenced online against the right mastoid. An electrode placed halfway between Fpz and Fz served as ground. Impedances were kept below 5 kΩ.

Data were analyzed using *EEGLab* [51] and *ERPLab* [52]. Continuous data were re-referenced offline to linked mastoids, and bandpass-filtered with .1 and 40 Hz cut-off frequencies (IIR Butterworth filter). After epoching the data from -200 to 2000 ms relative to the onset of the target word, we rejected data that exceeded a peak-to-peak threshold of 75 μV (in 100 ms steps). Three participants were excluded due to excessive artefacts (over 50% in at least one of the eight sub-conditions), and one for not respecting instructions. The remaining 36 participants had 11.5% rejected trials on average, with no differences among the eight sub-conditions, *F*(7,217) = 1.836, *p* = .101, Greenhouse-Geisser corrected. Finally, the epoched data was corrected using a 200 ms pre-target baseline. Based on previous findings and visual inspection of our own ERP data, we selected four analysis time-windows corresponding to the following components: ELAN (100–300 ms), N400 (350–500 ms), early P600 (550–650 ms), and late P600 (800–1200 ms). These time windows correspond to those previously used in the literature, while their separation by at least 50 ms minimized the risk of component overlap. For the N400, we also ran analyses for the (most commonly used) 300–500 ms time window, which replicated all effects reported for 350–500 ms.

### 2.5. Statistical analyses

All statistical analyses were done using *R* version 3.4.2 (*Short summer* [53]). Since we are interested in whether syntactic category identification interacts with lexical-semantic processing at both performance and electrophysiological levels, we implemented a Syntax×Semantics (2×2) design in both behavioural and ERP analyses.

#### Behavioural data analysis using cumulative link mixed effects models

Acceptability ratings on a Likert scale (from 1: totally acceptable to 5: totally inacceptable) were analyzed with cumulative link mixed effects models using the *clmm* function from the *ordinal* package [54]. Cumulative link models are more appropriate than parametric statistical tests (e.g., linear regressions) when analyzing ordinal data such as Likert scales [55]. Considering these ratings as continuous variables in statistical tests can be problematic. Because Likert scales are limited on the edges, they lead to a “censoring” effect (in our case, participants cannot select 0 or 6) [56]. This limitation brings the mean closer to the center and decreases variability in the data, both of which can affect the statistical test. Second, points on the scale are not always conceived as continuous by participants. Cumulative links models assume that the ratings from 1 to 5 are ordered, but not that the five points on the scale are equidistant, or that the values beyond these points are interpretable. For a given factor, the estimate is calculated compared to the baseline level–here, the variables have been coded such that the correct condition is the baseline. A positive estimate therefore expresses a higher rating on the scale. To ensure convergence, the random effect structure was limited to random slopes for condition per participant. We fit a model that included Syntax, Semantics (two levels, correct and anomalous), and their interaction.

#### ERP data analysis using mixed effects models

Response-contingent ERP analyses were run using mixed effect models (packages: *lme4* and *lmerTest* [57,58], because they can adjust for repeated measures of both participants and items while controlling for individual variability, and handle missing data and unequal sample sizes better than traditional ANOVAs [59]. ERP effects on the midline and lateral sites were analyzed separately. The variables were coded such that the intercept corresponded to the correct condition. The maximal random structure ensuring convergence included random slopes for condition per participant, condition per item, and cloze probability per item. We first calculated main effects and interactions for factors Syntax, Semantics, Anteriority (two levels: using F and C electrodes as anterior sites; and P and O electrodes as posterior sites), and Hemisphere (two levels: left and right). Then, we decrementally removed interactions and factors from this full model until we reached the optimal model, determined by comparing two minimally different models using ANOVAs. For clarity of interpretation, we used ANOVA wrappers (Type III Wald chi-square test) with the *car* package [60]–note that this option was not available using *clmm* for the behavioural analyses. When needed, we performed follow-up analyses of interactions and post-hoc pairwise comparisons using the *emmeans* package [61], using the default multiple comparison adjustment (Dunnett adjustment).

## 3. Results

### 3.1. Behavioural data

Sentences with either a lexical-semantic anomaly or an SCV were judged to be less acceptable than correct sentences (*M* = 2.02, *SD* = 0.47), as supported by the strong main effects of Syntax and Semantics in Table 2. Further, the interaction between Syntax and Semantics and the follow-up analyses show that lexical-semantic anomalies primarily affected judgments in the absence of a syntactic anomaly (*M* = 3.09, *SD* = 0.61). In contrast, sentences containing an SCV were judged equally unacceptable regardless of whether lexical-semantic anomalies were present (*M* = 3.93, *SD* = 0.67) or not (*M* = 3.71, *SD* = 0.73).

**Table 2:** Effects of syntactic category violations and semantic anomalies on participants’ responses. Cumulative Link Mixed Model fitted with the Laplace approximation.

		Effects	Estimate*	SE	z-value	p-value
Main model		Syntax	3.099	0.251	12.34	< 0.001
		Semantics	1.971	0.179	10.98	< 0.001
		Syntax×Semantics	-1.525	0.155	-9.84	< 0.001
Follow-up pairwise comparisons	Semantics correct	Syntax	1.508	0.027	56.13	< 0.001
	Semantics anomalous	Syntax	0.624	0.031	20.34	< 0.001
	Syntax correct	Semantics	0.938	0.027	34.60	< 0.001
	Syntax incorrect	Semantics	0.054	0.028	1.91	< 0.06

In the main model, the estimate is calculated in comparison to the rating for the correct condition.

### 3.2. ERP data

**Fig 1:**
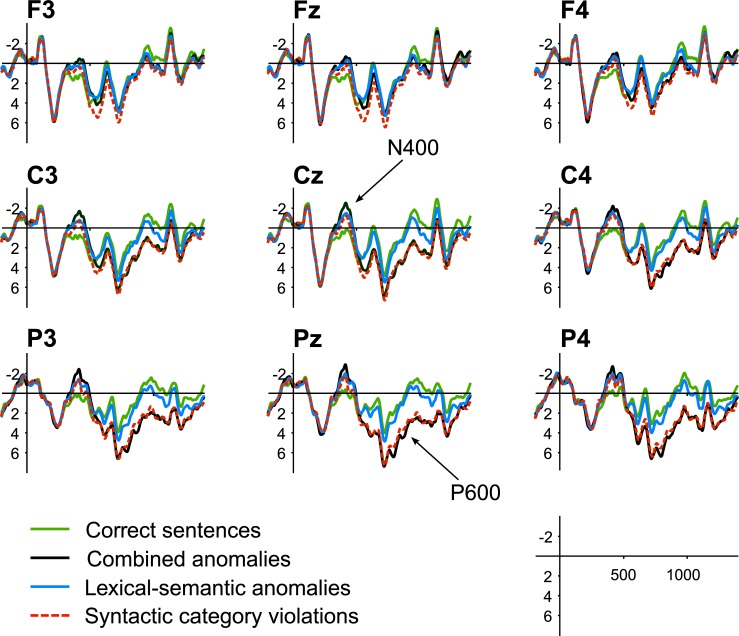
Grand average waveforms for the four experimental conditions. ERPs are shown time-locked to target word onset for correct sentences (green line), sentences with a syntactic category violation (red dotted line), sentences with a semantic anomaly (blue line), and sentences with combined anomalies (solid black line) on nine representative electrodes. Target onset is indicated by the vertical bar, where tick bars represent 2 μV of activity; time-windows extend from -200 ms to 1300 ms relative to target onset.

**Fig 2:**
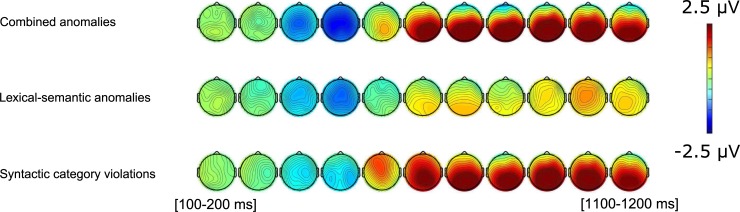
Voltage maps illustrating the effect of each of the three anomalous conditions. Mean amplitudes measured between 100 ms and 1200 ms in 100 ms increments.

The ERP waves for the correct condition and each of the three anomalous conditions (SCVs, lexical-semantic anomalies, and combined anomalies) are illustrated in Figs 1 and 2. All ERP waves are time-locked to target word onsets: verb and noun targets are merged, thus canceling out any possible context or target effects. Visually, two observations are striking. First, we observe the absence of the ELAN: our balanced design and the care with which we selected and controlled the stimulus materials led to virtually aligned ERP onsets when using a 200 ms pre-target baseline correction. Note that our pre-stimulus baselines themselves are also comparable across all conditions, contrary to most SCV studies that use a “post target onset” baseline (0–100 ms) due to unbalanced designs [18,19,22,26,62]. Second, we observe an N400 in both SCV conditions. We will investigate these observations by running statistical analyses on the 100–300 ms time-window (where one would predict an ELAN effect) and the 350–500 ms time-window for the N400 effect. Later effects are also observable: a posterior positivity in both the SCV and the combined conditions between 500 and 1200 ms, and a frontal positivity between 500 and 650 ms in the pure SCV condition only. We ran mixed-effect models on each of these time-windows and report significant main effects and interactions involving factors Syntax or Semantics in Table 3. These effects will be commented in text, along with follow-up analyses when necessary.

**Table 3:** Analysis of deviance table (Type III Wald chi-square tests) reporting significant effects corresponding to the main mixed-effect models in the four time-windows of interest (ELAN, N400, early positivity, posterior P600), at midline electrodes and lateral sites.

Time- window	Site	Fixed effects and interactions	Chi-square	Df	p-value	mR^2^ / cR^2^
100–300 ms	Midline	(Intercept)	65.097	1	< 0.001	.002 / .100
	Lateral sites	(Intercept)	50.978	1	< 0.001	.001 / .108
350–500 ms	Midline	(Intercept)	19.633	1	< 0.001	.014 / .112
		Syntax	6.623	1	0.01	
		Semantics	27.085	1	< 0.001	
	Lateral sites	(Intercept)	34.180	1	< 0.001	.013 / .165
		Syntax	8.546	1	0.003	
		Semantics	22.890	1	< 0.001	
550–650 ms	Midline	(Intercept)	27.403	1	< 0.001	.008 / .116
		Syntax	21.971	1	< 0.001	
		Syntax×Semantics×Anteriority	6.088	1	0.014	
	Lateral	(Intercept)	31.970	1	< 0.001	.006 / .154
		Syntax	12.705	1	< 0.001	
		Syntax×Semantics×Anteriority	5.481	1	0.019	
800–1200 ms	Midline	(Intercept)	0.229	1	0.632	.018 / .119
		Syntax	16.544	1	< 0.001	
		Syntax×Anteriority	13.554	1	< 0.001	
		Syntax×Semantics×Anteriority	6.098	1	0.014	
	Lateral	(Intercept)	1.327	1	0.249	.017 / .094
		Syntax	9.226	1	0.002	
		Semantics	4.227	1	0.040	
		Syntax×Semantics	3.860	1	0.049	
		Syntax×Anteriority	64.223	1	< 0.001	
		Syntax×Hemisphere	4.763	1	0.029	
		Syntax×Semantics×Anteriority	4.398	1	0.036	

*mR*^*2*^: marginal R squared, *cR*^*2*^: conditional R? squared. Note that the variance explained by the mixed-effect models is quite small. Fromont and collaborators discuss this issue further and offer alternatives for ERP data analysis [70].

#### Absence of ELAN: 100–300 ms

Visual inspection of the data (Figs 1 and 2) indicates no early anterior negativity elicited by SCVs, as the onset components in all four conditions are nicely aligned until around 300 ms. There was no statistically significant effect of Syntax, Semantics, and no interaction involving these terms in this early time-window.

#### Main effects of Syntax and Semantics on the N400: 350–500 ms

**Fig 3:**
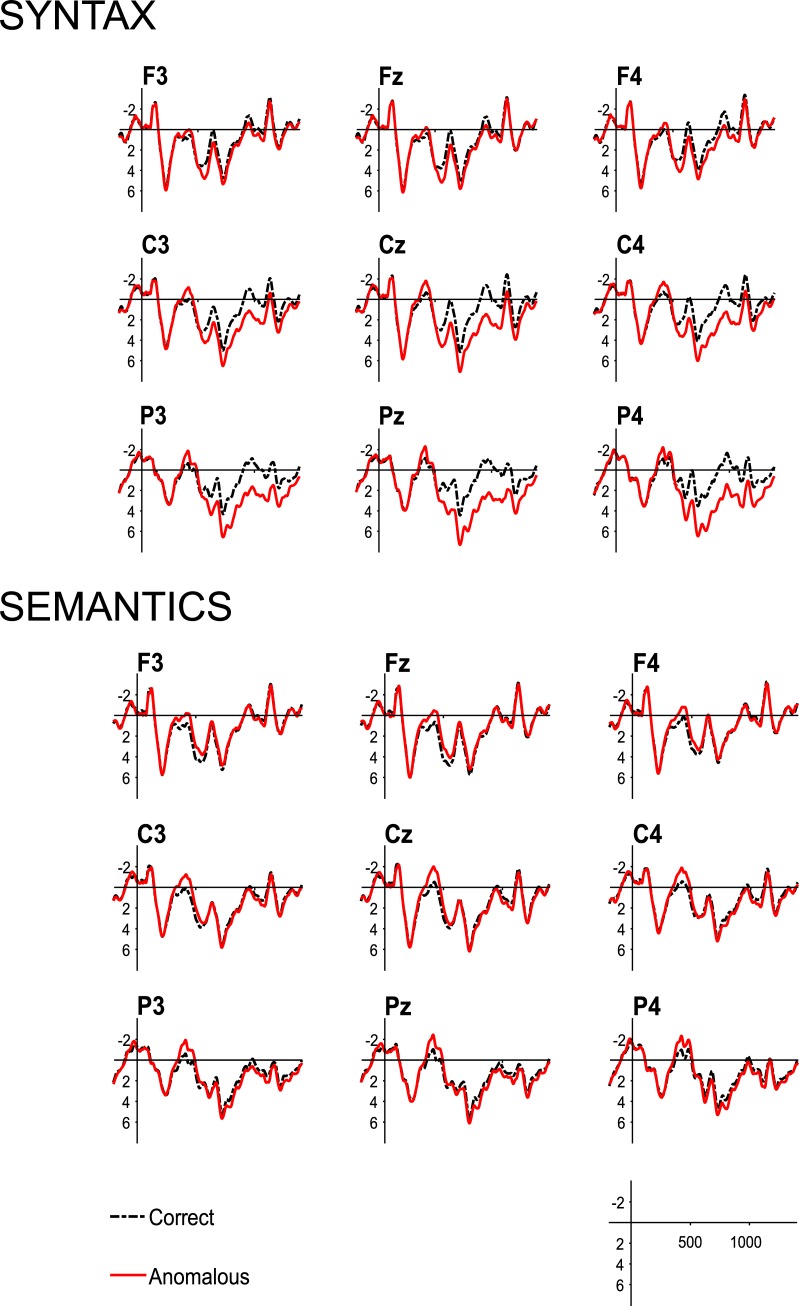
Grand average waveforms illustrating the main effects of Syntax and Semantics. Anomalous trials are in red and correct ones in black. Target onset is indicated by the vertical bar, where tick bars represent 2 μV of activity; ticks marks on the horizontal line represent 500 ms of time.

Visual inspection of the data in the 350–500 ms time-window revealed a centrally distributed negativity elicited by both SCVs and lexical-semantic anomalies, while the combined syntactic and lexical-semantic anomaly condition elicited a larger N400 than the “pure” syntactic and semantic conditions. At both midline and lateral sites, the best model fit included main effects of Syntax and Semantics without any interaction involving these factors (Fig 3), suggesting that a broadly distributed N400 was elicited under both violation types, with an additive effect of SCVs and lexical-semantic anomalies. We further tested if the N400 effects elicited by SCVs and lexical-semantic anomalies have different scalp distributions by calculating the difference waves between each of these two conditions and the correct one, and running a new model with factors Condition (2 levels: lexical-semantic anomalies, SCVs) and Electrode (21 levels). To calculate those difference waves, we had to average over items, so the random structure was highly simplified to random slopes for participants per condition, making the model much less conservative, and maximizing the chance of getting significant effects. Even so, there was no effect of Condition and no interaction between Condition and Electrode.

We further examined modulations of the N400 responses by selecting a subset of three electrodes best representing N400 effects (C3-Cz-C4). First, to check for the existence of additive effects for semantics and syntax that we appear to observe in the main models, we investigated Electrode (three levels) and Condition effects (four levels). We calculated difference waves between each of the three anomalous conditions minus the correct condition. We also included a fourth artificial “additive” condition that was calculated by adding N400 effects of the lexical-semantic and syntactic difference waves. As expected, the N400 effect observed in the combined condition was greater than the N400 in the lexical-semantic and pure syntactic conditions alone, but indistinguishable from the “additive” condition. The mixed-effect model (including random slopes for Condition per Participant) revealed a main effect of Condition (*X*^2^ (1) = 25.37, *p* < .001). Follow-up pairwise comparisons revealed that the estimated marginal means for lexical-semantic (-1.31 μV) and syntactic anomalies (-0.91 μV) were significantly smaller than those for combined anomalies (-1.86 μV; combined vs. lexical-semantic: *t*(407) = 3.366, *p =* .005; combined vs. syntactic: *t*(407) = 5.884, *p <* .001) and also, importantly, than those for artificial “additive” anomalies (-2.22 μV; additive vs. lexical-semantic: *t*(407) = 5.659, *p <* .001; additive vs. syntactic: *t*(407) = 8.176, *p <* .001). Conversely, there was no significant difference between the (empirical) combined and the (calculated) “additive” conditions (combined vs. additive: *t*(407) = -2.292, *p =* .1). Together with the main analysis, this result suggests that the two effects are indeed additive.

Second, in order to better understand how priming effects modulate the N400 response in different conditions we explored whether cloze probability had an impact on the N400 amplitude under syntactic and semantic manipulations. We ran a mixed-effects models with factors Syntax, Semantics, and Cloze probability. Interestingly, we observed not only main effects of Syntax (*X*^2^(1) = 9.56, *p* = .002), Semantics (*X*^2^(1) = 32.12, *p* < .001), and Cloze (*X*^2^(1) = 5.69, *p* = .017), but also an interaction between Cloze and Semantics (*X*^2^(1) = 6.8, *p* = .009). A regression tree from the *partykit* package [63] was chosen for a follow-up analysis in this specific case, because this method illustrates better how continuous and categorical variables interact: this is absent from pairwise comparisons using *emmeans*. However, this method is less conservative, as it does not take random factors into account. The regression tree confirmed that while semantically anomalous sentences induced a larger N400 amplitude than primed sentences (p < .001), cloze probability modulated the semantically primed sentences regardless of SCV. The N400 elicited by the target had a smaller (less negative) amplitude when the target was highly probable (cloze probability above .4, *p =* 0.001) compared to less probable target words (all full models can be found in S2_Appendix). Note however that this analysis is exploratory because we did not explicitly control for cloze probability, therefore the distribution of cloze probability values is largely skewed towards zero.

#### SCV effects on the early positivity: 550–650 ms

Inspection of the four individual conditions suggests that violations that are purely syntactic elicit a positivity that appears at frontal sites as early as 550 ms and becomes posterior around 650 ms, while combined anomalies only elicit a posterior positivity. These observations were reflected by a main effect of Syntax at midline and lateral sites, and a significant Syntax×Semantics×Anteriority interaction. As separate follow-up analyses at anterior and posterior sites did not converge, we split our data by levels of Semantics. Both models showed marginal interactions for Syntax×Anteriority (Semantics correct: *X*^2^(1) = 3.32, *p* = .07, Semantics incorrect: *X*^2^(1) = 2.80, *p* = .09). Visual inspection of Figs 1 and 2 suggest that the more frontal positivity in response to “pure” SCVs, but not in the combined anomalies, is driving the three-way interaction in the main model, and that the two trends in the follow-up reflect distinct patterns. However, we remain cautious about this tentative interpretation in the absence of significant results in the follow-up analyses.

#### Late P600: 800–1200 ms

Two patterns are of interest when considering our results together with the ERP waves in Fig 1 and the voltage maps in Fig 2, namely (a) a large P600 effect for SCV (irrespective of semantic anomalies), and (b) a small P600 elicited by pure lexical-semantic anomalies. At midline sites, there was a main effect of Syntax (*p* < .001), and interactions between Syntax×Anteriority (*p* < .001) and Syntax×Semantics×Anteriority (*p* = .015). At lateral sites, we observed a similar main effect of Syntax (*p* = .002), a main effect of Semantics (*p* = .04), and interactions of Syntax×Anteriority (*p* < .001), Syntax×Semantics (*p* = .049), Syntax×Semantics× Anteriority (*p* = .036), and Syntax×Hemisphere (*p* = .029). For each model, separate follow-up models for anterior and posterior sites confirmed that SCVs elicited a large, mostly-posterior positivity (midline at anterior sites: *X*^2^(1) = 21.07, *p* < .001; midline at posterior sites: *X*^2^(1) = 42.27, *p* < .001; lateral electrodes at anterior sites: *X*^2^(1) = 10.51, *p* = .001; lateral electrodes at posterior sites: *X*^2^(1) = 37.75, *p* < .001). We further explored the interactions involving Syntax×Semantics by splitting into levels of Syntax (correct, incorrect). Main effects of Semantics were observed for syntactically correct sentences, suggesting a small broadly-distributed positivity in response to lexical-semantic anomalies (midline: *X*^*2*^(1) = 3.69, *p =* .05; lateral electrodes: *X*^*2*^(1) = 4.43, *p* = .03). Significant interactions of Semantics×Anteriority were observed for syntactically incorrect sentences (midline: *X*^2^(1) = 8.25, *p* = .004; lateral electrodes: *X*^2^(1) = 10.65, *p* = .001), potentially revealing a slightly larger posterior positivity for combined anomalies compared to pure SCVs (SCVs effect at midline: Anterior–Posterior = -0.98 μV, z = -5.67, *p* < .001; Combined anomalies effect at midline: Anterior–Posterior = -1.69 μV, z = -9.67, *p* < .001; SCVs effect at lateral electrodes: Anterior–Posterior = -0.56 μV, z = -6.89, *p* < .001; Combined anomalies effect at lateral electrodes: Anterior–Posterior = -0.8 μV, z = -9.57, *p* < .001). Note however that comparing between pure SCVs and combined anomalies at Anterior and Posterior levels did not reveal any significant differences.

To summarize our findings so far, both semantic and syntactic anomalies seem to have elicited N400s and P600s, with seemingly additive effects on the N400 and interactive effects on the P600. The next section will investigate whether these biphasic ERP profiles in the grand-average data were representative of the individual data.

### 3.3. Exploratory analyses of individual data

Our analyses revealed N400 effects (and no LAN effect) in response to both syntactic violations and lexical-semantic anomalies. The N400 was followed by a large P600 effect only for sentences with an SCV. Visual inspection of individual data, however, revealed some interindividual variability: as shown in previous studies, individuals seem to display different dominance toward either an N400 or a P600 profile [33,40]. In this section, we evaluate whether (1) larger N400s equate to smaller P600s across individuals (i.e., whether the N400 and P600 are negatively correlated), and (2) whether their ERP responses are similar across conditions (i.e., whether N400s and P600s observed in response to one condition each correlated with N400 and P600 effects in *other* conditions).

#### Correlations between N400 and P600 effects within each condition

**Fig 4:**
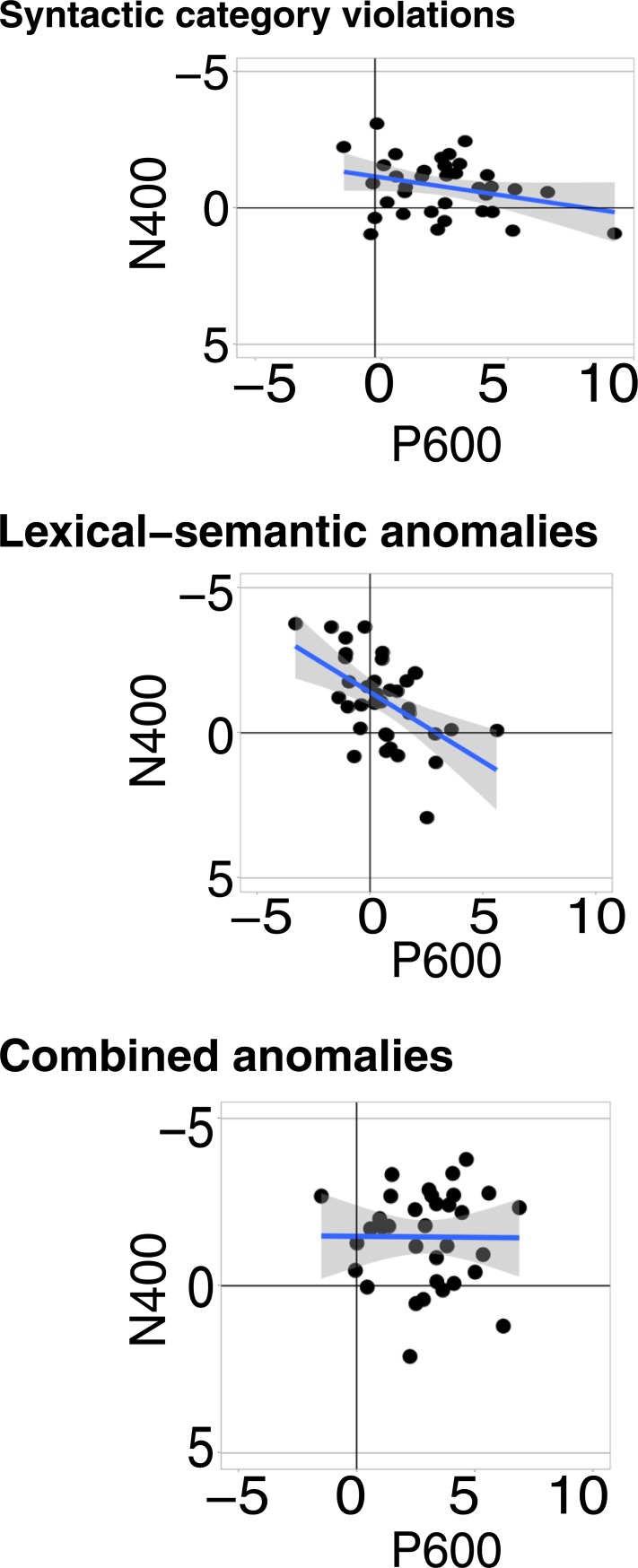
Scatterplots showing the relationship between N400 and P600 effects across individuals in the three anomalous conditions. For each participant, we subtracted the average response (in μV) to SCVs, lexical-semantic anomalies, and combined anomalies minus the correct condition. Linear smooths are fitted with a 95% confidence interval.

The magnitudes of N400 and P600 effects were estimated for every individual by calculating the difference between each of the three anomalous conditions (SC, lexical-semantic, and combined anomalies) minus the correct condition and by quantifying the amplitudes of these difference waves in representative time intervals. We then calculated the correlation between N400 and P600 amplitudes within all three conditions (using *Hmisc* [64]). As we were concerned that component overlap or autocorrelation may contribute to spurious correlations, both here and in previous studies [44], we ran two analyses. The first one adopts the method promoted by Tanner and colleagues, i.e., using the same region of interest (C3-Cz-C4-P2-Pz-P4) to quantify both N400 and P600 amplitudes, and using time-windows that were almost adjacent (50 ms apart), i.e., 350–500 ms for the N400 and 550–650 ms for the (early) P600. As expected, in this analysis we observed significant negative correlations in the SC (*r* = -.61, *p* < .001) and lexical-semantics conditions (*r* = -.55, *p* < .001), and a marginal but non-significant correlation in the combined condition (*r* = -.32, *p* = .057). The second analysis aimed to minimize component overlap and allowed us to test our hypothesis that component overlap or autocorrelation may have inflated previous findings. A first difference with previous approaches was that the regions of interest were optimized and limited to electrodes where each component was most prominent: the N400 was measured at C2-Cz-C3 electrodes and the P600 at P2-Pz-P3. A second difference was that the time-windows were selected 300 ms apart (N400: 350–500 ms; P600: 800–1200 ms). As illustrated in Fig 4, a significant negative correlation was observed *only* in the lexical-semantic condition (*r* = -.54, *p* < .001), which remained relatively unchanged compared to the first analysis. For the syntactic conditions (which elicited much larger P600s) the absence of a correlation between N400 and P600 amplitudes seems to confirm that component overlap or autocorrelation may have played a role in our first analysis (SCVs: *r* = -.3, *p =* .08; combined: *r* = -.09, *p* = 0.60).

Next, we addressed whether participants elicited similar responses throughout the experiment: for example, whether a participant who elicited a large N400 in responses to lexical-semantics anomalies also showed a large N400 effect in the two other conditions. This pattern could be taken as an indicator that ERP components largely reflect individual strategies independent of experimental manipulations. We therefore compared the response magnitudes of the N400 and P600 elicited across conditions [33].

#### Correlations for N400 and P600 effect magnitudes across conditions

**Fig 5:**
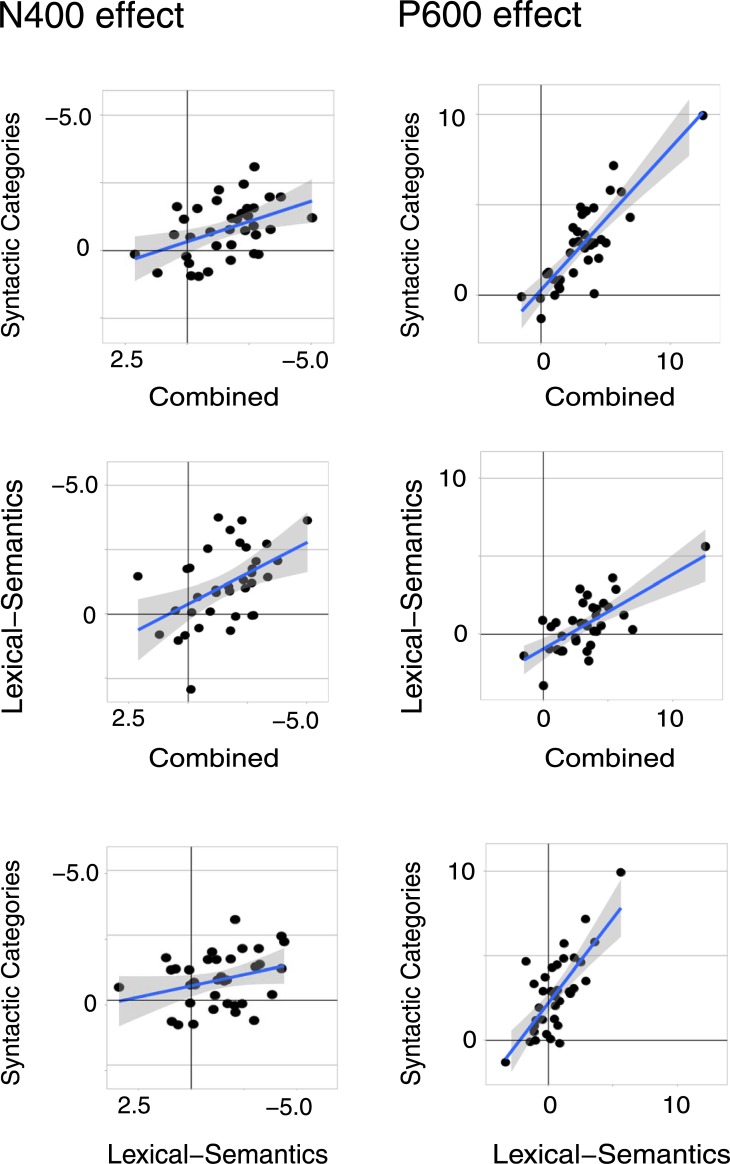
Scatterplot showing the relationship of N400 and P600 effects across individuals between conditions. We plotted the average response (in μV) for SCVs against combined anomalies, lexical-semantic anomalies against combined anomalies, and SCVs against lexical-semantic anomalies. Linear smooths are fitted with a 95% confidence interval.

We calculated correlations for the N400 and the P600 effects between all possible pairs of our three violation conditions. When appropriate, we compared the correlation coefficients using a test statistic that compares two correlation coefficients based on dependent groups with one overlapping variable (*cocor* [65, 66]). All results are illustrated in Fig 5. The N400 effects correlated positively when comparing SC and combined anomalies (*r* = 0.44, *p* = 0.007), as well as lexical-semantic and combined anomalies (*r* = 0.48, *p* = 0.003), but no significant N400 correlation was found when comparing “pure” SC and lexical-semantic anomalies (*r* = 0.3, *p* = 0.076). There was no difference between these coefficients, even when comparing the highest (*r* = 0.48) and the lowest one (*r* = 0.3; z = -1.089, *p* = 0.276), probably because the correlation was moderate even when significant. On the other hand, P600 effects were highly correlated across all conditions: SC and combined anomalies (*r* = 0.83, *p* < 0.001); lexical-semantic and combined anomalies (*r* = 0.69, *p* < 0.001); and SC and lexical-semantic anomalies (*r* = 0.73, *p* < 0.001).

## 4. Discussion

### 4.1. Consequences for “syntax-first” approaches to sentence processing

**Fig 6:**
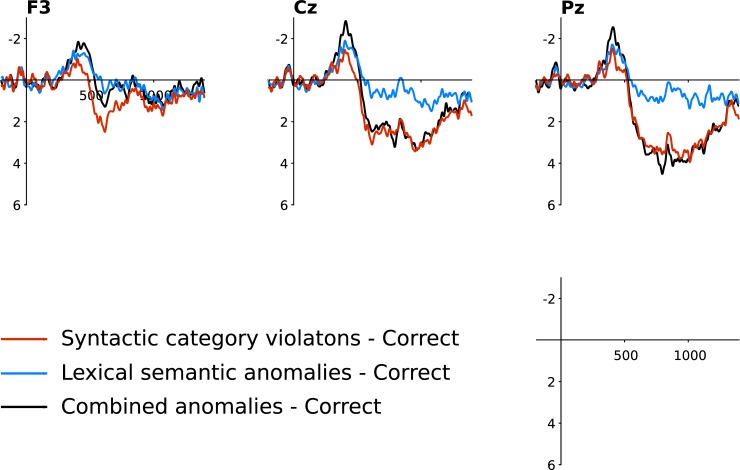
Difference waves between each of the anomalous conditions minus the correct condition. The effects of SCVs (in red), lexical-semantic anomalies (in blue), and combined anomalies (in black) are illustrated on three representative electrodes: F3 (where no ELAN is found), Cz (for the N400), and Pz (for the P600). Target onset is indicated by the vertical bar, where tick bars represent 2 μV of activity; ticks marks on the horizontal line represent 500 ms of time.

The present study reevaluates the temporal organization of syntactic category and lexical-semantic processing, and demonstrates that syntactic category violations do not elicit early ERP responses (previously described as ELANs [1,7,23]), nor do they block lexical-semantic information processing. To compare and contrast our findings with Friederici’s model, we summarize the time-course of ERP effects in response to SCVs, lexical-semantic, and combined anomalies in Fig 6.

Our study is one of the few studies to employ outright SCVs [34,67]. In studies employing the standard paradigm introduced by Friederici and colleagues [1,7,18,24], SCV sentences could always be rescued by adding morphological or lexical information after the target (e.g., Die Bluse wurde am *geb*ügelt*, ‘The blouse was on-the *ironed’ → *Die Bluse wurde am* *gebügelt****en*** *Jackett befestigt*, ‘The blouse was pinned to-the ironed jacket’) [6]. In our experiment, we use transitive or control verbs that specifically select for either a noun or a verb, respectively, such that swapping targets automatically results in outright SCVs. We argue that our data are unambiguous in that they provide a strong argument against the primacy of syntactic category identification (and, by extension, phrase-structure building), over other types of information processing during online sentence processing.

It has been argued that the ELAN effect is a reliable marker of syntactic processing in auditory studies but is not always expected in visual paradigms even when using the same linguistic materials [29]. That is, the absence of an ELAN in a reading study can still be argued to be unsurprising as it has been claimed to be more strongly linked to auditory sentence processing [1]. However, this modality difference (i.e., the absence of ELANs in many visual studies) is said to be due to specific shortcomings in experimental designs. Thus, Gunter and Friederici [68] suggested that the ELAN in reading studies can *only* be observed under certain “visual input conditions” that involve high contrast and a 300 ms stimulus-presentation time followed by a 200 ms inter-stimulus interval. Importantly, we used these *exact* specifications in our study and still did not observe an ELAN. One could also claim that the absence of ELANs in many reading studies simply indicates that there is no early ERP marker for SCVs, and that the finding of ELAN components in many auditory studies is in reality an artefact due to modality-specific issues, for example prosodic differences between conditions [6]. In order to test if our present findings are indeed modality-specific, we are currently following up with an auditory experiment.

Moreover, our data clearly show that an N400 is present in all conditions containing an SCV, therefore providing an important piece of evidence against semantic blocking, and directly contradicting the “syntax-first” model’s second core claim. Contrary to the standard paradigm used in many previous SCV studies [18,19,22,26,62], in our design, both SC information and lexical-semantic information are available and unambiguous at once [3]. Because the target word is in a complement position, anomalous sentences cannot be “fixed” after the target. Therefore, not only is our design one of the rare ones that employ outright SCVs, but it is also one of the very few ones (possibly the first, as far as we know) to systematically control for true SCVs together with lexical-semantic anomalies. This piece of evidence complements previous findings–such as a large-scale replication failure of the seminal semantic blocking study [24], where members from our lab observed an N400 even in the pure SCV condition (based on almost 200 participants [33]). Because our study implements true SCVs and lexical-semantic anomalies, we were able to observe additive effects of the syntactic and semantic manipulations in the combined condition. Our present finding is important because *unless* semantic blocking is real, N400s elicited by combined anomalies *should* be larger than the N400s observed in response to both pure syntactic and pure semantic errors. Our results are clearly consistent with findings that individuals do not wait until syntactic-category information becomes available to process lexical-semantic information [4,6], contrary to what is predicted by the “syntax-first” approach [1,20,69]. Finally, the priming manipulation used to introduce lexical-semantic anomalies required different word-stems within target pairs. Our SCV condition therefore also reflects lexical-semantic differences: one may argue that there are no *pure* SCVs, since we compare between different word-stems. One possible interpretation of the N400 in response to pure SCVs would therefore be that it mostly reflects lexical-semantic activity. If so, our data would provide yet another piece of evidence against semantic blocking. This also highlights a possible limitation of our design: our data do not allow us to tease apart “pure” SCVs and lexical (word-stem) effects. We will keep this limitation in mind when interpreting ERP responses to syntactic and semantic manipulations.

### 4.2. A revised time-course for the processing of local structures

#### N400 effects

We observed additive N400 effects (and no LAN effect) in response to SCVs and lexical-semantic anomalies, suggesting that the cognitive processes underlying these two linguistic dimensions rely on distinct neural generators. In the introduction, we reviewed arguments that the LAN and N400 are two extremes on a continuum reflecting similar processes, with a topography that can be modulated depending on the input type [41]. In agreement studies, it has been suggested that N400s index a mismatch between word forms (e.g., *is* / *are agreement mismatches on irregular verbs which are expressed as stems [40,71]) while the LAN reflects mismatching transparent affixes [41]. Transposing this to syntactic categories, (left-lateralized) anterior negativities may be observed when the cues to syntactic category are more grammatical in nature (i.e., provided by inflectional or derivational morphemes), while word stem cues, as we have in our experiment, could lead to more central negativities. For example, Hagoort and collaborators observed an anterior negativity in response to syntactic category violations that were cued by a morphophonological marker for a past participle in Dutch (*schroef* ‘propeller’ vs. *schroeft ‘propelled’ [21]), while an N400 was observed in Mandarin Chinese [30–32], a language where syntactic categories are specified by the word-stem. The present design, however, uses inflectional markers for verbs (i.e., infinitive verbs end with–*er*,–*ir*, or–*re*), which should have promoted the elicitation of a LAN instead of an N400. However, contrary to Hagoort and colleagues’ experiment, the morphological markers in our study are ambiguous between derivation and inflection (e.g., *tabler* ‘to table’ can be derived from the noun *table* ‘table’). It is therefore possible that the N400 effect in our experiment reflects the use of derivational morphology to facilitate SC identification. This would be in line with other studies that have reported that mismatches in derivational cues elicit N400 effects rather than LANs (e.g., [72,73]). Since our experiment was not designed to address that question, this interpretation remains speculative.

Globally we observe an N400 in response to both lexical-semantic anomalies and SCVs, and a larger N400 elicited by the combined condition. First, our finding that N400s were found for all types of anomalies clearly contradicts the semantic blocking hypothesis stipulating that semantic processing disappears once a syntactic error is identified, coherent in part with Nickels [33] and Zhang and collaborators [31]. However, Nickels found larger semantic effects in her purely lexical-semantic anomaly condition compared to her combined condition, which is not a definitive argument against blocking. Our results do show additive effects on combined conditions, indicating that lexical-semantic processing persists even in the presence of an SCV.

Assuming that additivity of N400 effects in syntactic and semantic conditions is an indicator that these processes operate in parallel, what could these processes reflect? We suggested that the N400 for SCVs could index the fact that the target word carries the wrong derivational information for the expected word-category (e.g., noun vs. verb). An alternative explanation is that the N400 reflects a mismatch between the expected stem and the actual target. On the other hand, in the lexical-semantic anomaly condition, the N400 likely reflects a facilitation (i.e., an N400 amplitude reduction) in the *correct* condition, because the target is primed. Exploratory analyses revealed that cloze probability modulated N400 effects on semantically-primed sentences regardless of whether their syntax was correct or incorrect, and that this effect was found to show a benefit for *correct* rather than a cost on *incorrect* sentence processing. Nevertheless, we must remain cautious in our interpretation as to whether these processes operate independently. Given that the N400 effects observed are moderate (in the range of 1 μV per process, such that the combined N400-effect of both sub-processes does not go beyond 2.5 μVs), it is still possible that there were enough resources available to process both types of error at the same time using the same neurocognitive resources, and thus the effects would only appear to be additive. Intriguingly, this interpretation is conceivable since the N400 scalp distribution is the same for all conditions. A follow-up study with more salient anomalies may help disambiguate this issue.

#### Early frontal positivity

We observed frontal positivities in response to SCVs in the absence of lexical-semantic anomalies. Similar frontal positivities before or around 600 ms have previously been observed for pure SCVs using a balanced design in English (*He made the* *meal* *to enjoy* vs. He made the *enjoy *the meal* / *He hoped to* *enjoy* *the meal* vs. He hoped to *meal *the enjoy*, unpublished data from Karsten Steinhauer’s Neurocognition of language lab: see S1 Fig). They have also been reported in response to morphosyntactic violations such as subject-verb agreement errors [74]. Some have interpreted this effect as a syntax-specific early P600, reflecting difficulty integrating a constituent within the sentence context [13], or as being related to ambiguity resolution and discourse level complexity [75]. These interpretations do not fit well with our own data, as the SCVs are unambiguous and not more difficult to integrate than combined violations, which don’t elicit a frontal positivity. Alternatively, others have interpreted this effect as a more domain-general P3a [76] normally reflecting surprisal and reallocation of attention [77]. Kasparian et al. [76] observed the frontal positivity only for the first (and arguably less predicted and more salient) morphosyntactic anomaly in a given sentence, thus suggesting a link to surprisal. In our present study, such an effect could be explained by our very constraining syntactic context that makes SCVs very salient (as opposed to previous SCV studies, as we argue in §4.1). The domain-general interpretation, however, does not account for the fact that these frontal positivities were shown to be insensitive to semantic manipulations [43,78]–including in our own paradigm. Given that only a quarter of our sentences contain “pure” SCVs, these violations may be more surprising in the context or our experiment and thus elicit a P3a. This tentative interpretation would need to be tested by future studies.

#### Late P600 effect

Our findings that SCVs elicit a significant P600, in the presence or absence of lexical-semantic anomalies are apparently in line with the widely-accepted view that the P600 reflects syntactic processing and reanalysis [1,14,79]. Further, we observe a main effect of semantics and an interaction between lexical-semantic anomalies and SCVs, revealing that the effects for the two types of errors were not additive. That is, the pure SCV and the combined conditions were statistically indistinguishable. This strongly suggests that the P600s we observe–for both lexical-semantic anomalies and SCVs– both rely on the same neural generators, and therefore compete for the same resources. Since the large syntactic P600 uses up these resources, no additional amplitude increase is seen in the combined condition. This implies that the weak P600 in the semantic condition is qualitatively the same as the one in the syntactic condition, which is incompatible with an interpretation of the P600, even in the pure SCV condition, as being *only* a reflection of structural reanalysis [1,14,79]. It is however completely in line with both the “Monitoring hypothesis” [80] and the interpretation of the P600 as task-related component reflecting well-formedness judgements [81]. We used an acceptability judgement task which encouraged participants to consider both the grammaticality of introductory and experimental sentences and the coherence between them. Finally, the P600 mirrors behavioral responses: lexical-semantic anomalies alone were judged to be worse than correct ones, but all ungrammatical sentences were rated worse than “pure” lexical-semantic ones. These effects may simply reflect the fact that ungrammatical sentences are easier to uniformly categorize as bad compared to semantically anomalous sentences. Since the latter condition is essentially a priming manipulation, not all sentences might have been judged equally unacceptable, thus introducing more variability in categorization.

### 4.3. Between-individual strategies and specific responses to experimental manipulations

Recent work by Tanner and colleagues suggests that individuals rarely display a biphasic negativity-P600 profile, but rather show dominance towards either an N400 or a P600 response. We used correlations to explore inter-individual data and address this possibility, as well as test whether these profiles are consistent across different experimental manipulations. First, we did not observe clear ERP response-dominance patterns in our individuals, except within the lexical-semantic condition where N400 and P600 effects were moderately and negatively correlated (*r =* -.54). This finding is inconsistent with previous observations in agreement [39] and syntactic category violation studies [33]. The main difference between our analyses and the aforementioned studies is that we minimized component overlap or autocorrelation by focusing on distinct electrode sites, and selected time-windows that were further apart than in previous analyses (300 ms vs adjacent time-windows [39] or 50 ms apart [33]) to estimate N400 and P600 effects. Our own data suggest that response dominance should be interpreted with caution. In fact, despite variability, most of our participants displayed a biphasic response to anomalous sentences involving an SCV: that is, our participants generally seemed to engage both mechanisms eliciting N400s and P600s to process SCVs (see [42] for a similar pattern involving LANs and P600s). In the lexical-semantic condition, the N400 and P600 effects were correlated, and while 27 participants elicited either only a N400 or a biphasic response, nine participants did not elicit an N400 at all: it seems that these participants directly engaged context-integration processes, perhaps without benefiting from priming in the correct condition.

The magnitude of the P600 effect is highly correlated between all of our conditions, even the lexical-semantic anomalies and the SCVs, which are different manipulations. Remember that our P600 effects reflected our acceptability ratings: it is therefore not surprising that individuals who are good at categorizing sentences as (un)acceptable can perform this task across the board, and that the cognitive processes underlying this ability are at least partly reflected by the P600. However, while the N400 effects (moderately) correlate between the combined condition and each of the two other ones, they do not correlate between lexical-semantics and SC conditions. Considering that we observed additive processes between Syntax and Semantics on the N400 effect, it could be that different individuals simply recruit different cognitive resources to process these two different types of anomalies. Once again, considering that the correlation coefficients did not actually differ between comparisons, one may want to evaluate this question by implementing stronger violations that would recruit more resources and provide a better opportunity to examine the question of whether or not SC and lexical-semantic processing require shared resources.

## 5. Conclusions and future directions

The present study systematically manipulated syntactic category violations and lexical-semantic anomalies, and showed that syntactic categories are not identified first and do not condition lexical-semantic integration, providing strong evidence against strictly serial models of sentence processing. Rather than observing a LAN-P600 complex (or eLAN-P600, or only a P600) for syntactic category violations, we systematically observed an N400-P600 one. As discussed above, this could be due to the properties of the targets used (i.e., uninflected word-stems). Our data supports proposals where local syntactic and semantic relations are processed simultaneously but independently. Furthermore, the N400-P3a complex elicited by syntactic category violations could reflect a prediction error response: experimental paradigms specifically addressing the additivity of the N400 and exploring the effects on the frontal P3a may shed light on this issue. Following this, the late posterior P600 suggests that syntactic and semantic information is integrated together in discourse-related mechanisms that might have been encouraged by our implementation of an acceptability judgement task. Inspection of the individual data showed that it is unlikely that participants relied on one single sentence-processing mechanism, reflected by either the N400 or the P600 effect alone. Finally, our study showed that while N400 effects elicited by participants were not correlated across all experimental manipulations, individuals who elicited a P600 tended to do so in every condition. We suggest that this component reflects participants’ ability to categorize between correct and unacceptable sentences: ongoing research from the same authors investigating online proficiency effects on ERP responses in native and second language speakers [70] will further address this issue.

## Supporting information

S1 Appendix List of sentences. (DOCX)

S2 Appendix Statistical analyses. (PDF)

S1 Fig Voltage maps comparing SCV effects in native English speakers. These maps illustrate the syntactic category violation effects (violation minus correct) in 100 ms time windows between 300 ms and 1300 ms post onset of the underlined critical words (from left to right).
(DOCX)

S1 Table Paired t-test results comparing control verbs and transitive verbs. (DOCX)

S2 Table Paired t-test results comparing target verbs and nouns. (DOCX)

S3 Table Priming and context effects on target predictability. (DOCX)

S1 Materials https://osf.io/k7vxp/. (DOCX)
