# Supplementary material for: Verbing nouns and nouning verbs: Using a balanced design provides ERP evidence against “syntax-first” approaches to sentence processing
Source: PLoS One. 2020 Mar 13;15(3):e0229169. doi: 10.1371/journal.pone.0229169 (PMC7069651; doi:10.1371/journal.pone.0229169)
Supplement: S1 Appendix — (DOCX) [file pone.0229169.s001.docx]

# S1 Appendix – List of sentences

List of French correct stimuli sentences.

*1 Marie et Jeanne jouent au hockey avec leur copain. Elles osent le plaquer sur le côté.*

*Marie et Jeanne vont au marais avec leur copain. Elles ôtent le crapaud sur le côté.*

*2 Les filles ne doivent pas peinturer le mur. Elles osent le tapisser dans le salon.*

*Les filles doivent nettoyer le sol après la fête. Elles ôtent le confetti dans le salon.*

*3 Les gardiennes ont vu Jérémy passer la barrière. Elles osent le chicaner sans le maître.*

*Les assistantes ont vu le peintre terminer l'œuvre. Elles ôtent le chevalet sans le maître.*

*4 Émilie a réalisé des films d'épouvante. Elle ose les produire dans la nuit.*

*Émilie a déterré les tombeaux des aztèques. Elle ôte les trésors dans la nuit.*

*5 La petite écolière n'a pas peur des grands. Elle ose les taquiner dans la cachette.*

*La nounou nettoie la table des jumeaux. Elle ôte les biberons dans la cuisine.*

*6 Les jumeaux ont volé une Cadillac rouge. Ils osent la conduire dans la montagne.*

*Les frères ont ouvert la tour informatique. Ils ôtent la mémoire dans la machine.*

*7 Les membres de la tribu blâment la sorcière. Ils osent la bannir pour la vie.*

*Les architectes du parc refont l'esquisse. Ils ôtent la fontaine pour la vue.*

*8 L'artiste reçoit les vedettes de cinéma. Il ose les tatouer dans le salon.*

*Michel refait les fondations de la maison. Il ôte les parois dans le salon.*

*9 Les maçonnes entament un nouveau gîte. Elles sont censées le bâtir sous le pont.*

*Les armées attaquent le vieux château. Elles ont cassé le renfort sous le pont.*

*10 La secrétaire doit bien conserver les dossiers. Elle est censée les stocker sur la table.*

*La fillette doit bien nettoyer les bureaux. Elle a cassé les flacons sur la table.*

*11 Julie veut rajouter les clichés à son album. Elle est censée les coller dans la minute.*

*Julie doit réparer les souliers de son amie. Elle a cassé les talons dans la montée.*

*12 Les témoins ne peuvent pas cacher la vérité. Ils sont censés la révéler sous la pression.*

*Les journalistes ne filment pas l'audience. Ils ont cassé la caméra sous la pression.*

*13 Les journalistes examinent la météo. Ils sont censés la prédire pour le public.*

*Les délinquants nient leur méfait. Ils ont cassé la clôture pour le plaisir.*

*14 Les spectateurs ont pris une place réservée. Ils sont censés la céder pour le programme.*

*Les garçons ont emprunté un jouet fragile. Ils ont cassé la moto pour le plaisir.*

*15 Le jardinier arrose ses tulipes. Il est censé les cueillir sous la pluie.*

*Le serveur cache ses maladresses. Il a cassé les théières sous la pression.*

*16 François aide souvent ses cousines. Il est censé les défendre de la milice.*

*François lance férocement des pierres. Il a cassé les fenêtres de la maison.*

*17 La voleuse a dérobé le tableau. Elle souhaite le vendre dans la rue.*

*La guérisseuse a trouvé l'antidote. Elle soigne le peuple dans la rue.*

*18 La choriste apprivoise les couplets de la chanson. Elle souhaite les chanter dans la soirée.*

*L'infirmière travaille dans les services de pédiatrie. Elle soigne les bébés dans la soirée.*

*19 L'illustratrice a inventé un nouveau personnage. Elle souhaite le dessiner dans le véhicule.*

*L'ambulancière a trouvé le char accidenté. Elle soigne le passager dans le véhicule.*

*20 La demoiselle regarde les biscuits encore chauds. Elle souhaite les croquer dans la minute.*

*La chirurgienne s'occupe des patients en urgence. Elle soigne les traumas dans la minute.*

*21 Le marchand a reçu des pépites d'or. Il souhaite les peser pour le fun.*

*L'enfant joue au docteur avec ses copines. Il soigne les poupées pour le fun.*

*22 Le détective cherche une fin à l'intrigue. Il souhaite la conclure pour les proches.*

*Le docteur aide l'accidentée de la route. Il soigne la victime pour les plâtres.*

*23 L'étudiant en dessin reproduit la Joconde. Il souhaite la peindre sans le modèle.*

*L'étudiant en médecine s'occupe du patient. Il soigne la lèvre sans le médecin.*

*24 Pierre s'occupe de ses copines. Il souhaite les saouler dans la nuit.*

*Pierre s'occupe de ses écuries. Il soigne les juments dans la nuit.*

*25 Les bijoutières travaillent sur un petit loquet. Elles ont failli le perdre sur la table.*

*Les écrivaines travaillent sur un nouveau scénario. Elles ont fini le texte sur la table.*

*26 Les campeuses ont pêché un gros saumon. Elles ont failli le bouffer dans la hâte.*

*Les filles ont créé un chef d'œuvre. Elles ont fini le dessin dans la hâte.*

*27 La policière a poursuivi des suspects. Elle a failli les saisir pour de bon.*

*La cantatrice termine les spectacles. Elle a fini les concerts pour de bon.*

*28 La recrue a allumé les bougies blanches. Elle a failli les souffler dans le noir.*

*La recrue a récité les proverbes scouts. Elle a fini les serments dans le noir.*

*29 Martine et Jules possèdent une Ferrari. Ils ont failli la piloter pour la soirée.*

*Martine et Jules consultent une psychologue. Ils ont fini la thérapie pour la semaine.*

*30 Les amis de Camille ont bu la sangria. Ils ont failli la verser sur la table.*

*Les amis de Camille ont mangé la collation. Ils ont fini la pizza sur la table.*

*31 Les cuisiniers ont une patate pourrie. Ils ont failli la frire dans la sauteuse.*

*Les élèves écrivent la dictée difficile. Ils ont fini la phrase dans la souffrance.*

*32 Le cinéaste a trouvé les séquences ennuyeuses. Il a failli les couper pour la sortie.*

*L'artiste a trouvé des musiques entrainantes. Il a fini les chansons pour la sortie.*

*33 La poissonnière dit vérifier ses homards. Elle prétend les sentir dans la matinée.*

*La gestionnaire dit vérifier ses dossiers. Elle présente les rapports dans la matinée.*

*34 La secrétaire a reçu les impôts de la société. Elle prétend les trier pour le trimestre.*

*La stagiaire dévoile les horaires de la cour. Elle présente les procès pour le trimestre.*

*35 La coureuse a égalé le record. Elle prétend le battre par la suite.*

*L'animatrice a ouvert le festival. Elle présente le film par la suite.*

*36 La chirurgienne demande son scalpel. Elle prétend le vouloir pour la chirurgie.*

*La chirurgienne termine son croquis. Elle présente le visage pour la chirurgie.*

*37 Le testeur découvre les liqueurs. Il prétend les renifler pour le client.*

*Le recteur accueille les cohortes. Il présente les facultés pour le collège.*

*38 L'élu du village proteste contre l'église. Il prétend la démolir pour le public.*

*L'élu de la ville accueille la presse nationale. Il présente la capitale pour le public.*

*39 L'apprenti cuisinier doit essayer des recettes. Il prétend les tester dans la journée.*

*L'agent immobilier doit contenter des clientes. Il présente les villas dans la journée.*

*40 L'avocat ne comprend pas le sens de l'affaire. Il prétend la résoudre dans la journée.*

*L'avocat rédige le plaidoyer de la prisonnière. Il présente la défense dans la journée.*

*41 Les violonistes jouent pour le public. Elles vont le ravir de leur charme.*

*Les mécaniciennes travaillent pour Honda. Elles font le moteur de leur char.*

*42 Les écolières voient un chat en danger. Elles vont le secourir dans la hâte.*

*Les écolières crèvent les pneus du char. Elles font le sabotage dans la hâte.*

*43 La danseuse envoûte toujours le spectateur. Elle va le séduire pour le plaisir.*

*La graphiste contente toujours le client. Elle fait le tatouage pour le plaisir.*

*44 Justine a ramassé des gros citrons. Elle va les presser pour le repas.*

*Justine a travaillé sur ses dossiers. Elle fait les projets pour le rapport.*

*45 Les étudiants veulent écouter la partie de hockey. Ils vont la capter dans le local.*

*Les étudiants veulent faire partie de l'association. Ils font la corvée dans le local.*

*46 Joël et Pierre achètent une dinde de Noël. Ils vont la cuisiner dans le four.*

*Joël et Pierre travaillent l'adaptation du rôle. Ils font la comédie dans le film.*

*47 Le chanteur présente ses dernières tounes. Il va les ruiner pour la foule.*

*Le mage présente ses dernières recettes. Il fait les potions pour la foule.*

*48 Martin a quarante ans aujourd'hui. Il va les célébrer pour le fun.*

*Martin dessine les sagas Star Wars aujourd'hui. Il fait les galaxies pour le fun.*

*49 Les pilleuses veulent protéger le trésor. Elles croient le cacher dans le sable.*

*Les pilleuses veulent trouver le trésor. Elles creusent le tombeau dans le sable.*

*50 Lise fait des mauvais rêves avec des zombies. Elle croit les tuer pour la foule.*

*Lise fait des petits fossés pour évacuer l'eau. Elle creuse les sillons pour la fuite.*

*51 L'écolière attrape les bonbons Haribo. Elle croit les voler pour la fille.*

*L'ouvrière aide les villages inondés. Elle creuse les canaux pour la foule.*

*52 La fillette marche sur les tapis. Elle croit les salir de ses pieds.*

*La pelleteuse commence les travaux. Elle creuse les bunkers de sa pelle.*

*53 Les garçons volent la bande-dessinée. Ils croient la rendre dans la journée.*

*Les croque-morts préparent les funérailles. Ils creusent la fosse dans la journée.*

*54 Les voyageurs ont gouté une soupe infecte. Ils croient la vomir dans le jardin.*

*Les ouvriers ont commencé l'installation. Ils creusent la piscine dans le jardin.*

*55 Les soldats s'exercent avec la cible. Ils croient la braquer pour le plaisir.*

*Les soldats gardent leur position. Ils creusent la tranchée pour le passage.*

*56 Paul raconte des histoires à ses enfants. Il croit les dire pour la postérité.*

*Paul accomplit plusieurs tâches au cimetière. Il creuse les tombes pour la postérité.*

*57 La fermière voit le chat affamé. Elle daigne le nourrir dans la cour.*

*La benjamine va au centre équestre. Elle peigne le nourrir dans la cour.*

*58 La patronne se débarrasse de son employé. Elle daigne le muter dans la firme.*

*La fermière a trouvé un petit animal. Elle peigne le muter dans la ferme.*

*59 La modiste n'aime pas ses souliers mauves. Elle daigne les porter dans la maison.*

*La mère n'aime pas les cheveux sales. Elle peigne les porter dans la maison.*

*60 La fille gâtée n'aime pas ses cadeaux. Elle daigne les jeter dans le secret.*

*La coiffeuse termine un de ses clients. Elle peigne les jeter dans le silence.*

*61 Le professeur lit une poésie de Préfontaine. Il daigne la traduire pour le public.*

*Le vétérinaire inspecte l’hyène du zoo. Il peigne la traduire pour le public.*

*62 Le partisan contredit la version officielle. Il daigne la démentir pour le public.*

*Le collectionneur a trouvé une poupée rare. Il peigne la démentir pour le public.*

*63 Le garçon n'aime pas les pastilles roses. Il daigne les traîner dans son sac.*

*Le berger n'aime pas les bêtes sales. Il peigne les troupeaux dans son champ.*

*64 Le papa prend soin des chaises de jardin. Il daigne les rentrer dans la cabane.*

*Le papa prend soin des poupées de sa fille. Il peigne les Barbies® dans la cabane.*

*65 Justine mise sur ses pools de hockey. Elle déclare les parier dans le secret.*

*Justine reçoit des mots de la mafia. Elle déchire les papiers dans le secret.*

*66 L'éditrice a terminé sa commande de manuels. Elle déclare les livrer dans la nuit.*

*La mécène a reçu des faux sans valeur. Elle déchire les tableaux dans la nuit.*

*67 La sorcière s'occupe de son patient. Elle déclare le saigner dans le salon.*

*La sœur veut jouer un mauvais tour. Elle déchire le rideau dans le salon.*

*68 La sorcière libère la princesse de son charme. Elle déclare le rompre dans la caverne.*

*La sorcière rate le sort de mutation. Elle déchire le livre dans la colère.*

*69 Tom est tanné d'avoir mal aux jambes. Il déclare les lever sur le lit.*

*Tom est tanné de se rappeler de son ex. Il déchire les photos sur le lit.*

*70 Simon édite les sketchs de son ami comédien. Il déclare les lire dans le train.*

*Simon déteste la forme de ses essais. Il déchire les pages dans le train.*

*71 Le jury écoute la déposition de la victime. Il déclare la croire pour la sécurité.*

*L'étudiant anticipe l'accusation de plagiat. Il déchire la preuve pour la sécurité.*

*72 Le papa raconte la blague. Il déclare la conter pour le plaisir.*

*Le cuisinier prépare une entrée. Il déchire la laitue pour le plat.*

*73 Les infirmières soignent le genou foulé. Elles doivent le fléchir dans la douleur.*

*Les infirmières achètent un café brûlant. Elles boivent le breuvage dans la douleur.*

*74 Les méchantes chipies veulent rire de Léo. Elles doivent le railler dans la cantine.*

*Les jeunes filles veulent guérir de leur rhume. Elles boivent le bouillon dans la cuisine.*

*75 Léa sort les biscuits brûlants du four. Elle doit les poser sur la table.*

*Léa teste les torréfacteurs de la ville. Elle boit les cafés sur la table.*

*76 La cuisinière a reçu les grains de café. Elle doit les moudre dans la cuisine.*

*La cuisinière reçoit les colis des vergers. Elle boit les cidres dans la cuisine.*

*77 Les chefs préparent la dinde de Noël. Ils doivent la rôtir dans la cuisine.*

*Les chefs se préparent une infusion. Ils boivent la tisane dans la cuisine.*

*78 Les infirmiers s'occupent d'une patiente en état de choc. Ils doivent la consoler dans le calme.*

*Les infirmiers prennent une pause au café du coin. Ils boivent la limonade dans le calme.*

*79 Le joueur voit des formations adverses. Il doit les défier pour le jeu.*

*L'enfant a cru voir des potions magiques. Il boit les lotions pour le jeu.*

*80 Pierre découvre des guêpes agressives. Il doit les fuir dans le village.*

*Pierre visite les brasseries locales. Il boit les bières dans le village.*

*81 Les grandes sœurs ont rencontré leur petit frérot. Elles espèrent le choyer dans la soirée.*

*Les étudiantes ont présenté le théâtre Shakespearien. Elles épatent le doyen dans la soirée.*

*82 Les soldates ont attrapé un nouvel otage. Elles espèrent le retenir dans le combat.*

*Les soldates ont développé un nouveau plan. Elles épatent le général dans le combat.*

*83 La gardienne console les petits enfants. Elle espère les calmer pour la soirée.*

*La stagiaire présente les nouveaux projets. Elle épate les patrons pour la soirée.*

*84 La journaliste a filmé les sabotages. Elle espère les diffuser sur la chaîne.*

*L'étudiante a présenté les manifestes. Elle épate les députés sur la chaîne.*

*85 Les voisins formulent une plainte. Ils espèrent la déposer pour la cause.*

*Les syndiqués manifestent sans trêve. Ils épatent la société pour la cause.*

*86 Les jardiniers repèrent une chienne endormie. Ils espèrent la caresser sur la patte.*

*Les saltimbanques font une acrobatie survoltée. Ils épatent la galerie sur la piste.*

*87 Le grand-père a vécu plusieurs aventures. Il espère les décrire dans la classe.*

*L'enfant a trouvé les dictées très faciles. Il épate les tutrices dans la classe.*

*88 L'animateur présente les activités. Il espère les débuter dans la joie.*

*L'animateur divertit les prisonnières. Il épate les détenues dans la joie.*

*89 Les couturières ont raté le veston. Elles jurent le découdre pour le col.*

*Les chanteuses ont détesté le nouvel opéra. Elles jugent le choriste pour le chœur.*

*90 Les techniciennes ont évalué l'aqueduc. Elles jurent le purifier pour la pluie.*

*Les productrices ont critiqué le vaudeville. Elles jugent le comédien pour la pièce.*

*91 La secrétaire s'occupe des papiers. Elle jure les trouer pour le classeur.*

*La procureure lit les chefs d'accusation. Elle juge les truands pour le conseil.*

*92 La maman s'occupe des dossards humides. Elle jure les sécher sur le porche.*

*La maman voit des braquages à la télé. Elle juge les bandits sur le poste.*

*93 Les parents ferment la fenêtre pendant l'orage. Ils jurent la rouvrir dans la nuit.*

*Les tribunaux corrigent l'affaire de corruption. Ils jugent la décision dans la cour.*

*94 Les élèves ont manqué des questions obligatoires. Ils jurent les refaire dans la soirée.*

*Les cinéastes critiquent une scène d'action. Ils jugent la doublure dans la nuance.*

*95 L'écolier s'occupe des épreuves de biologie. Il jure les terminer dans la classe.*

*Julien a examiné les poursuites judiciaires. Il juge les voleuses dans la soirée.*

*96 Le chevalier voit les armes au sol. Il jure les brandir dans la terreur.*

*Le juré voit entrer les meurtrières. Il juge les crapules dans la terreur.*

*97 Les enseignantes ont reçu un nouveau stagiaire. Elles partent le former pour le travail.*

*Les enseignantes ont appris leur vocabulaire. Elles parlent le jargon pour le travail.*

*98 Les victimes dénoncent le mafieux. Elles partent le trahir dans la terreur.*

*Les cousines se rendent à Hawaï. Elles parlent le créole dans la taverne.*

*99 Les ouvrières montent un meuble. Elles partent le sabler dans la cour.*

*Les adolescentes s'échangent un secret. Elles parlent le verlan dans la cour.*

*100 Les savantes ont un mélange trop concentré. Elles partent le diluer dans le labo.*

*Les infirmières soignent un patient étranger. Elles parlent le patois dans le labo.*

*101 Les armées voient un trou dans la muraille. Elles partent le bloquer dans la soirée.*

*Les joueuses de tennis rencontrent le journaliste. Elles parlent le français dans la soirée.*

*102 Les chirurgiennes ont reçu le nouveau foie. Elles partent le greffer pour le patient.*

*Les enseignantes ont voyagé en territoire belge. Elles parlent le flamand pour le plaisir.*

*103 Les étudiantes se réunissent pour leur rapport. Elles partent le rédiger pour le cours.*

*Les étudiantes ont appris un nouvel idiome. Elles parlent le polonais pour le cours.*

*104 Les sœurs ont reçu leur chiot. Elles partent le cajoler par la suite.*

*Les sœurs ont participé à un séjour linguistique. Elles parlent le mandarin par la suite.*

*105 La maman ours a besoin de saumons. Elle est supposée les pêcher pour les petits.*

*La cuisinière a terminé ses desserts. Elle a savouré les soufflés pour les petits.*

*106 La cuisinière prépare du pain. Elle est supposée le beurrer par la suite.*

*La cuisinière termine un dessert. Elle a savouré le nougat par la suite.*

*107 La campeuse poursuit le lapin. Elle est supposée le chasser dans la campagne.*

*La voisine concocte un dessert. Elle a savouré le gâteau dans la cuisine.*

*108 Les concurrents vont saboter la toile gagnante. Ils sont supposés la gâcher dans le silence.*

*Les étudiants admirent leur professeur. Ils ont savouré la leçon dans le silence.*

*109 Les athlètes s'entraînent peu pour la médaille. Ils sont supposés la mériter pour le principe.*

*Les évadés sont capturés après leur fuite. Ils ont savouré la liberté pour le principe.*

*110 Les gardiens ont retrouvé la preuve. Ils sont supposés la détruire dans le secret.*

*Les garçons ont gagné la partie. Ils ont savouré la victoire dans le secret.*

*111 Le couturier a reçu beaucoup de vestes. Il est supposé les coudre sur la table.*

*Le cuisinier a goûté beaucoup de pâtisseries. Il a savouré les tartes sur la table.*

*112 Tom s'occupe des pelouses de ses voisines. Il est supposé les tondre vers le dîner.*

*Tom se goinfre des sucreries de ses sœurs. Il a savouré les gaufres vers le dîner.*

*113 Les dresseuses trouvent un husky dans la nourriture. Elles pensent le punir dans la cuisine.*

*Les filles commencent un sport dans la nature. Elles poussent le traineau dans la colline.*

*114 Les étudiantes voient un camarade de classe. Elles pensent le saluer dans la rue.*

*Les étudiantes aident le déménageur en panne. Elles poussent le camion dans la rue.*

*115 La grand-mère attend les visiteurs. Elle pense les guetter dans la rue.*

*La livreuse déplace les paquetages. Elle pousse les colis dans la rue.*

*116 La chef a reçu ses langoustines. Elle pense les cuire dans la cuisine.*

*La chef a reçu ses cartons. Elle pousse les boîtes dans la cuisine.*

*117 La modéliste achète des escarpins dispendieux. Elle pense les choisir dans la réserve.*

*La mécanicienne reçoit des autos en panne. Elle pousse les voitures dans la ruelle.*

*118 Les locataires ont fini leur lessive. Ils pensent la pendre dans la garde-robe.*

*Les locataires organisent leur cuisine. Ils poussent la table dans le garde-manger.*

*119 Les stylistes doivent changer la couleur de la mèche. Ils pensent la pâlir sans le patron.*

*Les cochers doivent sortir la diligence embourbée. Ils poussent la calèche sans le patron.*

*120 L'inspecteur voit un lien entre les agressions. Il pense les relier sans le patron.*

*Le marchand fait le ménage des rayons. Il pousse les chariots sans le patron.*

*121 Les cuisinières ont fini le potage. Elles peuvent le chauffer dans la cuisine.*

*Les décoratrices s'occupent du salon. Elles posent le tapis dans la cuisine.*

*122 Les jumelles ont réparé leur vélo de route. Elles peuvent le pédaler sur le bitume.*

*Les jumelles ont ramené un souvenir de voyage. Elles posent le bibelot sur le buffet.*

*123 La jeune escrimeuse voit ses adversaires. Elle peut les vaincre pour le plaisir.*

*La décoratrice ajoute les détails décoratifs. Elle pose les cadres pour le plaisir.*

*124 La jeune étudiante mange des grosses portions. Elle peut les réduire sans mon aide.*

*La propriétaire redécore ses salles de bains. Elle pose les miroirs sans mon aide.*

*125 Les campeurs boivent l'eau du lac. Ils peuvent la filtrer pour la sécurité.*

*Les jurés commencent leur évaluation. Ils posent la question pour la sécurité.*

*126 Les pâtissiers préparent la brioche. Ils peuvent la pétrir sur la table.*

*Les chasseurs reviennent de la chasse. Ils posent la carcasse sur la table.*

*127 Le toiletteur lave mes chiennes. Il peut les coiffer sans mon aide.*

*Le nettoyeur prend mes tuniques. Il pose les tenues sans mon aide.*

*128 L'électricien a installé toutes ses prises. Il peut les brancher pour le chauffage.*

*Le jardinier termine la cour arrière. Il pose les statues pour le chemin.*

*129 Les organisatrices changent le nombre d'invités. Elles semblent le tripler sans mon aide.*

*Les partisanes de la révolution préparent un coup. Elles ciblent le clergé sans mon aide.*

*130 Les caissières ont un nouveau système. Elles semblent le parfaire pour la vente.*

*Les hôtesses préparent l'argument commercial. Elles ciblent le public pour la vente.*

*131 La vieille dame dirige les partis. Elle semble les fonder pour le plaisir.*

*La vieille dame nourrit les oiseaux. Elle cible les pigeons pour le plaisir.*

*132 La mécanicienne a inspecté les capots. Elle semble les rabattre pour les moteurs.*

*L'entraîneuse a présenté les exercices. Elle cible les biceps pour les muscles.*

*133 Les inspecteurs ont résolu l'affaire complexe. Ils semblent la clore pour la famille.*

*Les médicaments combattent la maladie. Ils ciblent la grippe pour la famille.*

*134 Les hommes prient devant la nouvelle sainte. Ils semblent la bénir pour de bon.*

*Les comprimés combattent la blessure musculaire. Ils ciblent la douleur pour de bon.*

*135 Le paparazzi dépose ses deux valises à l'hôtel. Il semble les défaire pour la soirée.*

*Le paparazzi arrive sur les collines d'Hollywood. Il cible les vedettes pour la soirée.*

*136 Le voyageur ouvre toutes les victuailles. Il semble les dévorer dans le silence.*

*Le chiffon nettoie toutes les surfaces. Il cible les saletés dans le salon.*

*137 Les pâtissières utilisent du beurre trop dur. Elles songent le ramollir dans la cuisine.*

*Les pâtissières cherchent le réfrigérateur. Elles longent le corridor dans la cuisine.*

*138 Les adolescentes font le dessin d'un beau cardinal. Elles songent le rougir sur le bord.*

*Les adolescentes cherchent le cellulaire tombé du pont. Elles longent le canal sur le bord.*

*139 Jessica doit résoudre ses problèmes de math. Elle songe les déduire pour la réponse.*

*Jessica ne trouve pas les bureaux administratifs. Elle longe les couloirs pour la recherche.*

*140 L'escrimeuse a acheté des sabres. Elle songe les manier dans le secret.*

*La campeuse est égarée dans les bois. Elle longe les sentiers dans le silence.*

*141 Les touristes approchent une vache sauvage. Ils songent la traire pour le plaisir.*

*Les touristes marchent au bord de la mer. Ils longent la plage pour le plaisir.*

*142 Les designers ont créé une médaille d'or. Ils songent la jaunir dans le silence.*

*Les accros du trekking sont rendus en Chine. Ils longent la muraille dans le silence.*

*143 Le campeur prépare les tasses de café. Il songe les remplir dans le silence.*

*Le campeur recherche la meilleure cachette. Il longe les rivières dans le silence.*

*144 Mario possède de vieilles chaises. Il songe les polir dans le jardin.*

*Mario chasse les nombreuses taupes. Il longe les cabanes dans le jardin.*

*145 Les filles ont du pain au congélateur. Elles viennent le dégeler pour le repas.*

*Les filles font un achat au supermarché. Elles tiennent le salami pour le repas.*

*146 Les protestantes s'en prennent au vitrail de l'église. Elles viennent le casser pour le principe.*

*Les belles-sœurs amènent un présent. Elles tiennent le cadeau pour le petit.*

*147 La gardienne porte secours aux enfants qui se noient. Elle vient les sauver par la main.*

*La gardienne porte secours aux enfants perdus. Elle tient les petits par la main.*

*148 La policière voit des voyous suspicieux. Elle vient les fouiller dans la poche.*

*La festivalière vient voir des groupes connus. Elle tient les billets dans la poche.*

*149 Les ouvriers n'aiment pas la nouvelle assurance. Ils viennent la négocier pour le principe.*

*Les danseurs n'aiment pas faire une arabesque. Ils tiennent la position pour le principe.*

*150 Les athlètes s'entrainent sur la piste de 500m. Ils viennent la courir pour le plaisir.*

*Les amoureux investissent dans une bonbonnerie. Ils tiennent la boutique pour le plaisir.*

*151 Louis prépare plusieurs semences. Il vient les répandre sur la terre.*

*Louis a besoin d'aides visuelles. Il tient les lunettes sur la tête.*

*152 Le docteur a reçu deux patientes. Il vient les guérir pour les parents.*

*Le mari attend les cartes d'embarquement. Il tient les valises pour les parents.*

*153 Sophie connaît le ballet et le hip-hop. Elle affirme les danser dans la classe.*

*Sophie note bien les garçons et les filles. Elle informe les parents dans la classe.*

*154 La critique culinaire trouve les brocolis fades. Elle affirme les saler pour le goût.*

*La recrue scoute voit des animaux sauvages. Elle informe les cadets pour le groupe.*

*155 La frimeuse montre les albums de dessin. Elle affirme les colorier de son talent.*

*La frimeuse vole les chèques des apprentis. Elle informe les salariés de son talent.*

*156 L'éditrice n'aime pas ces romans. Elle affirme les relire pour la critique.*

*La lectrice n'aime pas ces romans. Elle informe les vendeurs pour la critique.*

*157 Le vieillard adore ses petites-filles. Il affirme les chérir dans la tristesse.*

*Le vieillard voit des tornades à l'horizon. Il informe les gamines dans la tourmente.*

*158 L'escroc pirate des cartes électroniques. Il affirme les saboter dans la journée.*

*Le soldat transmet le message du président. Il informe les colonies dans la journée.*

*159 Le garçon finit les courtes évaluations. Il affirme les passer par lui-même.*

*Le garçon voit ses copines s'évanouir. Il informe les mamans par lui-même.*

*160 Le stagiaire doit terminer la facture. Il affirme la finir par lui-même.*

*Le stagiaire doit terminer l'enquête. Il affirme la politiser dans la soirée.*
