# Supplementary material for: Verbing nouns and nouning verbs: Using a balanced design provides ERP evidence against “syntax-first” approaches to sentence processing
Source: PLoS One. 2020 Mar 13;15(3):e0229169. doi: 10.1371/journal.pone.0229169 (PMC7069651; doi:10.1371/journal.pone.0229169)
Supplement: S2 Appendix — (PDF) [file pone.0229169.s002.pdf]

Supporting information: Statistical analyses

Lauren A. Fromont

I. Behavioural data: Cumulative link mixed model output

```
## Cumulative Link Mixed Model fitted with the Laplace approximation
##
## formula: RESP ~ SYN * SEM + (Condition | Subj)
## data:    subset(scores_acc, Group == "L1")
##
##  link  threshold nobs  logLik    AIC      niter       max.grad cond.H
##  logit flexible  11481 -14839.38 29712.76 2391(11960) 1.92e-02 8.0e+02
##
## Random effects:
##  Groups Name            Variance Std.Dev. Corr
##  Subj   (Intercept)     0.6289   0.7931
##         ConditionSEM    1.0586   1.0289   -0.359
##         ConditionSYN    2.1502   1.4664   -0.325  0.863
##         ConditionSYNSEM 2.7203   1.6493   -0.302  0.903  0.995
## Number of groups:  Subj 36
##
## Coefficients:
##                   Estimate Std. Error z value Pr(>|z|)
## SYNINCOR            3.0997     0.2511  12.342   <2e-16 ***
## SEMINCOR            1.9711     0.1795  10.979   <2e-16 ***
## SYNINCOR:SEMINCOR  -1.5250     0.1550  -9.838   <2e-16 ***
## ---
## Signif. codes:  0 '***' 0.001 '**' 0.01 '*' 0.05 '.' 0.1 ' ' 1
##
## Threshold coefficients:
##     Estimate Std. Error z value
## 1|2  -0.3013     0.1375  -2.191
## 2|3   1.0301     0.1382   7.453
## 3|4   2.2007     0.1395  15.775
## 4|5   3.7797     0.1419  26.636
```

```
## Cumulative Link Mixed Model fitted with the Laplace approximation
##
## formula: RESP ~ SYN + SEM + (Condition | Subj)
## data:    subset(scores_acc, Group == "L1")
##
##  link  threshold nobs  logLik    AIC      niter       max.grad cond.H
##  logit flexible  11481 -14863.36 29758.72 2095(11839) 7.11e-03 4.3e+03
##
## Random effects:
##  Groups Name            Variance Std.Dev. Corr
##  Subj   (Intercept)     0.9605   0.9801
##         ConditionSEM    3.8954   1.9737   -0.653
##         ConditionSYN    6.5299   2.5554   -0.632  0.957
##         ConditionSYNSEM 7.6374   2.7636   -0.619  0.966  0.998
## Number of groups:  Subj 36
```

```
##
## Coefficients:
##          Estimate Std. Error z value Pr(>|z|)
## SYNINCOR   1.0190     0.2708   3.763 0.000168 ***
## SEMINCOR   0.2940     0.1204   2.441 0.014629 *
## ---
## Signif. codes:  0 '***' 0.001 '**' 0.01 '*' 0.05 '.' 0.1 ' ' 1
##
## Threshold coefficients:
##     Estimate Std. Error z value
## 1|2  -0.8762     0.2474  -3.541
## 2|3   0.4533     0.2467   1.837
## 3|4   1.6243     0.2475   6.562
## 4|5   3.2066     0.2496  12.848
```

```
## Likelihood ratio tests of cumulative link models:
##
##              formula:                              link: threshold:
## scores_mL1ii RESP ~ SYN + SEM + (Condition | Subj) logit flexible
## scores_mL1i  RESP ~ SYN * SEM + (Condition | Subj) logit flexible
##
##              no.par   AIC logLik LR.stat df Pr(>Chisq)
## scores_mL1ii     16 29759 -14863
## scores_mL1i      17 29713 -14839  47.959  1  4.352e-12 ***
## ---
## Signif. codes:  0 '***' 0.001 '**' 0.01 '*' 0.05 '.' 0.1 ' ' 1
```

II. ERP data: Mixed effects models outputs

A. ELAN: Time window 100 ms - 300 ms

The full models show no effect or interaction involving Syntax (SYN) or Semantics (SEM)

At Midline electrodes

```
## Linear mixed model fit by maximum likelihood . t-tests use
##   Satterthwaite's method [lmerModLmerTest]
## Formula: ELAN ~ SYN * SEM * Ant + (Cond | Subj) + (cCibleP_COR + Cond |
##     Item)
##    Data: FWC_Mid
##
##       AIC       BIC    logLik  deviance  df.resid
##  224572.0  224858.2 -112252.0  224504.0     33426
##
## Scaled residuals:
##     Min      1Q  Median      3Q     Max
## -5.4947 -0.6335 -0.0058  0.6286  4.8604
##
## Random effects:
##  Groups   Name        Variance Std.Dev. Corr
##  Item     (Intercept)  2.6097  1.615
##           cCibleP_COR 10.3157  3.212     0.07
```

```
##           CondSEM      3.9575  1.989    -0.74 -0.25
##           CondSYN      4.9866  2.233    -0.68 -0.13  0.48
##           CondSYNSEM   4.2346  2.058    -0.74  0.04  0.64  0.48
##  Subj     (Intercept)  1.6123  1.270
##           CondSEM      1.4015  1.184     0.03
##           CondSYN      0.8912  0.944    -0.19  0.57
##           CondSYNSEM   1.1301  1.063    -0.17  0.46  0.36
##  Residual             46.4145  6.813
## Number of obs: 33460, groups:  Item, 160; Subj, 36
##
## Fixed effects:
##                             Estimate Std. Error         df t value
## (Intercept)                2.183e+00  2.705e-01  7.170e+01   8.068
## SYNINCOR                  -9.292e-02  2.806e-01  1.154e+02  -0.331
## SEMINCOR                  -1.421e-01  2.941e-01  9.215e+01  -0.483
## AntPost                   -6.326e-01  1.477e-01  3.256e+04  -4.283
## SYNINCOR:SEMINCOR          1.042e-01  4.150e-01  8.236e+01   0.251
## SYNINCOR:AntPost           1.096e-02  2.092e-01  3.256e+04   0.052
## SEMINCOR:AntPost           1.482e-01  2.108e-01  3.256e+04   0.703
## SYNINCOR:SEMINCOR:AntPost  8.829e-03  2.980e-01  3.256e+04   0.030
##                           Pr(>|t|)
## (Intercept)               1.17e-11 ***
## SYNINCOR                     0.741
## SEMINCOR                     0.630
## AntPost                   1.85e-05 ***
## SYNINCOR:SEMINCOR            0.802
## SYNINCOR:AntPost             0.958
## SEMINCOR:AntPost             0.482
## SYNINCOR:SEMINCOR:AntPost    0.976
## ---
## Signif. codes:  0 '***' 0.001 '**' 0.01 '*' 0.05 '.' 0.1 ' ' 1
##
## Correlation of Fixed Effects:
##                     (Intr) SYNINCOR SEMINCOR AntPst SYNINCOR:SEMINCOR
## SYNINCOR            -0.429
## SEMINCOR            -0.307  0.512
## AntPost             -0.273  0.263    0.251
## SYNINCOR:SEMINCOR    0.209 -0.737   -0.693   -0.178
## SYNINCOR:AP          0.193 -0.373   -0.177   -0.706  0.252
## SEMINCOR:AP          0.191 -0.184   -0.358   -0.701  0.254
## SYNINCOR:SEMINCOR: -0.135  0.262    0.254    0.496 -0.359
##                     SYNINCOR:A SEMINCOR:
## SYNINCOR
## SEMINCOR
## AntPost
## SYNINCOR:SEMINCOR
## SYNINCOR:AP
## SEMINCOR:AP          0.495
## SYNINCOR:SEMINCOR: -0.702     -0.707
```

Anova wrapper

```
## Analysis of Deviance Table (Type III Wald chisquare tests)
##
## Response: ELAN
```

```
##               Chisq Df Pr(>Chisq)
## (Intercept) 65.0972  1  7.129e-16 ***
## SYN          0.1096  1     0.7405
## SEM          0.2335  1     0.6289
## Ant         18.3472  1  1.841e-05 ***
## SYN:SEM      0.0630  1     0.8018
## SYN:Ant      0.0027  1     0.9582
## SEM:Ant      0.4940  1     0.4821
## SYN:SEM:Ant  0.0009  1     0.9764
## ---
## Signif. codes:  0 '***' 0.001 '**' 0.01 '*' 0.05 '.' 0.1 ' ' 1
```

R-squared (marginal and conditional)

```
## Warning: 'r.squaredGLMM' now calculates a revised statistic. See the help
## page.
```

```
##              R2m       R2c
## [1,] 0.001526027 0.1000653
```

At Lateral electrodes

```
## Linear mixed model fit by maximum likelihood . t-tests use
##   Satterthwaite's method [lmerModLmerTest]
## Formula: ELAN ~ SYN * SEM * Ant * Hemi + (Cond | Subj) + (cCibleP_COR +
##     Cond | Item)
##    Data: FWC_Lat
##
##       AIC       BIC    logLik  deviance  df.resid
##  755719.0  756125.2 -377817.5  755635.0    117068
##
## Scaled residuals:
##     Min      1Q  Median      3Q     Max
## -6.8719 -0.6293 -0.0149  0.6097  6.1084
##
## Random effects:
##  Groups   Name        Variance Std.Dev. Corr
##  Item     (Intercept)  1.8226  1.3500
##           cCibleP_COR 25.3718  5.0370    0.29
##           CondSEM      3.0275  1.7400   -0.70 -0.17
##           CondSYN      3.4289  1.8517   -0.63 -0.15  0.47
##           CondSYNSEM   2.9497  1.7175   -0.61  0.15  0.61  0.43
##  Subj     (Intercept)  0.7822  0.8844
##           CondSEM      0.8365  0.9146   -0.03
##           CondSYN      0.5877  0.7666   -0.19  0.49
##           CondSYNSEM   0.8668  0.9310   -0.22  0.41  0.35
##  Residual             36.4957  6.0412
## Number of obs: 117110, groups:  Item, 160; Subj, 36
##
## Fixed effects:
##                                      Estimate Std. Error         df
## (Intercept)                         1.375e+00  1.926e-01  8.578e+01
## SYNINCOR                            1.257e-01  2.156e-01  1.484e+02
## SEMINCOR                           -8.385e-02  2.258e-01  1.169e+02
## AntPost                            -5.036e-01  1.000e-01  1.162e+05
```

```
## Hemiright                            -7.499e-02  9.260e-02  1.162e+05
## SYNINCOR:SEMINCOR                    -7.153e-03  3.211e-01  1.051e+02
## SYNINCOR:AntPost                     -6.548e-02  1.417e-01  1.162e+05
## SEMINCOR:AntPost                      5.775e-02  1.428e-01  1.162e+05
## SYNINCOR:Hemiright                   -2.406e-01  1.311e-01  1.162e+05
## SEMINCOR:Hemiright                    9.118e-03  1.322e-01  1.162e+05
## AntPost:Hemiright                     5.284e-01  1.415e-01  1.162e+05
## SYNINCOR:SEMINCOR:AntPost             6.380e-03  2.018e-01  1.162e+05
## SYNINCOR:SEMINCOR:Hemiright           1.640e-01  1.868e-01  1.162e+05
## SYNINCOR:AntPost:Hemiright            6.360e-02  2.003e-01  1.162e+05
## SEMINCOR:AntPost:Hemiright           -6.228e-02  2.019e-01  1.162e+05
## SYNINCOR:SEMINCOR:AntPost:Hemiright  6.904e-02  2.854e-01  1.162e+05
##                                     t value Pr(>|t|)
## (Intercept)                           7.140 2.83e-10 ***
## SYNINCOR                              0.583 0.560653
## SEMINCOR                             -0.371 0.711017
## AntPost                              -5.035 4.79e-07 ***
## Hemiright                            -0.810 0.418067
## SYNINCOR:SEMINCOR                    -0.022 0.982268
## SYNINCOR:AntPost                     -0.462 0.643889
## SEMINCOR:AntPost                      0.405 0.685795
## SYNINCOR:Hemiright                   -1.835 0.066577 .
## SEMINCOR:Hemiright                    0.069 0.944996
## AntPost:Hemiright                     3.736 0.000187 ***
## SYNINCOR:SEMINCOR:AntPost             0.032 0.974780
## SYNINCOR:SEMINCOR:Hemiright           0.878 0.380107
## SYNINCOR:AntPost:Hemiright            0.317 0.750885
## SEMINCOR:AntPost:Hemiright           -0.308 0.757709
## SYNINCOR:SEMINCOR:AntPost:Hemiright   0.242 0.808851
## ---
## Signif. codes:  0 '***' 0.001 '**' 0.01 '*' 0.05 '.' 0.1 ' ' 1
```

```
##
## Correlation matrix not shown by default, as p = 16 > 12.
## Use print(x, correlation=TRUE)  or
##   vcov(x)     if you need it
```

Anova wrapper

```
## Analysis of Deviance Table (Type III Wald chisquare tests)
##
## Response: ELAN
##                   Chisq Df Pr(>Chisq)
## (Intercept)      50.9775  1  9.343e-13 ***
## SYN               0.3401  1  0.5597675
## SEM               0.1379  1  0.7103449
## Ant              25.3510  1  4.779e-07 ***
## Hemi              0.6557  1  0.4180656
## SYN:SEM           0.0005  1  0.9822253
## SYN:Ant           0.2137  1  0.6438884
## SEM:Ant           0.1637  1  0.6857946
## SYN:Hemi          3.3655  1  0.0665744 .
## SEM:Hemi          0.0048  1  0.9449960
## Ant:Hemi         13.9553  1  0.0001872 ***
## SYN:SEM:Ant       0.0010  1  0.9747803
```

```
## SYN:SEM:Hemi      0.7704  1  0.3801048
## SYN:Ant:Hemi      0.1008  1  0.7508843
## SEM:Ant:Hemi      0.0952  1  0.7577081
## SYN:SEM:Ant:Hemi  0.0585  1  0.8088506
## ---
## Signif. codes:  0 '***' 0.001 '**' 0.01 '*' 0.05 '.' 0.1 ' ' 1
```

R-squared (marginal and conditional)

```
##               R2m       R2c
## [1,] 0.0008926826 0.1081204
```

B. N400: Time window 350 ms - 500 ms

The best fits involved no interactions involving the factors Syntax (SYN) and Semantics (SEM)

At Midline electrodes

First, we ran the full model with factors Syntax (SYN), Semantics (SEM), Anteriority (Ant) and their interactions

```
## Linear mixed model fit by maximum likelihood . t-tests use
##   Satterthwaite's method [lmerModLmerTest]
## Formula: N400 ~ SYN * SEM * Ant + (Cond | Subj) + (cCibleP_COR + Cond |
##     Item)
##    Data: FWC_Mid
##
##       AIC       BIC    logLik  deviance  df.resid
##  236590.5  236876.7 -118261.2  236522.5     33426
##
## Scaled residuals:
##     Min      1Q  Median      3Q     Max
## -5.1745 -0.6328 -0.0161  0.6213  4.9724
##
## Random effects:
##  Groups   Name        Variance Std.Dev. Corr
##  Item     (Intercept)  4.6908  2.1658
##           cCibleP_COR 16.2858  4.0356    0.01
##           CondSEM      7.6597  2.7676   -0.77  0.05
##           CondSYN      6.3467  2.5193   -0.74  0.00  0.63
##           CondSYNSEM   6.2276  2.4955   -0.80 -0.03  0.66  0.74
##  Subj     (Intercept)  2.6241  1.6199
##           CondSEM      1.3247  1.1509   -0.18
##           CondSYN      0.5756  0.7587    0.19  0.10
##           CondSYNSEM   1.5722  1.2539   -0.25  0.48  0.75
##  Residual             66.5592  8.1584
## Number of obs: 33460, groups:  Item, 160; Subj, 36
##
## Fixed effects:
##                            Estimate Std. Error         df t value
## (Intercept)               1.582e+00  3.463e-01  7.498e+01   4.567
## SYNINCOR                 -7.129e-01  2.968e-01  1.431e+02  -2.402
## SEMINCOR                 -1.414e+00  3.436e-01  1.273e+02  -4.116
## AntPost                  -1.768e+00  1.769e-01  3.250e+04  -9.997
```

```
## SYNINCOR:SEMINCOR          2.933e-01  3.922e-01  1.439e+02   0.748
## SYNINCOR:AntPost           1.106e-02  2.505e-01  3.250e+04   0.044
## SEMINCOR:AntPost           1.946e-01  2.524e-01  3.250e+04   0.771
## SYNINCOR:SEMINCOR:AntPost  3.173e-02  3.569e-01  3.250e+04   0.089
##                           Pr(>|t|)
## (Intercept)               1.90e-05 ***
## SYNINCOR                    0.0176 *
## SEMINCOR                  6.89e-05 ***
## AntPost                    < 2e-16 ***
## SYNINCOR:SEMINCOR           0.4558
## SYNINCOR:AntPost            0.9648
## SEMINCOR:AntPost            0.4406
## SYNINCOR:SEMINCOR:AntPost   0.9292
## ---
## Signif. codes:  0 '***' 0.001 '**' 0.01 '*' 0.05 '.' 0.1 ' ' 1
##
## Correlation of Fixed Effects:
##                     (Intr) SYNINCOR SEMINCOR AntPst SYNINCOR:SEMINCOR
## SYNINCOR            -0.342
## SEMINCOR            -0.460  0.449
## AntPost             -0.255  0.298    0.257
## SYNINCOR:SEMINCOR    0.238 -0.589   -0.737   -0.225
## SYNINCOR:AP          0.180 -0.422   -0.182   -0.706  0.319
## SEMINCOR:AP          0.179 -0.209   -0.367   -0.701  0.322
## SYNINCOR:SEMINCOR: -0.127  0.296    0.260    0.496 -0.455
##                     SYNINCOR:A SEMINCOR:
## SYNINCOR
## SEMINCOR
## AntPost
## SYNINCOR:SEMINCOR
## SYNINCOR:AP
## SEMINCOR:AP          0.495
## SYNINCOR:SEMINCOR: -0.702     -0.707
```

Then, we ran the same model, and remove the interaction with Anteriority (Ant)

```
## Linear mixed model fit by maximum likelihood . t-tests use
##   Satterthwaite's method [lmerModLmerTest]
## Formula: N400 ~ SYN * SEM + Ant + (Cond | Subj) + (cCibleP_COR + Cond |
##     Item)
##    Data: FWC_Mid
##
##       AIC       BIC    logLik  deviance  df.resid
##  236585.9  236846.9 -118262.0  236523.9     33429
##
## Scaled residuals:
##     Min      1Q  Median      3Q     Max
## -5.1677 -0.6341 -0.0160  0.6200  4.9645
##
## Random effects:
##  Groups   Name        Variance Std.Dev. Corr
##  Item     (Intercept)  4.6907  2.1658
##           cCibleP_COR 16.2846  4.0354    0.01
##           CondSEM      7.6596  2.7676   -0.77  0.05
##           CondSYN      6.3466  2.5192   -0.74  0.00  0.63
```

```
##           CondSYNSEM   6.2275  2.4955   -0.80 -0.03  0.66  0.74
##  Subj     (Intercept)  2.6241  1.6199
##           CondSEM      1.3246  1.1509   -0.18
##           CondSYN      0.5756  0.7587    0.19  0.10
##           CondSYNSEM   1.5722  1.2539   -0.25  0.48  0.75
##  Residual             66.5621  8.1586
## Number of obs: 33460, groups:  Item, 160; Subj, 36
##
## Fixed effects:
##                     Estimate Std. Error         df t value Pr(>|t|)
## (Intercept)           1.5269     0.3377    67.8671   4.521 2.54e-05 ***
## SYNINCOR             -0.7073     0.2691    96.6405  -2.628  0.00998 **
## SEMINCOR             -1.3166     0.3195    95.2692  -4.120 8.07e-05 ***
## AntPost              -1.6588     0.0892 32501.9044 -18.596  < 2e-16 ***
## SYNINCOR:SEMINCOR     0.3091     0.3493    90.4717   0.885  0.37848
## ---
## Signif. codes:  0 '***' 0.001 '**' 0.01 '*' 0.05 '.' 0.1 ' ' 1
##
## Correlation of Fixed Effects:
##             (Intr) SYNINCOR SEMINC AntPst
## SYNINCOR    -0.301
## SEMINCOR    -0.435  0.442
## AntPost     -0.132  0.000    0.000
## SYNINCOR:SE  0.208 -0.562   -0.747  0.000
```

Comparing those models shows that we should be using the second one

```
## Data: FWC_Mid
## Models:
## neg_basic3: N400 ~ SYN * SEM + Ant + (Cond | Subj) + (cCibleP_COR + Cond |
## neg_basic3:     Item)
## neg_basic2: N400 ~ SYN * SEM * Ant + (Cond | Subj) + (cCibleP_COR + Cond |
## neg_basic2:     Item)
##            Df    AIC    BIC  logLik deviance  Chisq Chi Df Pr(>Chisq)
## neg_basic3 31 236586 236847 -118262   236524
## neg_basic2 34 236590 236877 -118261   236522 1.4237      3        0.7
```

Then, we removed the remaining interaction between Syntax (SYN) and Semantics (SEM)

```
## Linear mixed model fit by maximum likelihood . t-tests use
##   Satterthwaite's method [lmerModLmerTest]
## Formula: N400 ~ SYN + SEM + Ant + (Cond | Subj) + (cCibleP_COR + Cond |
##     Item)
##    Data: FWC_Mid
##
##       AIC       BIC    logLik  deviance  df.resid
##  236584.7  236837.2 -118262.3  236524.7     33430
##
## Scaled residuals:
##     Min      1Q  Median      3Q     Max
## -5.1657 -0.6335 -0.0161  0.6195  4.9667
##
## Random effects:
##  Groups   Name        Variance Std.Dev. Corr
##  Item     (Intercept)  4.7060  2.1693
##           cCibleP_COR 16.3394  4.0422    0.01
```

```
##           CondSEM      7.6877  2.7727   -0.77  0.04
##           CondSYN      6.3743  2.5247   -0.75 -0.01  0.63
##           CondSYNSEM   6.2285  2.4957   -0.80 -0.03  0.66  0.74
##  Subj     (Intercept)  2.6253  1.6203
##           CondSEM      1.3324  1.1543   -0.18
##           CondSYN      0.5789  0.7609    0.19  0.10
##           CondSYNSEM   1.5737  1.2545   -0.25  0.48  0.75
##  Residual             66.5615  8.1585
## Number of obs: 33460, groups:  Item, 160; Subj, 36
##
## Fixed effects:
##                Estimate Std. Error         df t value Pr(>|t|)
## (Intercept)      1.4645     0.3305    58.1719   4.431 4.21e-05 ***
## SYNINCOR        -0.5731     0.2227    76.3849  -2.574    0.012 *
## SEMINCOR        -1.1054     0.2124    69.1811  -5.204 1.90e-06 ***
## AntPost         -1.6588     0.0892 32502.6022 -18.596  < 2e-16 ***
## ---
## Signif. codes:  0 '***' 0.001 '**' 0.01 '*' 0.05 '.' 0.1 ' ' 1
##
## Correlation of Fixed Effects:
##          (Intr) SYNINC SEMINC
## SYNINCOR -0.228
## SEMINCOR -0.430  0.039
## AntPost  -0.135  0.000  0.000
```

Comparing those models shows that we should be using the last one

```
## Data: FWC_Mid
## Models:
## neg_basic: N400 ~ SYN + SEM + Ant + (Cond | Subj) + (cCibleP_COR + Cond |
## neg_basic:     Item)
## neg_basic3: N400 ~ SYN * SEM + Ant + (Cond | Subj) + (cCibleP_COR + Cond |
## neg_basic3:     Item)
##            Df    AIC    BIC  logLik deviance  Chisq Chi Df Pr(>Chisq)
## neg_basic  30 236585 236837 -118262   236525
## neg_basic3 31 236586 236847 -118262   236524 0.7768      1     0.3781
```

Anova wrapper

```
## Analysis of Deviance Table (Type III Wald chisquare tests)
##
## Response: N400
##                 Chisq Df Pr(>Chisq)
## (Intercept)  19.6333  1  9.382e-06 ***
## SYN           6.6233  1    0.01007 *
## SEM          27.0851  1  1.947e-07 ***
## Ant         345.8021  1  < 2.2e-16 ***
## ---
## Signif. codes:  0 '***' 0.001 '**' 0.01 '*' 0.05 '.' 0.1 ' ' 1
```

R-squared (marginal and conditional)

```
##             R2m       R2c
## [1,] 0.01436214 0.1120388
```

At Lateral electrodes

First, we ran the full model with factors Syntax (SYN), Semantics (SEM), Anteriority (Ant) and their interactions

```
## Linear mixed model fit by maximum likelihood . t-tests use
##   Satterthwaite's method [lmerModLmerTest]
## Formula: N400 ~ SYN * SEM * Ant * Hemi + (Cond | Subj) + (cCibleP_COR +
##     Cond | Item)
##    Data: FWC_Lat
##
##       AIC       BIC    logLik  deviance  df.resid
##  796063.7  796469.9 -397989.9  795979.7    117068
##
## Scaled residuals:
##     Min      1Q  Median      3Q     Max
## -6.1620 -0.6175 -0.0095  0.6134  6.2611
##
## Random effects:
##  Groups   Name        Variance Std.Dev. Corr
##  Item     (Intercept)  3.3481  1.8298
##           cCibleP_COR 76.8317  8.7654    0.34
##           CondSEM      5.3957  2.3229   -0.66  0.01
##           CondSYN      4.9189  2.2179   -0.64  0.02  0.61
##           CondSYNSEM   4.2259  2.0557   -0.62  0.11  0.61  0.63
##  Subj     (Intercept)  1.6321  1.2775
##           CondSEM      0.9215  0.9599   -0.25
##           CondSYN      0.4730  0.6877    0.18  0.09
##           CondSYNSEM   1.1798  1.0862   -0.17  0.45  0.68
##  Residual             51.4745  7.1746
## Number of obs: 117110, groups:  Item, 160; Subj, 36
##
## Fixed effects:
##                                       Estimate Std. Error         df
## (Intercept)                          1.541e+00  2.670e-01  7.768e+01
## SYNINCOR                            -5.095e-01  2.376e-01  1.916e+02
## SEMINCOR                            -9.566e-01  2.686e-01  1.521e+02
## AntPost                             -1.404e+00  1.188e-01  1.162e+05
## Hemiright                           -7.923e-01  1.100e-01  1.162e+05
## SYNINCOR:SEMINCOR                    1.073e-01  3.215e-01  2.043e+02
## SYNINCOR:AntPost                    -2.572e-01  1.682e-01  1.162e+05
## SEMINCOR:AntPost                    -9.788e-02  1.695e-01  1.162e+05
## SYNINCOR:Hemiright                  -7.848e-02  1.557e-01  1.162e+05
## SEMINCOR:Hemiright                   2.761e-02  1.570e-01  1.162e+05
## AntPost:Hemiright                    2.765e-01  1.680e-01  1.162e+05
## SYNINCOR:SEMINCOR:AntPost            1.807e-01  2.397e-01  1.162e+05
## SYNINCOR:SEMINCOR:Hemiright          2.074e-01  2.219e-01  1.162e+05
## SYNINCOR:AntPost:Hemiright           4.917e-02  2.379e-01  1.162e+05
## SEMINCOR:AntPost:Hemiright          -1.351e-01  2.398e-01  1.162e+05
## SYNINCOR:SEMINCOR:AntPost:Hemiright  2.103e-02  3.390e-01  1.162e+05
##                                     t value Pr(>|t|)
## (Intercept)                           5.769 1.55e-07 ***
## SYNINCOR                             -2.145 0.033224 *
## SEMINCOR                             -3.561 0.000494 ***
```

```
## AntPost                             -11.816  < 2e-16 ***
## Hemiright                            -7.204 5.89e-13 ***
## SYNINCOR:SEMINCOR                     0.334 0.738818
## SYNINCOR:AntPost                     -1.529 0.126233
## SEMINCOR:AntPost                     -0.577 0.563708
## SYNINCOR:Hemiright                   -0.504 0.614351
## SEMINCOR:Hemiright                    0.176 0.860368
## AntPost:Hemiright                     1.646 0.099731 .
## SYNINCOR:SEMINCOR:AntPost             0.754 0.450855
## SYNINCOR:SEMINCOR:Hemiright           0.935 0.349969
## SYNINCOR:AntPost:Hemiright            0.207 0.836281
## SEMINCOR:AntPost:Hemiright           -0.563 0.573205
## SYNINCOR:SEMINCOR:AntPost:Hemiright   0.062 0.950534
## ---
## Signif. codes:  0 '***' 0.001 '**' 0.01 '*' 0.05 '.' 0.1 ' ' 1
##
## Correlation matrix not shown by default, as p = 16 > 12.
## Use print(x, correlation=TRUE)  or
##   vcov(x)     if you need it
```

Then, we ran the same model, and remove the interaction with Anteriority (Ant)

```
## Linear mixed model fit by maximum likelihood . t-tests use
##   Satterthwaite's method [lmerModLmerTest]
## Formula: N400 ~ Hemi * SYN * SEM + Ant + (Cond | Subj) + (cCibleP_COR +
##     Cond | Item)
##    Data: FWC_Lat
##
##       AIC       BIC    logLik  deviance  df.resid
##  796063.0  796401.5 -397996.5  795993.0    117075
##
## Scaled residuals:
##     Min      1Q  Median      3Q     Max
## -6.1520 -0.6176 -0.0096  0.6133  6.2659
##
## Random effects:
##  Groups   Name        Variance Std.Dev. Corr
##  Item     (Intercept)  3.3481  1.8298
##           cCibleP_COR 76.8329  8.7654    0.34
##           CondSEM      5.3955  2.3228   -0.66  0.01
##           CondSYN      4.9187  2.2178   -0.64  0.02  0.61
##           CondSYNSEM   4.2258  2.0557   -0.62  0.11  0.61  0.63
##  Subj     (Intercept)  1.6321  1.2775
##           CondSEM      0.9214  0.9599   -0.25
##           CondSYN      0.4730  0.6877    0.18  0.09
##           CondSYNSEM   1.1797  1.0862   -0.17  0.45  0.68
##  Residual             51.4804  7.1750
## Number of obs: 117110, groups:  Item, 160; Subj, 36
##
## Fixed effects:
##                              Estimate Std. Error         df t value
## (Intercept)                 1.546e+00  2.628e-01  7.283e+01   5.883
## Hemiright                  -6.737e-01  8.314e-02  1.162e+05  -8.104
## SYNINCOR                   -6.198e-01  2.264e-01  1.580e+02  -2.738
```

```
## SEMINCOR                    -9.985e-01  2.586e-01  1.307e+02  -3.861
## AntPost                     -1.416e+00  4.237e-02  1.162e+05 -33.419
## Hemiright:SYNINCOR          -5.741e-02  1.177e-01  1.162e+05  -0.488
## Hemiright:SEMINCOR          -3.028e-02  1.187e-01  1.162e+05  -0.255
## SYNINCOR:SEMINCOR            1.848e-01  3.046e-01  1.647e+02   0.607
## Hemiright:SYNINCOR:SEMINCOR  2.164e-01  1.678e-01  1.162e+05   1.290
##                             Pr(>|t|)
## (Intercept)                 1.14e-07 ***
## Hemiright                   5.38e-16 ***
## SYNINCOR                    0.006890 **
## SEMINCOR                    0.000177 ***
## AntPost                      < 2e-16 ***
## Hemiright:SYNINCOR          0.625857
## Hemiright:SEMINCOR          0.798609
## SYNINCOR:SEMINCOR           0.544977
## Hemiright:SYNINCOR:SEMINCOR 0.197025
## ---
## Signif. codes:  0 '***' 0.001 '**' 0.01 '*' 0.05 '.' 0.1 ' ' 1
##
## Correlation of Fixed Effects:
##             (Intr) Hmrght SYNINCOR SEMINC AntPst Hm:SYNINCOR H:SEMI
## Hemiright   -0.158
## SYNINCOR    -0.265  0.184
## SEMINCOR    -0.438  0.161  0.428
## AntPost     -0.069  0.000  0.000    0.000
## Hm:SYNINCOR  0.112 -0.706 -0.260   -0.113  0.000
## Hm:SEMINCOR  0.111 -0.701 -0.129   -0.229  0.000  0.495
## SYNINCOR:SE  0.243 -0.136 -0.586   -0.722  0.000  0.193       0.195
## H:SYNINCOR: -0.078  0.496  0.183    0.162  0.000 -0.702      -0.707
##             SYNINCOR:
## Hemiright
## SYNINCOR
## SEMINCOR
## AntPost
## Hm:SYNINCOR
## Hm:SEMINCOR
## SYNINCOR:SE
## H:SYNINCOR: -0.275
```

Comparing those models shows that we should be using the second one

```
## Data: FWC_Lat
## Models:
## neg_basicL3: N400 ~ Hemi * SYN * SEM + Ant + (Cond | Subj) + (cCibleP_COR +
## neg_basicL3:     Cond | Item)
## neg_basicL2: N400 ~ SYN * SEM * Ant * Hemi + (Cond | Subj) + (cCibleP_COR +
## neg_basicL2:     Cond | Item)
##             Df    AIC    BIC  logLik deviance  Chisq Chi Df Pr(>Chisq)
## neg_basicL3 35 796063 796402 -397997   795993
## neg_basicL2 42 796064 796470 -397990   795980 13.304      7    0.06503 .
## ---
## Signif. codes:  0 '***' 0.001 '**' 0.01 '*' 0.05 '.' 0.1 ' ' 1
```

Then, we removed the interaction with Semantics (SEM)

```
## Linear mixed model fit by maximum likelihood . t-tests use
```

```
##   Satterthwaite's method [lmerModLmerTest]
## Formula: N400 ~ Hemi * SYN + SEM + Ant + (Cond | Subj) + (cCibleP_COR +
##     Cond | Item)
##    Data: FWC_Lat
##
##       AIC       BIC    logLik  deviance  df.resid
##  796060.6  796370.0 -397998.3  795996.6    117078
##
## Scaled residuals:
##     Min      1Q  Median      3Q     Max
## -6.1576 -0.6180 -0.0093  0.6131  6.2661
##
## Random effects:
##  Groups   Name        Variance Std.Dev. Corr
##  Item     (Intercept)  3.3480  1.8297
##           cCibleP_COR 76.8199  8.7647    0.34
##           CondSEM      5.4151  2.3270   -0.66  0.02
##           CondSYN      4.9373  2.2220   -0.64  0.02  0.62
##           CondSYNSEM   4.2265  2.0559   -0.62  0.11  0.61  0.63
##  Subj     (Intercept)  1.6328  1.2778
##           CondSEM      0.9263  0.9624   -0.25
##           CondSYN      0.4737  0.6882    0.18  0.09
##           CondSYNSEM   1.1805  1.0865   -0.18  0.45  0.69
##  Residual             51.4816  7.1751
## Number of obs: 117110, groups:  Item, 160; Subj, 36
##
## Fixed effects:
##                      Estimate Std. Error         df t value Pr(>|t|)
## (Intercept)         1.493e+00  2.541e-01  6.098e+01   5.875 1.89e-07 ***
## Hemiright          -6.886e-01  5.932e-02  1.162e+05 -11.608  < 2e-16 ***
## SYNINCOR           -5.471e-01  1.835e-01  9.748e+01  -2.981  0.00363 **
## SEMINCOR           -8.313e-01  1.738e-01  8.374e+01  -4.784 7.28e-06 ***
## AntPost            -1.416e+00  4.237e-02  1.162e+05 -33.419  < 2e-16 ***
## Hemiright:SYNINCOR  4.948e-02  8.387e-02  1.162e+05   0.590  0.55522
## ---
## Signif. codes:  0 '***' 0.001 '**' 0.01 '*' 0.05 '.' 0.1 ' ' 1
##
## Correlation of Fixed Effects:
##             (Intr) Hmrght SYNINC SEMINC AntPst
## Hemiright   -0.117
## SYNINCOR    -0.157  0.162
## SEMINCOR    -0.386  0.000  0.012
## AntPost     -0.071  0.000  0.000  0.000
## Hm:SYNINCOR  0.083 -0.707 -0.228  0.000  0.000
```

Comparing those models shows that we should be using the second one

```
## Data: FWC_Lat
## Models:
## neg_basicL4: N400 ~ Hemi * SYN + SEM + Ant + (Cond | Subj) + (cCibleP_COR +
## neg_basicL4:     Cond | Item)
## neg_basicL3: N400 ~ Hemi * SYN * SEM + Ant + (Cond | Subj) + (cCibleP_COR +
## neg_basicL3:     Cond | Item)
##             Df    AIC    BIC  logLik deviance  Chisq Chi Df Pr(>Chisq)
## neg_basicL4 32 796061 796370 -397998   795997
```

```
## neg_basicL3 35 796063 796402 -397997   795993 3.5264      3     0.3174
```

Then, we removed the remaining interaction between Syntax (SYN) and Hemishpere

```
## Linear mixed model fit by maximum likelihood . t-tests use
##   Satterthwaite's method [lmerModLmerTest]
## Formula: N400 ~ Hemi + SYN + SEM + Ant + (Cond | Subj) + (cCibleP_COR +
##     Cond | Item)
##    Data: FWC_Lat
##
##       AIC       BIC    logLik  deviance  df.resid
##  796058.9  796358.7 -397998.5  795996.9    117079
##
## Scaled residuals:
##     Min      1Q  Median      3Q     Max
## -6.1593 -0.6179 -0.0094  0.6134  6.2678
##
## Random effects:
##  Groups   Name        Variance Std.Dev. Corr
##  Item     (Intercept)  3.3479  1.8297
##           cCibleP_COR 76.8194  8.7647    0.34
##           CondSEM      5.4151  2.3270   -0.66  0.02
##           CondSYN      4.9373  2.2220   -0.64  0.02  0.62
##           CondSYNSEM   4.2266  2.0559   -0.62  0.11  0.61  0.63
##  Subj     (Intercept)  1.6328  1.2778
##           CondSEM      0.9262  0.9624   -0.25
##           CondSYN      0.4737  0.6882    0.18  0.09
##           CondSYNSEM   1.1805  1.0865   -0.18  0.45  0.69
##  Residual             51.4817  7.1751
## Number of obs: 117110, groups:  Item, 160; Subj, 36
##
## Fixed effects:
##               Estimate Std. Error         df t value Pr(>|t|)
## (Intercept)  1.480e+00  2.532e-01  6.015e+01   5.846 2.20e-07 ***
## Hemiright   -6.638e-01  4.193e-02  1.162e+05 -15.831  < 2e-16 ***
## SYNINCOR    -5.223e-01  1.787e-01  8.756e+01  -2.923  0.00441 **
## SEMINCOR    -8.313e-01  1.738e-01  8.374e+01  -4.784 7.28e-06 ***
## AntPost     -1.416e+00  4.237e-02  1.162e+05 -33.419  < 2e-16 ***
## ---
## Signif. codes:  0 '***' 0.001 '**' 0.01 '*' 0.05 '.' 0.1 ' ' 1
##
## Correlation of Fixed Effects:
##           (Intr) Hmrght SYNINC SEMINC
## Hemiright -0.083
## SYNINCOR  -0.142  0.000
## SEMINCOR  -0.387  0.000  0.012
## AntPost   -0.072  0.000  0.000  0.000
```

Comparing those models shows that we should be using the second one

```
## Data: FWC_Lat
## Models:
## neg_basic_L: N400 ~ Hemi + SYN + SEM + Ant + (Cond | Subj) + (cCibleP_COR +
## neg_basic_L:     Cond | Item)
## neg_basicL4: N400 ~ Hemi * SYN + SEM + Ant + (Cond | Subj) + (cCibleP_COR +
## neg_basicL4:     Cond | Item)
```

```
##              Df    AIC    BIC  logLik deviance  Chisq Chi Df Pr(>Chisq)
## neg_basic_L 31 796059 796359 -397998   795997
## neg_basicL4 32 796061 796370 -397998   795997 0.3481      1     0.5552
```

Anova wrapper

```
## Analysis of Deviance Table (Type III Wald chisquare tests)
##
## Response: N400
##                Chisq Df Pr(>Chisq)
## (Intercept)   34.180  1  5.024e-09 ***
## Hemi         250.619  1  < 2.2e-16 ***
## SYN            8.546  1   0.003463 **
## SEM           22.890  1  1.716e-06 ***
## Ant         1116.802  1  < 2.2e-16 ***
## ---
## Signif. codes:  0 '***' 0.001 '**' 0.01 '*' 0.05 '.' 0.1 ' ' 1
```

R-squared (marginal and conditional)

```
##             R2m       R2c
## [1,] 0.01367494 0.1646921
```

Model with Condition and Electrode as factors (using difference waves)

```
## Linear mixed model fit by maximum likelihood . t-tests use
##   Satterthwaite's method [lmerModLmerTest]
## Formula: N400 ~ Cond * Elec + (Cond | Subj)
##    Data: N400_long
##
##      AIC      BIC   logLik deviance df.resid
##   4966.4   5211.2  -2437.2   4874.4     1466
##
## Scaled residuals:
##     Min      1Q  Median      3Q     Max
## -4.7855 -0.5857  0.0203  0.5795  3.8555
##
## Random effects:
##  Groups   Name        Variance Std.Dev. Corr
##  Subj     (Intercept) 0.8472   0.9205
##           CondSYN     1.3614   1.1668   -0.72
##  Residual             1.3014   1.1408
## Number of obs: 1512, groups:  Subj, 36
##
## Fixed effects:
##                   Estimate Std. Error         df t value Pr(>|t|)
## (Intercept)     -1.232e+00  2.443e-01  1.921e+02  -5.042 1.06e-06 ***
## CondSYN          3.803e-01  3.318e-01  2.397e+02   1.146  0.25290
## ElecC4          -1.732e-02  2.689e-01  1.440e+03  -0.064  0.94864
## ElecCz          -2.296e-01  2.689e-01  1.440e+03  -0.854  0.39335
## ElecF3           1.573e-01  2.689e-01  1.440e+03   0.585  0.55871
## ElecF4           3.330e-02  2.689e-01  1.440e+03   0.124  0.90145
## ElecF7           6.702e-01  2.689e-01  1.440e+03   2.493  0.01279 *
## ElecF8           7.688e-01  2.689e-01  1.440e+03   2.859  0.00431 **
## ElecFp1          1.519e+00  2.689e-01  1.440e+03   5.649 1.95e-08 ***
```

```
## ElecFp2          4.360e-01  2.689e-01  1.440e+03   1.621  0.10514
## ElecFpz          5.464e-01  2.689e-01  1.440e+03   2.032  0.04232 *
## ElecFz          -5.841e-02  2.689e-01  1.440e+03  -0.217  0.82805
## ElecO1           3.060e-01  2.689e-01  1.440e+03   1.138  0.25534
## ElecO2           2.535e-01  2.689e-01  1.440e+03   0.943  0.34591
## ElecOz           2.252e-01  2.689e-01  1.440e+03   0.838  0.40238
## ElecP3           8.133e-03  2.689e-01  1.440e+03   0.030  0.97587
## ElecP4          -7.343e-02  2.689e-01  1.440e+03  -0.273  0.78483
## ElecPz          -1.439e-02  2.689e-01  1.440e+03  -0.054  0.95733
## ElecT3           5.335e-01  2.689e-01  1.440e+03   1.984  0.04742 *
## ElecT4           4.881e-01  2.689e-01  1.440e+03   1.815  0.06969 .
## ElecT5           4.532e-01  2.689e-01  1.440e+03   1.686  0.09210 .
## ElecT6           1.620e-01  2.689e-01  1.440e+03   0.602  0.54698
## CondSYN:ElecC4  -1.428e-01  3.803e-01  1.440e+03  -0.376  0.70733
## CondSYN:ElecCz   2.155e-01  3.803e-01  1.440e+03   0.567  0.57093
## CondSYN:ElecF3   2.222e-01  3.803e-01  1.440e+03   0.584  0.55903
## CondSYN:ElecF4   7.218e-02  3.803e-01  1.440e+03   0.190  0.84948
## CondSYN:ElecF7   8.403e-02  3.803e-01  1.440e+03   0.221  0.82514
## CondSYN:ElecF8  -1.210e-01  3.803e-01  1.440e+03  -0.318  0.75034
## CondSYN:ElecFp1 -2.592e-01  3.803e-01  1.440e+03  -0.682  0.49550
## CondSYN:ElecFp2  6.721e-02  3.803e-01  1.440e+03   0.177  0.85974
## CondSYN:ElecFpz  2.182e-01  3.803e-01  1.440e+03   0.574  0.56620
## CondSYN:ElecFz   3.071e-01  3.803e-01  1.440e+03   0.808  0.41942
## CondSYN:ElecO1  -2.207e-01  3.803e-01  1.440e+03  -0.581  0.56166
## CondSYN:ElecO2  -1.161e-01  3.803e-01  1.440e+03  -0.305  0.76016
## CondSYN:ElecOz  -8.730e-02  3.803e-01  1.440e+03  -0.230  0.81845
## CondSYN:ElecP3  -5.935e-02  3.803e-01  1.440e+03  -0.156  0.87599
## CondSYN:ElecP4  -3.589e-02  3.803e-01  1.440e+03  -0.094  0.92482
## CondSYN:ElecPz   6.257e-02  3.803e-01  1.440e+03   0.165  0.86932
## CondSYN:ElecT3  -2.850e-01  3.803e-01  1.440e+03  -0.749  0.45375
## CondSYN:ElecT4  -2.142e-01  3.803e-01  1.440e+03  -0.563  0.57323
## CondSYN:ElecT5  -4.329e-01  3.803e-01  1.440e+03  -1.139  0.25508
## CondSYN:ElecT6  -1.964e-01  3.803e-01  1.440e+03  -0.516  0.60568
## ---
## Signif. codes:  0 '***' 0.001 '**' 0.01 '*' 0.05 '.' 0.1 ' ' 1
```

```
##
## Correlation matrix not shown by default, as p = 42 > 12.
## Use print(x, correlation=TRUE)  or
##   vcov(x)     if you need it
```

Anova wrapper

```
## Analysis of Deviance Table (Type III Wald chisquare tests)
##
## Response: N400
##               Chisq Df Pr(>Chisq)
## (Intercept) 25.4198  1  4.612e-07 ***
## Cond         1.3135  1     0.2518
## Elec        85.0795 20  5.304e-10 ***
## Cond:Elec   10.3458 20     0.9615
## ---
## Signif. codes:  0 '***' 0.001 '**' 0.01 '*' 0.05 '.' 0.1 ' ' 1
```

R-squared (marginal and conditional)

```
##             R2m       R2c
## [1,] 0.07301721 0.4143312
```

Model evaluating the effect of Cloze probability at central electrodes

```
## Linear mixed model fit by maximum likelihood . t-tests use
##   Satterthwaite's method [lmerModLmerTest]
## Formula: N400 ~ SYN * SEM * cCibleP_COR + (1 | Subj) + (1 | Item)
##    Data: N400_C
##
##      AIC      BIC   logLik deviance df.resid
##  51729.4  51805.3 -25853.7  51707.4     7269
##
## Scaled residuals:
##     Min      1Q  Median      3Q     Max
## -4.1686 -0.6414 -0.0022  0.6320  4.1030
##
## Random effects:
##  Groups   Name        Variance Std.Dev.
##  Item     (Intercept)  0.7829  0.8848
##  Subj     (Intercept)  3.5774  1.8914
##  Residual             69.6830  8.3476
## Number of obs: 7280, groups:  Item, 160; Subj, 36
##
## Fixed effects:
##                                Estimate Std. Error         df t value
## (Intercept)                     1.16890    0.37809   62.10252   3.092
## SYNINCOR                       -0.86056    0.27626 7134.26968  -3.115
## SEMINCOR                       -1.57610    0.27810 7150.67984  -5.667
## cCibleP_COR                     1.97773    0.82864 5031.40792   2.387
## SYNINCOR:SEMINCOR               0.28354    0.39336 7153.03255   0.721
## SYNINCOR:cCibleP_COR            0.08606    1.14083 7124.25459   0.075
## SEMINCOR:cCibleP_COR           -2.99549    1.14871 7133.47776  -2.608
## SYNINCOR:SEMINCOR:cCibleP_COR   0.49445    1.62440 7135.18279   0.304
##                               Pr(>|t|)
## (Intercept)                    0.00298 **
## SYNINCOR                       0.00185 **
## SEMINCOR                      1.51e-08 ***
## cCibleP_COR                    0.01704 *
## SYNINCOR:SEMINCOR              0.47105
## SYNINCOR:cCibleP_COR           0.93987
## SEMINCOR:cCibleP_COR           0.00913 **
## SYNINCOR:SEMINCOR:cCibleP_COR  0.76084
## ---
## Signif. codes:  0 '***' 0.001 '**' 0.01 '*' 0.05 '.' 0.1 ' ' 1
##
## Correlation of Fixed Effects:
##                   (Intr) SYNINCOR SEMINCOR cCP_CO SYNINCOR:SEMINCOR
## SYNINCOR          -0.362
## SEMINCOR          -0.360  0.494
## cCibleP_COR       -0.032  0.045    0.046
## SYNINCOR:SEMINCOR  0.255 -0.703   -0.707   -0.031
## SYNINCOR:CP        0.023 -0.076   -0.034   -0.690  0.054
```

```
## SEMINCOR:CP         0.025 -0.033   -0.075   -0.685  0.053
## SYNINCOR:SEMINCOR: -0.016  0.053    0.054    0.486 -0.078
##                    SYNINCOR:C SEMINCOR:
## SYNINCOR
## SEMINCOR
## cCibleP_COR
## SYNINCOR:SEMINCOR
## SYNINCOR:CP
## SEMINCOR:CP         0.498
## SYNINCOR:SEMINCOR: -0.703     -0.707
```

Anova wrapper

```
## Analysis of Deviance Table (Type III Wald chisquare tests)
##
## Response: N400
##                       Chisq Df Pr(>Chisq)
## (Intercept)          9.5578  1   0.001991 **
## SYN                  9.7035  1   0.001839 **
## SEM                 32.1193  1   1.45e-08 ***
## cCibleP_COR          5.6964  1   0.017000 *
## SYN:SEM              0.5196  1   0.471027
## SYN:cCibleP_COR      0.0057  1   0.939866
## SEM:cCibleP_COR      6.8001  1   0.009115 **
## SYN:SEM:cCibleP_COR  0.0927  1   0.760830
## ---
## Signif. codes:  0 '***' 0.001 '**' 0.01 '*' 0.05 '.' 0.1 ' ' 1
```

R-squared (marginal and conditional)

```
##             R2m        R2c
## [1,] 0.01101343 0.06925366
```

Regression tree

```
## Loading required package: grid

## Loading required package: libcoin

## Loading required package: mvtnorm
```

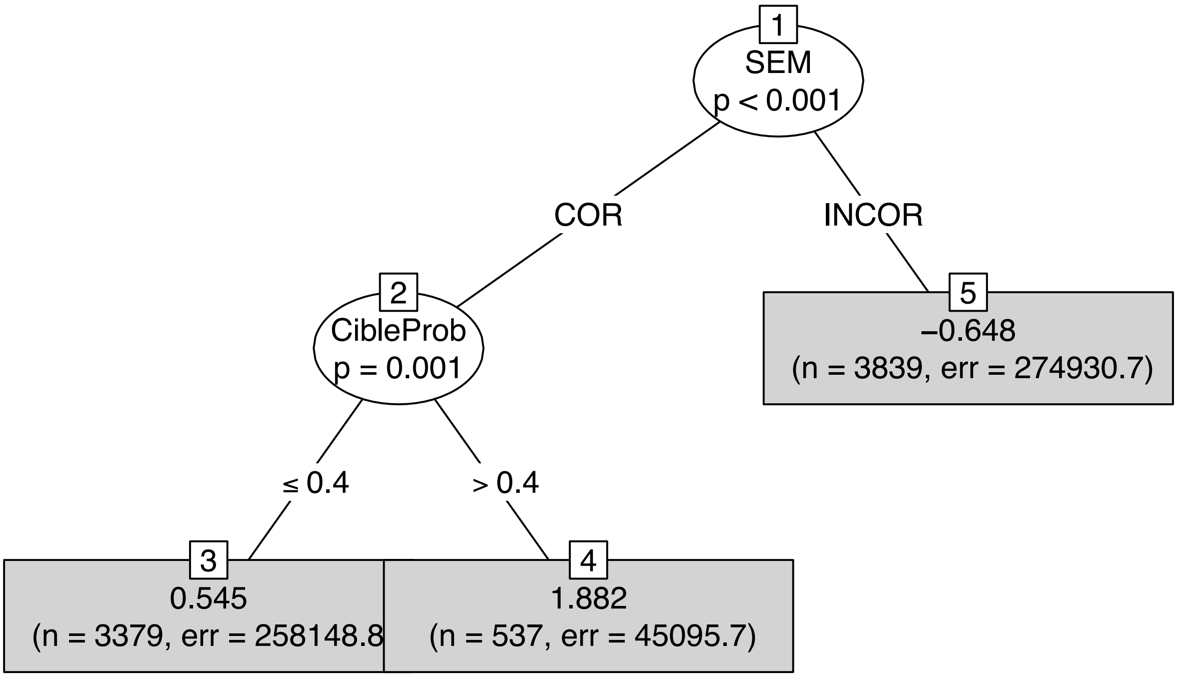

C. P3a: Time window 550 ms - 650 ms

At Midline electrodes

```
## Linear mixed model fit by maximum likelihood . t-tests use
##   Satterthwaite's method [lmerModLmerTest]
## Formula: eP600 ~ SYN * SEM * Ant + (Cond | Subj) + (cCibleP_COR + Cond |
##     Item)
##    Data: FWC_Mid
##
##       AIC       BIC    logLik  deviance  df.resid
##  243039.3  243325.5 -121485.7  242971.3     33426
##
## Scaled residuals:
##     Min      1Q  Median      3Q     Max
## -4.4882 -0.6352 -0.0080  0.6323  4.5486
##
## Random effects:
##  Groups   Name        Variance Std.Dev. Corr
##  Item     (Intercept)  3.876   1.969
##           cCibleP_COR 18.979   4.356    -0.06
##           CondSEM      7.728   2.780    -0.73  0.04
##           CondSYN      7.707   2.776    -0.73  0.11  0.55
##           CondSYNSEM   9.699   3.114    -0.77  0.12  0.63  0.66
##  Subj     (Intercept)  2.659   1.631
##           CondSEM      1.417   1.190    -0.29
##           CondSYN      3.719   1.929     0.05  0.46
##           CondSYNSEM   5.122   2.263     0.04  0.49  0.70
##  Residual             80.576   8.976
## Number of obs: 33460, groups:  Item, 160; Subj, 36
```

```
##
## Fixed effects:
##                           Estimate Std. Error         df t value
## (Intercept)              1.812e+00  3.461e-01  6.988e+01   5.235
## SYNINCOR                 2.051e+00  4.376e-01  7.549e+01   4.687
## SEMINCOR                 1.031e-01  3.579e-01  1.208e+02   0.288
## AntPost                 -2.242e-01  1.946e-01  3.251e+04  -1.152
## SYNINCOR:SEMINCOR       -8.713e-01  5.039e-01  8.793e+01  -1.729
## SYNINCOR:AntPost        -5.071e-01  2.756e-01  3.251e+04  -1.840
## SEMINCOR:AntPost         7.684e-02  2.777e-01  3.251e+04   0.277
## SYNINCOR:SEMINCOR:AntPost  9.687e-01  3.926e-01  3.251e+04   2.467
##                         Pr(>|t|)
## (Intercept)             1.65e-06 ***
## SYNINCOR                1.20e-05 ***
## SEMINCOR                  0.7738
## AntPost                   0.2492
## SYNINCOR:SEMINCOR         0.0873 .
## SYNINCOR:AntPost          0.0658 .
## SEMINCOR:AntPost          0.7820
## SYNINCOR:SEMINCOR:AntPost   0.0136 *
## ---
## Signif. codes:  0 '***' 0.001 '**' 0.01 '*' 0.05 '.' 0.1 ' ' 1
##
## Correlation of Fixed Effects:
##                   (Intr) SYNINCOR SEMINCOR AntPst SYNINCOR:SEMINCOR
## SYNINCOR          -0.268
## SEMINCOR          -0.489  0.486
## AntPost           -0.281  0.222    0.272
## SYNINCOR:SEMINCOR   0.317 -0.577   -0.631   -0.193
## SYNINCOR:AP        0.198 -0.315   -0.192   -0.706  0.273
## SEMINCOR:AP        0.197 -0.156   -0.388   -0.701  0.276
## SYNINCOR:SEMINCOR: -0.139  0.221    0.274    0.496 -0.390
##                   SYNINCOR:A SEMINCOR:
## SYNINCOR
## SEMINCOR
## AntPost
## SYNINCOR:SEMINCOR
## SYNINCOR:AP
## SEMINCOR:AP        0.495
## SYNINCOR:SEMINCOR: -0.702     -0.707
```

Anova wrapper

```
## Analysis of Deviance Table (Type III Wald chisquare tests)
##
## Response: eP600
##               Chisq Df Pr(>Chisq)
## (Intercept) 27.4035  1  1.651e-07 ***
## SYN         21.9710  1  2.768e-06 ***
## SEM          0.0830  1    0.77328
## Ant          1.3280  1    0.24917
## SYN:SEM      2.9903  1    0.08377 .
## SYN:Ant      3.3861  1    0.06575 .
## SEM:Ant      0.0765  1    0.78204
## SYN:SEM:Ant  6.0877  1    0.01361 *
```

```
## ---
## Signif. codes:  0 '***' 0.001 '**' 0.01 '*' 0.05 '.' 0.1 ' ' 1
```

R-squared (marginal and conditional)

```
##              R2m       R2c
## [1,] 0.007679117 0.1162467
```

Follow-up models splitting by levels of Semantics

Semantics = correct

```
## Linear mixed model fit by maximum likelihood . t-tests use
##   Satterthwaite's method [lmerModLmerTest]
## Formula: eP600 ~ SYN * Ant + (Cond | Subj) + (cCibleP_COR + Cond | Item)
##    Data: subset(FWC_Mid, SEM == "COR")
##
##      AIC      BIC   logLik deviance df.resid
## 123683.8 123792.1 -61827.9 123655.8    16962
##
## Scaled residuals:
##     Min      1Q  Median      3Q     Max
## -4.4236 -0.6393 -0.0051  0.6274  3.8503
##
## Random effects:
##  Groups   Name        Variance Std.Dev. Corr
##  Item     (Intercept)  3.981   1.995
##           cCibleP_COR 27.457   5.240    -0.14
##           CondSYN      7.717   2.778    -0.75  0.15
##  Subj     (Intercept)  2.689   1.640
##           CondSYN      3.676   1.917    0.06
##  Residual             82.194   9.066
## Number of obs: 16976, groups:  Item, 160; Subj, 36
##
## Fixed effects:
##                    Estimate Std. Error         df t value Pr(>|t|)
## (Intercept)          1.7937     0.3506    71.0652   5.116 2.55e-06 ***
## SYNINCOR             2.0199     0.4373    76.1460   4.619 1.54e-05 ***
## AntPost             -0.2242     0.1965 16468.7446  -1.141   0.2539
## SYNINCOR:AntPost    -0.5071     0.2783 16468.7446  -1.822   0.0685 .
## ---
## Signif. codes:  0 '***' 0.001 '**' 0.01 '*' 0.05 '.' 0.1 ' ' 1
##
## Correlation of Fixed Effects:
##             (Intr) SYNINCOR AntPst
## SYNINCOR    -0.267
## AntPost     -0.280  0.225
## SYNINCOR:AP  0.198 -0.318   -0.706
```

Anova wrapper

```
## Analysis of Deviance Table (Type III Wald chisquare tests)
##
## Response: eP600
##              Chisq Df Pr(>Chisq)
```

```
## (Intercept) 26.1723  1  3.123e-07 ***
## SYN         21.3358  1  3.855e-06 ***
## Ant          1.3018  1    0.25388
## SYN:Ant      3.3194  1    0.06847 .
## ---
## Signif. codes:  0 '***' 0.001 '**' 0.01 '*' 0.05 '.' 0.1 ' ' 1
```

Semantics = anomalous

```
## Linear mixed model fit by maximum likelihood . t-tests use
##   Satterthwaite's method [lmerModLmerTest]
## Formula: eP600 ~ SYN * Ant + (Cond | Subj) + (cCibleP_COR + Cond | Item)
##    Data: subset(FWC_Mid, SEM == "INCOR")
##
##      AIC      BIC   logLik deviance df.resid
## 119352.6 119460.5 -59662.3 119324.6    16470
##
## Scaled residuals:
##     Min      1Q  Median      3Q     Max
## -4.1023 -0.6336 -0.0149  0.6288  4.6344
##
## Random effects:
##  Groups   Name        Variance Std.Dev. Corr
##  Item     (Intercept)  3.608   1.899
##           cCibleP_COR 13.298   3.647    -0.04
##           CondSYNSEM   6.699   2.588    -0.64 -0.19
##  Subj     (Intercept)  3.079   1.755
##           CondSYNSEM   3.971   1.993    0.16
##  Residual             78.544   8.863
## Number of obs: 16484, groups:  Item, 160; Subj, 36
##
## Fixed effects:
##                    Estimate Std. Error         df t value Pr(>|t|)
## (Intercept)          1.9261     0.3603    62.9184   5.346 1.33e-06 ***
## SYNINCOR             1.1495     0.4382    71.2131   2.623   0.0107 *
## AntPost             -0.1474     0.1956 15980.0164  -0.753   0.4512
## SYNINCOR:AntPost     0.4616     0.2761 15980.0164   1.672   0.0946 .
## ---
## Signif. codes:  0 '***' 0.001 '**' 0.01 '*' 0.05 '.' 0.1 ' ' 1
##
## Correlation of Fixed Effects:
##             (Intr) SYNINCOR AntPst
## SYNINCOR    -0.150
## AntPost     -0.272  0.223
## SYNINCOR:AP  0.192 -0.315   -0.709
```

Anova wrapper

```
## Analysis of Deviance Table (Type III Wald chisquare tests)
##
## Response: eP600
##               Chisq Df Pr(>Chisq)
## (Intercept) 28.5773  1  9.003e-08 ***
## SYN          6.8798  1   0.008718 **
## Ant          0.5676  1   0.451199
```

```
## SYN:Ant      2.7952  1   0.094545 .
## ---
## Signif. codes:  0 '***' 0.001 '**' 0.01 '*' 0.05 '.' 0.1 ' ' 1
```

At Lateral electrodes

```
## Linear mixed model fit by maximum likelihood . t-tests use
##   Satterthwaite's method [lmerModLmerTest]
## Formula: eP600 ~ SYN * SEM * Ant * Hemi + (Cond | Subj) + (cCibleP_COR +
##     Cond | Item)
##    Data: FWC_Lat
##
##       AIC       BIC    logLik  deviance  df.resid
##  818598.5  819004.6 -409257.2  818514.5    117068
##
## Scaled residuals:
##     Min      1Q  Median      3Q     Max
## -6.6979 -0.6263 -0.0181  0.6118  5.4797
##
## Random effects:
##  Groups   Name        Variance Std.Dev. Corr
##  Item     (Intercept)  3.3384  1.8271
##           cCibleP_COR 79.6321  8.9237    0.37
##           CondSEM      6.0762  2.4650   -0.61  0.07
##           CondSYN      5.8244  2.4134   -0.67  0.03  0.56
##           CondSYNSEM   7.0932  2.6633   -0.66  0.15  0.61  0.62
##  Subj     (Intercept)  1.5179  1.2320
##           CondSEM      0.9665  0.9831   -0.23
##           CondSYN      2.5881  1.6088    0.00  0.28
##           CondSYNSEM   3.1697  1.7804   -0.06  0.49  0.66
##  Residual             62.3852  7.8984
## Number of obs: 117110, groups:  Item, 160; Subj, 36
##
## Fixed effects:
##                                    Estimate Std. Error         df
## (Intercept)                       1.617e+00  2.625e-01  8.101e+01
## SYNINCOR                          1.134e+00  3.518e-01  8.295e+01
## SEMINCOR                          1.510e-02  2.834e-01  1.486e+02
## AntPost                           2.525e-01  1.308e-01  1.162e+05
## Hemiright                        -5.799e-01  1.211e-01  1.162e+05
## SYNINCOR:SEMINCOR                -7.022e-01  3.872e-01  1.253e+02
## SYNINCOR:AntPost                 -6.081e-02  1.852e-01  1.162e+05
## SEMINCOR:AntPost                  1.638e-01  1.866e-01  1.162e+05
## SYNINCOR:Hemiright                5.275e-02  1.715e-01  1.162e+05
## SEMINCOR:Hemiright                6.329e-02  1.728e-01  1.162e+05
## AntPost:Hemiright                 3.297e-01  1.849e-01  1.162e+05
## SYNINCOR:SEMINCOR:AntPost         6.178e-01  2.639e-01  1.162e+05
## SYNINCOR:SEMINCOR:Hemiright       4.110e-01  2.443e-01  1.162e+05
## SYNINCOR:AntPost:Hemiright        1.881e-01  2.619e-01  1.162e+05
## SEMINCOR:AntPost:Hemiright        5.644e-02  2.640e-01  1.162e+05
## SYNINCOR:SEMINCOR:AntPost:Hemiright -8.687e-02  3.732e-01  1.162e+05
##                                  t value Pr(>|t|)
## (Intercept)                        6.163 2.63e-08 ***
```

```
## SYNINCOR                              3.223  0.00182 **
## SEMINCOR                              0.053  0.95756
## AntPost                               1.931  0.05355 .
## Hemiright                            -4.790 1.67e-06 ***
## SYNINCOR:SEMINCOR                    -1.814  0.07212 .
## SYNINCOR:AntPost                     -0.328  0.74266
## SEMINCOR:AntPost                      0.877  0.38024
## SYNINCOR:Hemiright                    0.308  0.75835
## SEMINCOR:Hemiright                    0.366  0.71419
## AntPost:Hemiright                     1.783  0.07458 .
## SYNINCOR:SEMINCOR:AntPost             2.341  0.01922 *
## SYNINCOR:SEMINCOR:Hemiright           1.682  0.09252 .
## SYNINCOR:AntPost:Hemiright            0.718  0.47266
## SEMINCOR:AntPost:Hemiright            0.214  0.83068
## SYNINCOR:SEMINCOR:AntPost:Hemiright  -0.233  0.81592
## ---
## Signif. codes:  0 '***' 0.001 '**' 0.01 '*' 0.05 '.' 0.1 ' ' 1
##
## Correlation matrix not shown by default, as p = 16 > 12.
## Use print(x, correlation=TRUE)  or
##   vcov(x)     if you need it
```

Anova wrapper

```
## Analysis of Deviance Table (Type III Wald chisquare tests)
##
## Response: eP600
##                    Chisq Df Pr(>Chisq)
## (Intercept)      37.9803  1  7.146e-10 ***
## SYN              10.3848  1   0.001271 **
## SEM               0.0028  1   0.957491
## Ant               3.7269  1   0.053543 .
## Hemi             22.9418  1  1.670e-06 ***
## SYN:SEM           3.2894  1   0.069727 .
## SYN:Ant           0.1078  1   0.742657
## SEM:Ant           0.7699  1   0.380241
## SYN:Hemi          0.0946  1   0.758352
## SEM:Hemi          0.1341  1   0.714189
## Ant:Hemi          3.1792  1   0.074581 .
## SYN:SEM:Ant       5.4815  1   0.019219 *
## SYN:SEM:Hemi      2.8301  1   0.092514 .
## SYN:Ant:Hemi      0.5157  1   0.472662
## SEM:Ant:Hemi      0.0457  1   0.830677
## SYN:SEM:Ant:Hemi  0.0542  1   0.815924
## ---
## Signif. codes:  0 '***' 0.001 '**' 0.01 '*' 0.05 '.' 0.1 ' ' 1
```

R-squared (marginal and conditional)

```
##              R2m      R2c
## [1,] 0.006162623 0.153648
```

Follow-up models splitting by levels of Syntax

Syntax = correct

```
## Linear mixed model fit by maximum likelihood . t-tests use
##   Satterthwaite's method [lmerModLmerTest]
## Formula: eP600 ~ SEM * Ant + (Cond | Subj) + (cCibleP_COR + Cond | Item)
##    Data: subset(FWC_Lat, SYN == "COR")
##
##       AIC       BIC    logLik  deviance  df.resid
##  501692.1  501820.5 -250832.0  501664.1     71046
##
## Scaled residuals:
##     Min      1Q  Median      3Q     Max
## -5.7733 -0.6099 -0.0101  0.6046  6.6736
##
## Random effects:
##  Groups   Name        Variance Std.Dev. Corr
##  Item     (Intercept)  2.9522  1.7182
##           cCibleP_COR 72.3207  8.5042    0.34
##           CondSEM      5.4465  2.3338   -0.66 -0.09
##  Subj     (Intercept)  1.3366  1.1561
##           CondSEM      0.7878  0.8876   -0.20
##  Residual             67.0521  8.1885
## Number of obs: 71060, groups:  Item, 160; Subj, 36
##
## Fixed effects:
##                    Estimate Std. Error         df t value Pr(>|t|)
## (Intercept)       1.409e+00  2.435e-01  7.564e+01   5.785 1.54e-07 ***
## SEMINCOR         -3.010e-03  2.537e-01  1.332e+02  -0.012    0.991
## AntPost          -6.006e-02  8.625e-02  7.048e+04  -0.696    0.486
## SEMINCOR:AntPost  1.070e-01  1.231e-01  7.048e+04   0.869    0.385
## ---
## Signif. codes:  0 '***' 0.001 '**' 0.01 '*' 0.05 '.' 0.1 ' ' 1
##
## Correlation of Fixed Effects:
##             (Intr) SEMINCOR AntPst
## SEMINCOR    -0.412
## AntPost     -0.188  0.180
## SEMINCOR:AP  0.131 -0.257   -0.701
```

Anova wrapper

```
## Analysis of Deviance Table (Type III Wald chisquare tests)
##
## Response: eP600
##               Chisq Df Pr(>Chisq)
## (Intercept) 33.4698  1  7.238e-09 ***
## SEM          0.0001  1     0.9905
## Ant          0.4848  1     0.4862
## SEM:Ant      0.7559  1     0.3846
## ---
## Signif. codes:  0 '***' 0.001 '**' 0.01 '*' 0.05 '.' 0.1 ' ' 1
```

Syntax = incorrect

```
## Linear mixed model fit by maximum likelihood . t-tests use
```

```
##   Satterthwaite's method [lmerModLmerTest]
## Formula: eP600 ~ SEM * Ant + (Cond | Subj) + (cCibleP_COR + Cond | Item)
##    Data: subset(FWC_Lat, SYN == "INCOR")
##
##       AIC       BIC    logLik  deviance  df.resid
##  506350.4  506478.9 -253161.2  506322.4     71131
##
## Scaled residuals:
##     Min      1Q  Median      3Q     Max
## -7.1442 -0.6154 -0.0062  0.6147  5.2790
##
## Random effects:
##  Groups   Name        Variance Std.Dev. Corr
##  Item     (Intercept)   3.526   1.878
##           cCibleP_COR 140.813  11.866    0.42
##           CondSYNSEM    4.499   2.121   -0.42  0.23
##  Subj     (Intercept)   3.513   1.874
##           CondSYNSEM    2.001   1.414   -0.25
##  Residual              70.892   8.420
## Number of obs: 71145, groups:  Item, 160; Subj, 36
##
## Fixed effects:
##                    Estimate Std. Error         df t value Pr(>|t|)
## (Intercept)       2.431e+00  3.505e-01  5.269e+01   6.934 5.91e-09 ***
## SEMINCOR         -4.340e-01  3.033e-01  6.929e+01  -1.431  0.15695
## AntPost          -2.168e-01  8.894e-02  7.060e+04  -2.438  0.01479 *
## SEMINCOR:AntPost  3.385e-01  1.265e-01  7.060e+04   2.676  0.00744 **
## ---
## Signif. codes:  0 '***' 0.001 '**' 0.01 '*' 0.05 '.' 0.1 ' ' 1
##
## Correlation of Fixed Effects:
##             (Intr) SEMINCOR AntPst
## SEMINCOR    -0.342
## AntPost     -0.134  0.155
## SEMINCOR:AP  0.094 -0.221   -0.703
```

Anova wrapper

```
## Analysis of Deviance Table (Type III Wald chisquare tests)
##
## Response: eP600
##               Chisq Df Pr(>Chisq)
## (Intercept) 48.0866  1  4.078e-12 ***
## SEM          2.0475  1   0.152460
## Ant          5.9417  1   0.014787 *
## SEM:Ant      7.1629  1   0.007443 **
## ---
## Signif. codes:  0 '***' 0.001 '**' 0.01 '*' 0.05 '.' 0.1 ' ' 1
```

D. P600: Time window 800 ms - 1200 ms

At Midline electrodes

```
## Linear mixed model fit by maximum likelihood . t-tests use
```

```
##   Satterthwaite's method [lmerModLmerTest]
## Formula: lP600 ~ SYN * SEM * Ant + (Cond | Subj) + (cCibleP_COR + Cond |
##     Item)
##    Data: FWC_Mid
##
##       AIC       BIC    logLik  deviance  df.resid
##  233857.5  234143.7 -116894.7  233789.5     33426
##
## Scaled residuals:
##     Min      1Q  Median      3Q     Max
## -5.2582 -0.6244 -0.0030  0.6236  6.3541
##
## Random effects:
##  Groups   Name        Variance Std.Dev. Corr
##  Item     (Intercept)  3.136   1.771
##           cCibleP_COR 14.805   3.848    -0.26
##           CondSEM      4.979   2.231    -0.67  0.29
##           CondSYN      5.388   2.321    -0.69  0.21  0.44
##           CondSYNSEM   6.686   2.586    -0.75  0.25  0.51  0.50
##  Subj     (Intercept)  2.167   1.472
##           CondSEM      2.162   1.470    -0.54
##           CondSYN      4.351   2.086    -0.36  0.79
##           CondSYNSEM   4.646   2.155    -0.33  0.68  0.91
##  Residual             61.235   7.825
## Number of obs: 33460, groups:  Item, 160; Subj, 36
##
## Fixed effects:
##                            Estimate Std. Error         df t value
## (Intercept)              -1.489e-01  3.110e-01  6.751e+01  -0.479
## SYNINCOR                  1.748e+00  4.298e-01  6.113e+01   4.067
## SEMINCOR                  5.959e-01  3.481e-01  8.100e+01   1.712
## AntPost                   9.915e-02  1.696e-01  3.257e+04   0.584
## SYNINCOR:SEMINCOR        -8.220e-01  4.679e-01  7.937e+01  -1.757
## SYNINCOR:AntPost          8.845e-01  2.402e-01  3.257e+04   3.682
## SEMINCOR:AntPost         -1.363e-01  2.421e-01  3.257e+04  -0.563
## SYNINCOR:SEMINCOR:AntPost  8.452e-01  3.423e-01  3.257e+04   2.469
##                          Pr(>|t|)
## (Intercept)              0.633710
## SYNINCOR                 0.000138 ***
## SEMINCOR                 0.090723 .
## AntPost                  0.558895
## SYNINCOR:SEMINCOR        0.082802 .
## SYNINCOR:AntPost         0.000232 ***
## SEMINCOR:AntPost         0.573388
## SYNINCOR:SEMINCOR:AntPost 0.013540 *
## ---
## Signif. codes:  0 '***' 0.001 '**' 0.01 '*' 0.05 '.' 0.1 ' ' 1
##
## Correlation of Fixed Effects:
##                   (Intr) SYNINCOR SEMINCOR AntPst SYNINCOR:SEMINCOR
## SYNINCOR          -0.477
## SEMINCOR          -0.593  0.642
## AntPost           -0.273  0.197    0.244
## SYNINCOR:SEMINCOR  0.426 -0.665   -0.763   -0.181
```

```
## SYNINCOR:AP         0.193 -0.279   -0.172   -0.706  0.257
## SEMINCOR:AP         0.191 -0.138   -0.348   -0.701  0.259
## SYNINCOR:SEMINCOR: -0.135  0.196    0.246    0.496 -0.366
##                    SYNINCOR:A SEMINCOR:
## SYNINCOR
## SEMINCOR
## AntPost
## SYNINCOR:SEMINCOR
## SYNINCOR:AP
## SEMINCOR:AP         0.495
## SYNINCOR:SEMINCOR: -0.702     -0.707
```

Anova wrapper

```
## Analysis of Deviance Table (Type III Wald chisquare tests)
##
## Response: lP600
##               Chisq Df Pr(>Chisq)
## (Intercept)  0.2291  1  0.6321635
## SYN         16.5441  1  4.753e-05 ***
## SEM          2.9309  1  0.0868966 .
## Ant          0.3416  1  0.5588909
## SYN:SEM      3.0865  1  0.0789455 .
## SYN:Ant     13.5546  1  0.0002317 ***
## SEM:Ant      0.3171  1  0.5733837
## SYN:SEM:Ant  6.0978  1  0.0135354 *
## ---
## Signif. codes:  0 '***' 0.001 '**' 0.01 '*' 0.05 '.' 0.1 ' ' 1
```

R-squared (marginal and conditional)

```
##             R2m       R2c
## [1,] 0.01827483 0.1193736
```

Follow-up models splitting by levels of Syntax

Syntax = correct

```
## Linear mixed model fit by maximum likelihood . t-tests use
##   Satterthwaite's method [lmerModLmerTest]
## Formula: lP600 ~ SEM * Ant + (Cond | Subj) + (cCibleP_COR | Item)
##    Data: subset(FWC_Mid, SYN == "COR")
##
##      AIC      BIC   logLik deviance df.resid
## 116598.1 116683.1 -58288.0 116576.1    16709
##
## Scaled residuals:
##     Min      1Q  Median      3Q     Max
## -5.5398 -0.6129  0.0055  0.6227  4.9069
##
## Random effects:
##  Groups   Name        Variance Std.Dev. Corr
##  Item     (Intercept)  1.651   1.285
##           cCibleP_COR 11.743   3.427    -0.21
##  Subj     (Intercept)  2.162   1.470
```

```
##           CondSEM      2.117   1.455    -0.55
##  Residual             60.912   7.805
## Number of obs: 16720, groups:  Item, 160; Subj, 36
##
## Fixed effects:
##                    Estimate Std. Error         df t value Pr(>|t|)
## (Intercept)      -1.475e-01  2.949e-01  5.713e+01  -0.500   0.6189
## SEMINCOR          5.739e-01  2.987e-01  4.957e+01   1.922   0.0604 .
## AntPost           9.915e-02  1.692e-01  1.638e+04   0.586   0.5579
## SEMINCOR:AntPost -1.363e-01  2.415e-01  1.638e+04  -0.565   0.5724
## ---
## Signif. codes:  0 '***' 0.001 '**' 0.01 '*' 0.05 '.' 0.1 ' ' 1
##
## Correlation of Fixed Effects:
##             (Intr) SEMINCOR AntPst
## SEMINCOR    -0.539
## AntPost     -0.287  0.283
## SEMINCOR:AP  0.201 -0.404   -0.701
```

Anova wrapper

```
## Analysis of Deviance Table (Type III Wald chisquare tests)
##
## Response: lP600
##              Chisq Df Pr(>Chisq)
## (Intercept) 0.2501  1    0.61698
## SEM         3.6923  1    0.05467 .
## Ant         0.3434  1    0.55785
## SEM:Ant     0.3187  1    0.57237
## ---
## Signif. codes:  0 '***' 0.001 '**' 0.01 '*' 0.05 '.' 0.1 ' ' 1
```

Syntax = incorrect

```
## Linear mixed model fit by maximum likelihood . t-tests use
##   Satterthwaite's method [lmerModLmerTest]
## Formula: lP600 ~ SEM * Ant + (Cond | Subj) + (cCibleP_COR | Item)
##    Data: subset(FWC_Mid, SYN == "COR")
##
##      AIC      BIC   logLik deviance df.resid
## 116598.1 116683.1 -58288.0 116576.1    16709
##
## Scaled residuals:
##     Min      1Q  Median      3Q     Max
## -5.5398 -0.6129  0.0055  0.6227  4.9069
##
## Random effects:
##  Groups   Name        Variance Std.Dev. Corr
##  Item     (Intercept)  1.651   1.285
##           cCibleP_COR 11.743   3.427    -0.21
##  Subj     (Intercept)  2.162   1.470
##           CondSEM      2.117   1.455    -0.55
##  Residual             60.912   7.805
## Number of obs: 16720, groups:  Item, 160; Subj, 36
##
```

```
## Fixed effects:
##                    Estimate Std. Error         df t value Pr(>|t|)
## (Intercept)      -1.475e-01  2.949e-01  5.713e+01  -0.500   0.6189
## SEMINCOR          5.739e-01  2.987e-01  4.957e+01   1.922   0.0604 .
## AntPost           9.915e-02  1.692e-01  1.638e+04   0.586   0.5579
## SEMINCOR:AntPost -1.363e-01  2.415e-01  1.638e+04  -0.565   0.5724
## ---
## Signif. codes:  0 '***' 0.001 '**' 0.01 '*' 0.05 '.' 0.1 ' ' 1
##
## Correlation of Fixed Effects:
##             (Intr) SEMINCOR AntPst
## SEMINCOR    -0.539
## AntPost     -0.287  0.283
## SEMINCOR:AP  0.201 -0.404   -0.701
```

Anova wrapper

```
## Analysis of Deviance Table (Type III Wald chisquare tests)
##
## Response: lP600
##              Chisq Df Pr(>Chisq)
## (Intercept) 0.2501  1    0.61698
## SEM         3.6923  1    0.05467 .
## Ant         0.3434  1    0.55785
## SEM:Ant     0.3187  1    0.57237
## ---
## Signif. codes:  0 '***' 0.001 '**' 0.01 '*' 0.05 '.' 0.1 ' ' 1
```

Follow-up models splitting by Anteriority levels

Anterior sites

```
## Linear mixed model fit by maximum likelihood . t-tests use
##   Satterthwaite's method [lmerModLmerTest]
## Formula: lP600 ~ SYN * SEM + (Cond | Subj) + (cCibleP_COR | Item)
##    Data: subset(FWC_Mid, Ant == "Ant")
##
##      AIC      BIC   logLik deviance df.resid
## 119867.5 120006.6 -59915.8 119831.5    16712
##
## Scaled residuals:
##     Min      1Q  Median      3Q     Max
## -4.9337 -0.6328 -0.0009  0.6437  5.9553
##
## Random effects:
##  Groups   Name        Variance Std.Dev. Corr
##  Item     (Intercept)  0.7023  0.8381
##           cCibleP_COR  6.3509  2.5201   -0.14
##  Subj     (Intercept)  2.5347  1.5921
##           CondSEM      2.1659  1.4717   -0.49
##           CondSYN      3.7860  1.9458   -0.27  0.77
##           CondSYNSEM   4.7964  2.1901   -0.09  0.59  0.92
##  Residual             74.0152  8.6032
## Number of obs: 16730, groups:  Item, 160; Subj, 36
```

```
##
## Fixed effects:
##                    Estimate Std. Error      df t value Pr(>|t|)
## (Intercept)         -0.1283     0.3070 39.9465  -0.418   0.6781
## SYNINCOR             1.7303     0.3769 34.4377   4.591 5.65e-05 ***
## SEMINCOR             0.5447     0.3120 36.2687   1.746   0.0893 .
## SYNINCOR:SEMINCOR   -0.8111     0.4152 32.8366  -1.953   0.0593 .
## ---
## Signif. codes:  0 '***' 0.001 '**' 0.01 '*' 0.05 '.' 0.1 ' ' 1
##
## Correlation of Fixed Effects:
##             (Intr) SYNINCOR SEMINC
## SYNINCOR    -0.359
## SEMINCOR    -0.523  0.683
## SYNINCOR:SE  0.506 -0.605   -0.820
```

Anova wrapper

```
## Analysis of Deviance Table (Type III Wald chisquare tests)
##
## Response: lP600
##               Chisq Df Pr(>Chisq)
## (Intercept)  0.1748  1    0.67590
## SYN         21.0788  1  4.408e-06 ***
## SEM          3.0478  1    0.08085 .
## SYN:SEM      3.8158  1    0.05077 .
## ---
## Signif. codes:  0 '***' 0.001 '**' 0.01 '*' 0.05 '.' 0.1 ' ' 1
```

Posterior sites

```
## Linear mixed model fit by maximum likelihood . t-tests use
##   Satterthwaite's method [lmerModLmerTest]
## Formula: lP600 ~ SYN * SEM + (Cond | Subj) + (cCibleP_COR | Item)
##    Data: subset(FWC_Mid, Ant == "Post")
##
##      AIC      BIC   logLik deviance df.resid
## 114033.5 114172.6 -56998.8 113997.5    16712
##
## Scaled residuals:
##     Min      1Q  Median      3Q     Max
## -5.9660 -0.6294 -0.0054  0.6272  5.2095
##
## Random effects:
##  Groups   Name        Variance Std.Dev. Corr
##  Item     (Intercept)  0.677   0.8228
##           cCibleP_COR 11.764   3.4299   -0.17
##  Subj     (Intercept)  1.812   1.3461
##           CondSEM      1.704   1.3055   -0.44
##           CondSYN      4.846   2.2014   -0.26  0.79
##           CondSYNSEM   4.801   2.1911   -0.40  0.76  0.92
##  Residual             51.969   7.2089
## Number of obs: 16730, groups:  Item, 160; Subj, 36
##
## Fixed effects:
```

```
##                   Estimate Std. Error       df t value Pr(>|t|)
## (Intercept)       -0.01626    0.26254 42.48385  -0.062    0.951
## SYNINCOR           2.60683    0.40093 34.96653   6.502  1.7e-07 ***
## SEMINCOR           0.40311    0.27100 33.87243   1.488    0.146
## SYNINCOR:SEMINCOR  0.02377    0.35295 30.10222   0.067    0.947
## ---
## Signif. codes:  0 '***' 0.001 '**' 0.01 '*' 0.05 '.' 0.1 ' ' 1
##
## Correlation of Fixed Effects:
##             (Intr) SYNINCOR SEMINC
## SYNINCOR    -0.329
## SEMINCOR    -0.482  0.699
## SYNINCOR:SE  0.250 -0.703   -0.795
```

Anova wrapper

```
## Analysis of Deviance Table (Type III Wald chisquare tests)
##
## Response: lP600
##               Chisq Df Pr(>Chisq)
## (Intercept)  0.0038  1     0.9506
## SYN         42.2758  1  7.927e-11 ***
## SEM          2.2127  1     0.1369
## SYN:SEM      0.0045  1     0.9463
## ---
## Signif. codes:  0 '***' 0.001 '**' 0.01 '*' 0.05 '.' 0.1 ' ' 1
```

At Lateral electrodes

```
## Linear mixed model fit by maximum likelihood . t-tests use
##   Satterthwaite's method [lmerModLmerTest]
## Formula: lP600 ~ SYN * SEM * Ant * Hemi + (Cond | Subj) + (cCibleP_COR |
##     Item)
##    Data: FWC_Lat
##
##       AIC       BIC    logLik  deviance  df.resid
##  791196.6  791486.7 -395568.3  791136.6    117080
##
## Scaled residuals:
##     Min      1Q  Median      3Q     Max
## -6.8560 -0.6161 -0.0029  0.6053  6.0402
##
## Random effects:
##  Groups   Name        Variance Std.Dev. Corr
##  Item     (Intercept)  0.8505  0.9222
##           cCibleP_COR 32.9886  5.7436   0.42
##  Subj     (Intercept)  1.1237  1.0601
##           CondSEM      1.3155  1.1470   -0.60
##           CondSYN      2.4688  1.5712   -0.45  0.83
##           CondSYNSEM   2.6479  1.6272   -0.38  0.74  0.84
##  Residual             49.8496  7.0604
## Number of obs: 117110, groups:  Item, 160; Subj, 36
##
## Fixed effects:
```

```
##                                          Estimate Std. Error         df
## (Intercept)                            -2.371e-01  2.058e-01  5.830e+01
## SYNINCOR                                8.629e-01  2.841e-01  4.409e+01
## SEMINCOR                                4.542e-01  2.209e-01  5.227e+01
## AntPost                                 1.371e-01  1.169e-01  1.166e+05
## Hemiright                              -1.022e-01  1.082e-01  1.166e+05
## SYNINCOR:SEMINCOR                      -5.877e-01  2.991e-01  5.183e+01
## SYNINCOR:AntPost                        1.327e+00  1.656e-01  1.166e+05
## SEMINCOR:AntPost                        5.181e-02  1.668e-01  1.166e+05
## SYNINCOR:Hemiright                      3.345e-01  1.533e-01  1.166e+05
## SEMINCOR:Hemiright                     -1.157e-03  1.545e-01  1.166e+05
## AntPost:Hemiright                       1.181e-01  1.653e-01  1.166e+05
## SYNINCOR:SEMINCOR:AntPost               4.947e-01  2.359e-01  1.166e+05
## SYNINCOR:SEMINCOR:Hemiright             1.396e-01  2.184e-01  1.166e+05
## SYNINCOR:AntPost:Hemiright             -1.209e-01  2.341e-01  1.166e+05
## SEMINCOR:AntPost:Hemiright             -1.184e-01  2.359e-01  1.166e+05
## SYNINCOR:SEMINCOR:AntPost:Hemiright     7.346e-02  3.336e-01  1.166e+05
##                                        t value Pr(>|t|)
## (Intercept)                             -1.152   0.2540
## SYNINCOR                                 3.037   0.0040 **
## SEMINCOR                                 2.056   0.0448 *
## AntPost                                  1.173   0.2410
## Hemiright                               -0.944   0.3450
## SYNINCOR:SEMINCOR                       -1.965   0.0548 .
## SYNINCOR:AntPost                         8.014 1.12e-15 ***
## SEMINCOR:AntPost                         0.311   0.7562
## SYNINCOR:Hemiright                       2.182   0.0291 *
## SEMINCOR:Hemiright                      -0.007   0.9940
## AntPost:Hemiright                        0.715   0.4749
## SYNINCOR:SEMINCOR:AntPost                2.097   0.0360 *
## SYNINCOR:SEMINCOR:Hemiright              0.639   0.5226
## SYNINCOR:AntPost:Hemiright              -0.516   0.6055
## SEMINCOR:AntPost:Hemiright              -0.502   0.6158
## SYNINCOR:SEMINCOR:AntPost:Hemiright      0.220   0.8257
## ---
## Signif. codes:  0 '***' 0.001 '**' 0.01 '*' 0.05 '.' 0.1 ' ' 1

##
## Correlation matrix not shown by default, as p = 16 > 12.
## Use print(x, correlation=TRUE)  or
##   vcov(x)     if you need it
```

Anova wrapper

```
## Analysis of Deviance Table (Type III Wald chisquare tests)
##
## Response: lP600
##                   Chisq Df Pr(>Chisq)
## (Intercept)       1.3274  1   0.249262
## SYN               9.2259  1   0.002386 **
## SEM               4.2266  1   0.039795 *
## Ant               1.3750  1   0.240951
## Hemi              0.8918  1   0.344988
## SYN:SEM           3.8599  1   0.049453 *
## SYN:Ant          64.2236  1  1.111e-15 ***
```

```
## SEM:Ant           0.0964  1   0.756156
## SYN:Hemi          4.7628  1   0.029080 *
## SEM:Hemi          0.0001  1   0.994025
## Ant:Hemi          0.5106  1   0.474870
## SYN:SEM:Ant       4.3982  1   0.035976 *
## SYN:SEM:Hemi      0.4088  1   0.522568
## SYN:Ant:Hemi      0.2667  1   0.605538
## SEM:Ant:Hemi      0.2518  1   0.615777
## SYN:SEM:Ant:Hemi  0.0485  1   0.825691
## ---
## Signif. codes:  0 '***' 0.001 '**' 0.01 '*' 0.05 '.' 0.1 ' ' 1
```

R-squared (marginal and conditional)

```
##            R2m        R2c
## [1,] 0.0168678 0.09448006
```

Follow-up models splitting by levels of Syntax

Syntax = correct

```
## Linear mixed model fit by maximum likelihood . t-tests use
##   Satterthwaite's method [lmerModLmerTest]
## Formula: lP600 ~ SEM * Ant * Hemi + (Cond | Subj) + (cCibleP_COR | Item)
##    Data: subset(FWC_Lat, SYN == "COR")
##
##       AIC       BIC    logLik  deviance  df.resid
##  393280.0  393414.7 -196625.0  393250.0     58505
##
## Scaled residuals:
##     Min      1Q  Median      3Q     Max
## -6.1263 -0.6111  0.0012  0.6045  6.3014
##
## Random effects:
##  Groups   Name        Variance Std.Dev. Corr
##  Item     (Intercept)  1.246   1.116
##           cCibleP_COR 32.628   5.712    0.10
##  Subj     (Intercept)  1.149   1.072
##           CondSEM      1.359   1.166    -0.61
##  Residual             47.896   6.921
## Number of obs: 58520, groups:  Item, 160; Subj, 36
##
## Fixed effects:
##                              Estimate Std. Error         df t value
## (Intercept)                -2.676e-01  2.166e-01  6.705e+01  -1.235
## SEMINCOR                    4.689e-01  2.227e-01  5.059e+01   2.105
## AntPost                     1.371e-01  1.146e-01  5.807e+04   1.196
## Hemiright                  -1.022e-01  1.061e-01  5.807e+04  -0.963
## SEMINCOR:AntPost            5.181e-02  1.635e-01  5.807e+04   0.317
## SEMINCOR:Hemiright         -1.157e-03  1.514e-01  5.807e+04  -0.008
## AntPost:Hemiright           1.181e-01  1.620e-01  5.807e+04   0.729
## SEMINCOR:AntPost:Hemiright -1.184e-01  2.313e-01  5.807e+04  -0.512
##                            Pr(>|t|)
## (Intercept)                  0.2211
```

```
## SEMINCOR                      0.0403 *
## AntPost                       0.2316
## Hemiright                     0.3353
## SEMINCOR:AntPost              0.7514
## SEMINCOR:Hemiright            0.9939
## AntPost:Hemiright             0.4660
## SEMINCOR:AntPost:Hemiright   0.6087
## ---
## Signif. codes:  0 '***' 0.001 '**' 0.01 '*' 0.05 '.' 0.1 ' ' 1
##
## Correlation of Fixed Effects:
##              (Intr) SEMINCOR AntPst Hmrght SEMINCOR:AnP SEMINCOR:H AntP:H
## SEMINCOR     -0.557
## AntPost      -0.227  0.220
## Hemiright    -0.245  0.238    0.463
## SEMINCOR:AnP  0.159 -0.315   -0.701 -0.324
## SEMINCOR:Hm   0.172 -0.340   -0.324 -0.701  0.463
## AntPst:Hmrg   0.160 -0.156   -0.707 -0.655  0.495        0.459
## SEMINCOR:AP: -0.112  0.223    0.495  0.459 -0.707       -0.655     -0.701
```

Anova wrapper

```
## Analysis of Deviance Table (Type III Wald chisquare tests)
##
## Response: lP600
##               Chisq Df Pr(>Chisq)
## (Intercept)  1.5253  1    0.21682
## SEM          4.4316  1    0.03528 *
## Ant          1.4311  1    0.23158
## Hemi         0.9282  1    0.33533
## SEM:Ant      0.1004  1    0.75139
## SEM:Hemi     0.0001  1    0.99390
## Ant:Hemi     0.5315  1    0.46600
## SEM:Ant:Hemi 0.2621  1    0.60867
## ---
## Signif. codes:  0 '***' 0.001 '**' 0.01 '*' 0.05 '.' 0.1 ' ' 1
```

Syntax = incorrect

```
## Linear mixed model fit by maximum likelihood . t-tests use
##   Satterthwaite's method [lmerModLmerTest]
## Formula: lP600 ~ SEM * Ant + (Cond | Subj) + (cCibleP_COR | Item)
##    Data: subset(FWC_Lat, SYN == "INCOR")
##
##       AIC       BIC    logLik  deviance  df.resid
##  493601.5  493702.4 -246789.7  493579.5     71134
##
## Scaled residuals:
##     Min      1Q  Median      3Q     Max
## -6.2198 -0.6006  0.0067  0.6089  6.6195
##
## Random effects:
##  Groups   Name        Variance Std.Dev. Corr
##  Item     (Intercept)   1.7604  1.3268
##           cCibleP_COR 106.6002 10.3247  0.60
```

```
##  Subj     (Intercept)   2.4324  1.5596
##           CondSYNSEM    0.9114  0.9547  -0.08
##  Residual              59.5948  7.7198
## Number of obs: 71145, groups:  Item, 160; Subj, 36
##
## Fixed effects:
##                    Estimate Std. Error         df t value Pr(>|t|)
## (Intercept)       7.242e-01  2.827e-01  4.658e+01   2.562   0.0137 *
## SEMINCOR         -3.911e-02  1.811e-01  3.781e+01  -0.216   0.8302
## AntPost           5.619e-01  8.155e-02  7.074e+04   6.890 5.61e-12 ***
## SEMINCOR:AntPost  2.275e-01  1.160e-01  7.074e+04   1.961   0.0498 *
## ---
## Signif. codes:  0 '***' 0.001 '**' 0.01 '*' 0.05 '.' 0.1 ' ' 1
##
## Correlation of Fixed Effects:
##             (Intr) SEMINCOR AntPst
## SEMINCOR    -0.140
## AntPost     -0.153  0.238
## SEMINCOR:AP  0.107 -0.339   -0.703
```

Anova wrapper

```
## Analysis of Deviance Table (Type III Wald chisquare tests)
##
## Response: lP600
##               Chisq Df Pr(>Chisq)
## (Intercept)  6.5636  1    0.01041 *
## SEM          0.0466  1    0.82903
## Ant         47.4762  1  5.568e-12 ***
## SEM:Ant      3.8470  1    0.04984 *
## ---
## Signif. codes:  0 '***' 0.001 '**' 0.01 '*' 0.05 '.' 0.1 ' ' 1
```

Follow-up models splitting by Anteriority levels

Anterior sites

```
## Linear mixed model fit by maximum likelihood . t-tests use
##   Satterthwaite's method [lmerModLmerTest]
## Formula: lP600 ~ SYN * SEM * Hemi + (Cond | Subj) + (cCibleP_COR | Item)
##    Data: subset(FWC_Lat, Ant == "Ant")
##
##       AIC       BIC    logLik  deviance  df.resid
##  458729.7  458930.1 -229342.8  458685.7     66898
##
## Scaled residuals:
##     Min      1Q  Median      3Q     Max
## -6.4446 -0.6129 -0.0053  0.6081  5.7381
##
## Random effects:
##  Groups   Name        Variance Std.Dev. Corr
##  Item     (Intercept)  0.8007  0.8948
##           cCibleP_COR 19.8646  4.4570   0.19
##  Subj     (Intercept)  1.4111  1.1879
```

```
##            CondSEM       1.4035  1.1847   -0.50
##            CondSYN       2.0450  1.4300   -0.42  0.73
##            CondSYNSEM    2.7447  1.6567   -0.21  0.63  0.73
##  Residual              54.8388  7.4053
## Number of obs: 66920, groups:  Item, 160; Subj, 36
##
## Fixed effects:
##                               Estimate Std. Error         df t value
## (Intercept)                 -2.691e-01  2.273e-01  5.218e+01  -1.184
## SYNINCOR                     8.602e-01  2.653e-01  4.225e+01   3.243
## SEMINCOR                     4.796e-01  2.295e-01  4.794e+01   2.089
## Hemiright                   -1.022e-01  1.135e-01  6.633e+04  -0.900
## SYNINCOR:SEMINCOR           -5.691e-01  3.221e-01  4.487e+01  -1.767
## SYNINCOR:Hemiright           3.345e-01  1.608e-01  6.633e+04   2.081
## SEMINCOR:Hemiright          -1.157e-03  1.620e-01  6.633e+04  -0.007
## SYNINCOR:SEMINCOR:Hemiright  1.396e-01  2.290e-01  6.633e+04   0.610
##                             Pr(>|t|)
## (Intercept)                  0.24180
## SYNINCOR                     0.00231 **
## SEMINCOR                     0.04201 *
## Hemiright                    0.36792
## SYNINCOR:SEMINCOR            0.08402 .
## SYNINCOR:Hemiright           0.03746 *
## SEMINCOR:Hemiright           0.99430
## SYNINCOR:SEMINCOR:Hemiright  0.54212
## ---
## Signif. codes:  0 '***' 0.001 '**' 0.01 '*' 0.05 '.' 0.1 ' ' 1
##
## Correlation of Fixed Effects:
##                    (Intr) SYNINCOR SEMINCOR Hmrght SYNINCOR:SEMINCOR
## SYNINCOR           -0.444
## SEMINCOR           -0.500  0.679
## Hemiright          -0.250  0.214    0.247
## SYNINCOR:SEMINCOR   0.475 -0.666   -0.716   -0.176
## SYNINCOR:Hm         0.176 -0.303   -0.175   -0.706  0.250
## SEMINCOR:Hm         0.175 -0.150   -0.353   -0.701  0.251
## SYNINCOR:SEMINCOR: -0.124  0.213    0.250    0.496 -0.356
##                    SYNINCOR:H SEMINCOR:
## SYNINCOR
## SEMINCOR
## Hemiright
## SYNINCOR:SEMINCOR
## SYNINCOR:Hm
## SEMINCOR:Hm         0.495
## SYNINCOR:SEMINCOR: -0.702     -0.707
```

Anova wrapper

```
## Analysis of Deviance Table (Type III Wald chisquare tests)
##
## Response: lP600
##                Chisq Df Pr(>Chisq)
## (Intercept)   1.4017  1   0.236439
## SYN          10.5151  1   0.001184 **
## SEM           4.3651  1   0.036682 *
```

```
## Hemi          0.8107  1   0.367922
## SYN:SEM       3.1224  1   0.077223 .
## SYN:Hemi      4.3295  1   0.037457 *
## SEM:Hemi      0.0001  1   0.994304
## SYN:SEM:Hemi  0.3716  1   0.542118
## ---
## Signif. codes:  0 '***' 0.001 '**' 0.01 '*' 0.05 '.' 0.1 ' ' 1
```

Posterior sites

```
## Linear mixed model fit by maximum likelihood . t-tests use
##   Satterthwaite's method [lmerModLmerTest]
## Formula: lP600 ~ SYN * SEM * Hemi + (Cond | Subj) + (cCibleP_COR | Item)
##    Data: subset(FWC_Lat, Ant == "Post")
##
##       AIC       BIC    logLik  deviance  df.resid
##  330422.3  330616.4 -165189.2  330378.3     50168
##
## Scaled residuals:
##     Min      1Q  Median      3Q     Max
## -6.1724 -0.6250 -0.0015  0.6143  5.6717
##
## Random effects:
##  Groups   Name        Variance Std.Dev. Corr
##  Item     (Intercept)  0.8362  0.9145
##           cCibleP_COR 29.4829  5.4298   0.18
##  Subj     (Intercept)  1.3942  1.1808
##           CondSEM      1.7838  1.3356   -0.60
##           CondSYN      4.1343  2.0333   -0.43  0.78
##           CondSYNSEM   4.2749  2.0676   -0.55  0.75  0.87
##  Residual             41.5884  6.4489
## Number of obs: 50190, groups:  Item, 160; Subj, 36
##
## Fixed effects:
##                              Estimate Std. Error         df t value
## (Intercept)                -6.770e-02  2.276e-01  5.349e+01  -0.297
## SYNINCOR                    2.204e+00  3.586e-01  3.924e+01   6.145
## SEMINCOR                    4.738e-01  2.518e-01  4.295e+01   1.882
## Hemiright                   1.593e-02  1.141e-01  4.968e+04   0.140
## SYNINCOR:SEMINCOR          -1.381e-01  3.290e-01  4.259e+01  -0.420
## SYNINCOR:Hemiright          2.136e-01  1.616e-01  4.968e+04   1.321
## SEMINCOR:Hemiright         -1.196e-01  1.629e-01  4.968e+04  -0.734
## SYNINCOR:SEMINCOR:Hemiright  2.131e-01  2.303e-01  4.968e+04   0.925
##                            Pr(>|t|)
## (Intercept)                  0.7673
## SYNINCOR                   3.18e-07 ***
## SEMINCOR                     0.0667 .
## Hemiright                    0.8890
## SYNINCOR:SEMINCOR            0.6767
## SYNINCOR:Hemiright           0.1864
## SEMINCOR:Hemiright           0.4630
## SYNINCOR:SEMINCOR:Hemiright   0.3549
## ---
## Signif. codes:  0 '***' 0.001 '**' 0.01 '*' 0.05 '.' 0.1 ' ' 1
```

```
##
## Correlation of Fixed Effects:
##                    (Intr) SYNINCOR SEMINCOR Hmrght SYNINCOR:SEMINCOR
## SYNINCOR           -0.437
## SEMINCOR           -0.580  0.726
## Hemiright          -0.251  0.159    0.227
## SYNINCOR:SEMINCOR   0.330 -0.722   -0.775   -0.173
## SYNINCOR:Hm         0.177 -0.225   -0.160   -0.706  0.246
## SEMINCOR:Hm         0.176 -0.112   -0.323   -0.701  0.248
## SYNINCOR:SEMINCOR: -0.124  0.158    0.229    0.496 -0.350
##                    SYNINCOR:H SEMINCOR:
## SYNINCOR
## SEMINCOR
## Hemiright
## SYNINCOR:SEMINCOR
## SYNINCOR:Hm
## SEMINCOR:Hm         0.495
## SYNINCOR:SEMINCOR: -0.702     -0.707
```

Anova wrapper

```
## Analysis of Deviance Table (Type III Wald chisquare tests)
##
## Response: lP600
##                Chisq Df Pr(>Chisq)
## (Intercept)   0.0885  1    0.76614
## SYN          37.7588  1  8.005e-10 ***
## SEM           3.5410  1    0.05987 .
## Hemi          0.0195  1    0.88902
## SYN:SEM       0.1763  1    0.67455
## SYN:Hemi      1.7457  1    0.18642
## SEM:Hemi      0.5387  1    0.46299
## SYN:SEM:Hemi  0.8560  1    0.35486
## ---
## Signif. codes:  0 '***' 0.001 '**' 0.01 '*' 0.05 '.' 0.1 ' ' 1
```

Follow-up models splitting by Hemisphere levels

Right hemisphere

```
## Linear mixed model fit by maximum likelihood . t-tests use
##   Satterthwaite's method [lmerModLmerTest]
## Formula: lP600 ~ SYN * SEM * Ant + (Cond | Subj) + (cCibleP_COR | Item)
##    Data: subset(FWC_Lat, Hemi == "right")
##
##       AIC       BIC    logLik  deviance  df.resid
##  396474.0  396671.5 -198215.0  396430.0     58533
##
## Scaled residuals:
##     Min      1Q  Median      3Q     Max
## -6.2159 -0.6142 -0.0027  0.6049  6.0182
##
## Random effects:
##  Groups   Name        Variance Std.Dev. Corr
```

```
##  Item     (Intercept)  0.6075  0.7794
##           cCibleP_COR 20.5839  4.5370   0.26
##  Subj     (Intercept)  1.4577  1.2073
##           CondSEM      1.6821  1.2970   -0.62
##           CondSYN      2.8656  1.6928   -0.50  0.82
##           CondSYNSEM   2.9346  1.7131   -0.47  0.74  0.85
##  Residual             50.3823  7.0980
## Number of obs: 58555, groups:  Item, 160; Subj, 36
##
## Fixed effects:
##                            Estimate Std. Error         df t value
## (Intercept)                 -0.3268     0.2260    47.7059  -1.446
## SYNINCOR                     1.1885     0.3036    39.3507   3.915
## SEMINCOR                     0.4404     0.2437    42.6849   1.808
## AntPost                      0.2552     0.1175 57989.3049   2.172
## SYNINCOR:SEMINCOR           -0.4421     0.3241    41.2720  -1.364
## SYNINCOR:AntPost             1.2058     0.1664 57989.3052   7.245
## SEMINCOR:AntPost            -0.0666     0.1677 57989.3049  -0.397
## SYNINCOR:SEMINCOR:AntPost    0.5681     0.2371 57989.3051   2.396
##                           Pr(>|t|)
## (Intercept)               0.154764
## SYNINCOR                  0.000349 ***
## SEMINCOR                  0.077734 .
## AntPost                   0.029889 *
## SYNINCOR:SEMINCOR         0.179919
## SYNINCOR:AntPost          4.38e-13 ***
## SEMINCOR:AntPost          0.691321
## SYNINCOR:SEMINCOR:AntPost 0.016583 *
## ---
## Signif. codes:  0 '***' 0.001 '**' 0.01 '*' 0.05 '.' 0.1 ' ' 1
##
## Correlation of Fixed Effects:
##                     (Intr) SYNINCOR SEMINCOR AntPst SYNINCOR:SEMINCOR
## SYNINCOR            -0.505
## SEMINCOR            -0.603  0.757
## AntPost             -0.223  0.166    0.207
## SYNINCOR:SEMINCOR    0.474 -0.750   -0.804   -0.155
## SYNINCOR:AP          0.157 -0.235   -0.146   -0.706  0.220
## SEMINCOR:AP          0.156 -0.116   -0.295   -0.701  0.222
## SYNINCOR:SEMINCOR: -0.110  0.165    0.209    0.496 -0.314
##                     SYNINCOR:A SEMINCOR:
## SYNINCOR
## SEMINCOR
## AntPost
## SYNINCOR:SEMINCOR
## SYNINCOR:AP
## SEMINCOR:AP          0.495
## SYNINCOR:SEMINCOR: -0.702     -0.707
```

Anova wrapper

```
## Analysis of Deviance Table (Type III Wald chisquare tests)
##
## Response: lP600
##                Chisq Df Pr(>Chisq)
```

```
## (Intercept)  2.0904  1    0.14823
## SYN         15.3286  1  9.034e-05 ***
## SEM          3.2671  1    0.07068 .
## Ant          4.7159  1    0.02989 *
## SYN:SEM      1.8609  1    0.17253
## SYN:Ant     52.4898  1  4.325e-13 ***
## SEM:Ant      0.1577  1    0.69132
## SYN:SEM:Ant  5.7403  1    0.01658 *
## ---
## Signif. codes:  0 '***' 0.001 '**' 0.01 '*' 0.05 '.' 0.1 ' ' 1
```

Left hemisphere

```
## Linear mixed model fit by maximum likelihood . t-tests use
##   Satterthwaite's method [lmerModLmerTest]
## Formula: lP600 ~ SYN * SEM * Ant + (Cond | Subj) + (cCibleP_COR + Cond |
##     Item)
##    Data: subset(FWC_Lat, Hemi == "left")
##
##       AIC       BIC    logLik  deviance  df.resid
##  394157.6  394462.8 -197044.8  394089.6     58521
##
## Scaled residuals:
##     Min      1Q  Median      3Q     Max
## -6.8150 -0.6142 -0.0077  0.6040  6.1845
##
## Random effects:
##  Groups   Name        Variance Std.Dev. Corr
##  Item     (Intercept)  2.1821  1.4772
##           cCibleP_COR 10.0537  3.1708   -0.22
##           CondSEM      3.7891  1.9466   -0.64  0.38
##           CondSYN      3.5316  1.8793   -0.68  0.13  0.48
##           CondSYNSEM   4.1140  2.0283   -0.70  0.35  0.47  0.48
##  Subj     (Intercept)  0.9908  0.9954
##           CondSEM      1.1841  1.0882   -0.59
##           CondSYN      2.1649  1.4713   -0.43  0.83
##           CondSYNSEM   2.5038  1.5823   -0.31  0.68  0.87
##  Residual             47.8671  6.9186
## Number of obs: 58555, groups:  Item, 160; Subj, 36
##
## Fixed effects:
##                              Estimate Std. Error         df t value
## (Intercept)                -2.952e-01  2.196e-01  7.649e+01  -1.344
## SYNINCOR                    9.166e-01  3.071e-01  6.453e+01   2.985
## SEMINCOR                    4.528e-01  2.599e-01  8.788e+01   1.742
## AntPost                     1.371e-01  1.145e-01  5.762e+04   1.197
## SYNINCOR:SEMINCOR          -6.319e-01  3.610e-01  8.873e+01  -1.750
## SYNINCOR:AntPost            1.327e+00  1.622e-01  5.762e+04   8.178
## SEMINCOR:AntPost            5.181e-02  1.635e-01  5.762e+04   0.317
## SYNINCOR:SEMINCOR:AntPost  4.947e-01  2.311e-01  5.762e+04   2.140
##                            Pr(>|t|)
## (Intercept)                  0.1829
## SYNINCOR                     0.0040 **
## SEMINCOR                     0.0850 .
```

```
## AntPost                      0.2314
## SYNINCOR:SEMINCOR            0.0835 .
## SYNINCOR:AntPost           2.94e-16 ***
## SEMINCOR:AntPost             0.7513
## SYNINCOR:SEMINCOR:AntPost   0.0323 *
## ---
## Signif. codes:  0 '***' 0.001 '**' 0.01 '*' 0.05 '.' 0.1 ' ' 1
##
## Correlation of Fixed Effects:
##                   (Intr) SYNINCOR SEMINCOR AntPst SYNINCOR:SEMINCOR
## SYNINCOR          -0.526
## SEMINCOR          -0.619  0.673
## AntPost           -0.224  0.160    0.189
## SYNINCOR:SEMINCOR  0.478 -0.673   -0.768   -0.136
## SYNINCOR:AP        0.158 -0.226   -0.133   -0.706  0.193
## SEMINCOR:AP        0.157 -0.112   -0.270   -0.701  0.194
## SYNINCOR:SEMINCOR: -0.111  0.159    0.191    0.496 -0.274
##                   SYNINCOR:A SEMINCOR:
## SYNINCOR
## SEMINCOR
## AntPost
## SYNINCOR:SEMINCOR
## SYNINCOR:AP
## SEMINCOR:AP        0.495
## SYNINCOR:SEMINCOR: -0.702     -0.707
```

Anova wrapper

```
## Analysis of Deviance Table (Type III Wald chisquare tests)
##
## Response: lP600
##               Chisq Df Pr(>Chisq)
## (Intercept)  1.8068  1   0.178894
## SYN          8.9095  1   0.002837 **
## SEM          3.0354  1   0.081469 .
## Ant          1.4320  1   0.231443
## SYN:SEM      3.0641  1   0.080039 .
## SYN:Ant     66.8835  1   2.88e-16 ***
## SEM:Ant      0.1004  1   0.751321
## SYN:SEM:Ant  4.5804  1   0.032340 *
## ---
## Signif. codes:  0 '***' 0.001 '**' 0.01 '*' 0.05 '.' 0.1 ' ' 1
```
