# Supplementary material for: Verbing nouns and nouning verbs: Using a balanced design provides ERP evidence against “syntax-first” approaches to sentence processing
Source: PLoS One. 2020 Mar 13;15(3):e0229169. doi: 10.1371/journal.pone.0229169 (PMC7069651; doi:10.1371/journal.pone.0229169)
Supplement: S1 Fig — These maps illustrate the syntactic category violation effects (violation minus correct) in 100 ms time windows between 300 ms and 1300 ms post onset of the underlined critical words (from left to right). (DOCX) [file pone.0229169.s003.docx]

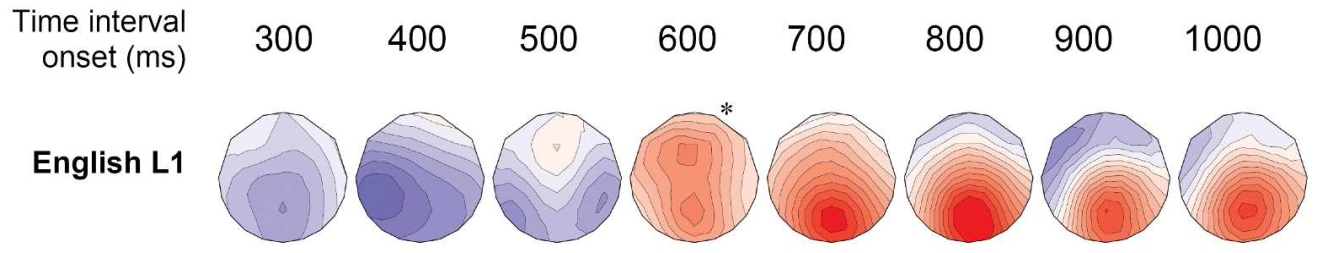

Supplementary Fig 1. Voltage Maps Comparing SCV Effects in native English speakers. These maps illustrate the syntactic category violation effects (violation minus correct) in 100 ms time windows between 300 ms and 1300 ms post onset of the underlined critical words (from left to right).
