# Supplementary material for: Verbing nouns and nouning verbs: Using a balanced design provides ERP evidence against “syntax-first” approaches to sentence processing
Source: PLoS One. 2020 Mar 13;15(3):e0229169. doi: 10.1371/journal.pone.0229169 (PMC7069651; doi:10.1371/journal.pone.0229169)
Supplement: S1 Table — (DOCX) [file pone.0229169.s004.docx]

Supplementary Table 1. Paired t-test results comparing control verbs and transitive verbs.

| Lexical dimension | *Mean* (*SD*) | | Paired t-tests results | |
| --- | --- | --- | --- | --- |
|  | Control verbs  (e.g. *oser*) | Transitive verbs  (e.g. *ôter*) | t(df) | *p* value |
| Num. Phonemes | 3.8 (1.23) | 3.8 (1.32) | < .001 | 1 |
| Num. Characters | 6.45 (1.36) | 6.20 (1.51) | 1.097 | .287 |
| Frequency* | 2.35 (0.94) | 2.80 (1.24) | -1.360 | .190 |

*All lexical measures were retrieved from Lexique.org [S1]. Frequencies are log-transformed values from lemma frequencies for French film subtitles. Syllable structure (not in table?) and length are based on Québec French phonology

**Reference**

S1. New B, Pallier C, Ferrand L, Matos R. Une base de données lexicales du français contemporain sur internet : LEXIQUE. L’Année Psychologique, 2001;101:447-462.
