# Supplementary material for: Verbing nouns and nouning verbs: Using a balanced design provides ERP evidence against “syntax-first” approaches to sentence processing
Source: PLoS One. 2020 Mar 13;15(3):e0229169. doi: 10.1371/journal.pone.0229169 (PMC7069651; doi:10.1371/journal.pone.0229169)
Supplement: S2 Table — (DOCX) [file pone.0229169.s005.docx]

Supplementary Table 2. Paired t-test results comparing target verbs and nouns.

| Lexical dimension | *Mean*(*SD*) | | Paired t-tests results | |
| --- | --- | --- | --- | --- |
|  | Target verbs  (e.g. *plaquer*) | Target nouns  (e.g. *crapaud*) | t(df) | *p* value |
| Syllable length | 2.10, SDV=.04 | 2.09, SDN=.04 | -0.706 | .438 |
| Num. phonemes | 5.12, SDV=.08 | 5.06, SDN=.08 | -1.164 | .246 |
| Num. characters | 6.52, SDV=.09 | 6.94, SDN=.08 | 4.784 | <.001 |
| Frequency | 1.31, SDV=.77 | 1.21, SDN=.74 | -2.122 | .103 |
