# Supplementary material for: Verbing nouns and nouning verbs: Using a balanced design provides ERP evidence against “syntax-first” approaches to sentence processing
Source: PLoS One. 2020 Mar 13;15(3):e0229169. doi: 10.1371/journal.pone.0229169 (PMC7069651; doi:10.1371/journal.pone.0229169)
Supplement: S3 Table — (DOCX) [file pone.0229169.s006.docx]

Supplementary Table 3. Priming and context effects on target predictability.

| Experimental measure | *Mean*(*SD*) | | Paired t-tests results | |
| --- | --- | --- | --- | --- |
|  | Target verbs  (e.g. *plaquer*) | Target nouns  (e.g. *crapaud*) | t(df=159) | *p* value |
| Relatedness between prime and target* | 3.25, SDV=1.04 | 3.39, SDN=1.10 | 1.353 | .18 |
| Cloze probability** | .10, SDV=.18 | .17, SDN=.26 | 2.774 | .006 |

* To test the degree of priming, we presented pairs of primes and targets (e.g. ‘hockey’ and ‘tackle’) to sixty different French speakers through online questionnaires, and asked them to rate their relatedness on a scale from 1 (not related) to 5 (very related).

** We removed the target word from all 160 sentences pairs in the correct condition, and divided them into four lists using a Latin square design. Forty French-speaking participants were asked to complete the sentences with what they thought was the most appropriate word. We then calculated the probability of the target words to be used based on sentence-completion results on a range from 0 – the target was never employed as best completion– to 1 – the target was always employed as completion.
