# Supplementary material for: Verbing nouns and nouning verbs: Using a balanced design provides ERP evidence against “syntax-first” approaches to sentence processing
Source: PLoS One. 2020 Mar 13;15(3):e0229169. doi: 10.1371/journal.pone.0229169 (PMC7069651; doi:10.1371/journal.pone.0229169)
Supplement: S1 Materials — (DOCX) [file pone.0229169.s007.docx]

**S1 Materials.** <https://osf.io/k7vxp/>
